# Diet selection in the Coyote *Canis latrans*

**DOI:** 10.1093/jmammal/gyad094

**Published:** 2023-11-04

**Authors:** Matt W Hayward, Carl D Mitchell, Jan F Kamler, Paul Rippon, David R Heit, Vilis Nams, Robert A Montgomery

**Affiliations:** Conservation Science Research Group, College of Engineering, Science and the Environment, University of Newcastle, Callaghan, New South Wales 2207, Australia; Mammal Research Institute, University of Pretoria, Tshwane X001, South Africa; Centre for African Conservation Ecology, Nelson Mandela University, Port Elizabeth 6213, South Africa; Wayan, Idaho 83285, USA; Wildlife Conservation Research Unit, The Recanati-Kaplan Centre, Department of Biology, University of Oxford, Oxford OX13 5QL, United Kingdom; School of Information and Physical Sciences, University of Newcastle, Callaghan, New South Wales 2207, Australia; University of New Hampshire, Department of Natural Resources and the Environment, Durham, New Hampshire 03824, USA; Department of Plant, Food and Environmental Sciences, Agricultural Campus, Dalhousie University, Truro, Nova Scotia B2N 5E3, Canada; Wildlife Conservation Research Unit, The Recanati-Kaplan Centre, Department of Biology, University of Oxford, Oxford OX13 5QL, United Kingdom

**Keywords:** *Canis latrans*, Coyote, diet, Jacobs’ index, predator–prey, prey preferences

## Abstract

The Coyote (*Canis latrans*) is one of the most studied species in North America with at least 445 papers on its diet alone. While this research has yielded excellent reviews of what coyotes eat, it has been inadequate to draw deeper conclusions because no synthesis to date has considered prey availability. We accounted for prey availability by investigating the prey selection of coyotes across its distribution using the traditional Jacobs’ index method, as well as the new iterative preference averaging (IPA) method on scats and biomass. We found that coyotes selected for Dall’s Sheep (*Ovis dalli*), White-tailed Deer (*Odocoileus virginianus*), Eastern Cottontail Rabbit (*Sylvilagus floridanus*), and California Vole (*Microtus californicus*), which yielded a predator-to-preferred prey mass ratio of 1:2. We also found that coyotes avoided preying on other small mammals, including carnivorans and arboreal species. There was strong concordance between the traditional and IPA method on scats, but this pattern was weakened when biomass was considered. General linear models revealed that coyotes preferred to prey upon larger species that were riskier to hunt, reflecting their ability to hunt in groups, and were least likely to hunt solitary species. Coyotes increasingly selected Mule Deer (*O. hemionus*) and Snowshoe Hare (*Lepus americanus*) at higher latitudes, whereas Black-tailed Jackrabbit (*L. californicus*) were increasingly selected toward the tropics. Mule Deer were increasingly selected at higher coyote densities, while Black-tailed Jackrabbit were increasingly avoided at higher coyote densities. Coyote predation could constrain the realized niche of prey species at the distributional limits of the predator through their increased efficiency of predation reflected in increased prey selection values. These results are integral to improved understandings of Coyote ecology and can inform predictive analyses allowing for spatial variation, which ultimately will lead to better understandings about the ecological role of the coyote across different ecosystems.

## Teaser

Coyotes preferentially prey on Dall’s Sheep, White-tailed Deer, Eastern Cottontail Rabbit, and California Vole, while they avoid depredating small mammals, carnivorans, and arboreal species. These prey preferences reflect their predatory behavioral flexibility in being able to hunt alone or in groups. Understanding predator–prey preferences enables us to begin predicting their responses to variation in bottom-up dietary resources.

## Abstract (es)

El coyote (*Canis latrans*) es una de las especies más estudiadas en América del Norte con al menos 445 artículos solo sobre su dieta. Si bien esta investigación ha producido excelentes revisiones de lo que comen los coyotes, no ha sido adecuada para sacar conclusiones más profundas porque ninguna síntesis hasta la fecha ha considerado la disponibilidad de presas. Tomamos en cuenta la disponibilidad de presas al investigar la selección de presas de los coyotes a lo largo de la distribución de la especie utilizando el método tradicional del índice de Jacobs, así como el nuevo método iterativo de promedio de preferencia sobre excrementos y biomasa. Descubrimos que los coyotes seleccionados para la oveja de Dall (*Ovis dalli*), el venado de cola blanca (*Odocoileus virginianus*), los conejos de rabo blanco del este (*Sylvilagus floridanus*) y los campañoles de California (*Microtus californicus*), produjeron una proporción de masa de depredador a presa preferida de 1:2. También encontramos que los coyotes evitaban depredar a otros mamíferos pequeños, incluidos los carnívoros y las especies arbóreas. Hubo una fuerte concordancia entre el método de promedio de preferencia tradicional e iterativo en los excrementos, pero este patrón se debilitó cuando se consideró la biomasa. Los modelos lineales generales revelaron que los coyotes preferían cazar especies más grandes que eran más riesgosas de cazar, lo que reflejaba su capacidad para cazar en grupos, y era menos probable que cazaran especies solitarias. Los coyotes seleccionaron cada vez más al venado bura (*O. hemionus*) y la liebre con raquetas de nieve (*Lepus americanus*) en latitudes más altas, mientras que la liebre de cola negra (*L. californicus*) fue seleccionada cada vez más hacia los trópicos. El venado bura se seleccionaba cada vez más en densidades más altas de coyotes, mientras que la liebre de cola negra se evitaba cada vez más en densidades más altas de coyotes. La depredación de los coyotes podría restringir el nicho realizado de las especies de presa en los límites de distribución de las especies de depredadores a través de su mayor eficiencia de depredación reflejada en mayores valores de selección de presas. Estos resultados son parte integral de una mejor comprensión de la ecología del coyote y pueden informar análisis predictivos que permitan la variación espacial, lo que en última instancia conducirá a una mejor comprensión sobre el papel ecológico del coyote en diferentes ecosistemas.

Coyotes (*Canis latrans*) are one of the few Carnivoran species to have benefited from humans, having greatly expanded their range since 1500 (Hody and Kays 2018), including a 40% expansion since the 1950s (Laliberte and Ripple 2004). This has occurred despite the species being heavily persecuted for their real or perceived predation on livestock (LeSher 2020). The ability of coyotes to thrive despite human persecution reflects comparatively high reproductive rate and ecological plasticity. Coyotes can hunt in groups or as individuals across their range in North America (Bowen 1981; Bowyer 1987; Gese et al. 1988). They can persist in diverse habitats from dry tropical deciduous forests in Mexico (Hildalgo-Mihart et al. 2006), through the prairies and savannahs of the Midwest (Kamler et al. 2003), to forests (Major and Sherburne 1987) into high mountains and high latitudes (Roy and Dorrance 1985), and both rural and urban areas (Person and Hirth 1991; Grinder and Krausman 2001). Coyotes can remain at sites for long periods and defend a territory, or can survive as nonterritorial transients, and move through areas more widely (Kamler and Gibson 2000). A key reason coyotes can achieve this is their dietary flexibility.

Coyotes have a broad diet consisting of foods as diverse as fruit (Andelt et al. 1987), crops (Kamler et al. 2007), invertebrates (Kamler et al. 2002; Kuiken et al. 2003; Way 2008), herpetofauna (Hernandez et al. 1994), birds (Witmer et al. 1995), and mammals ranging from small rodents (Morey et al. 2007) up to the size of adult Moose (*Alces alces*; Benson and Patterson 2013), but they do prioritize wild mammals (Jensen et al. 2022). Coyotes tend to be more carnivorous in temperate forests, and when in parts of their range that are sympatric with wolves (*C. lupus*; Jensen et al. 2022). Small mammals are important prey items throughout the year, but lagomorph consumption increases in winter and spring (Jensen et al. 2022). Coyotes and their prey adjust their strategies based on anthropogenic, environmental, and behavioral constraints, which can create pockets of refugia for prey and subordinate competitors, and ultimately result in partial separation of predators and prey across the landscape (Arias-Del Razo et al. 2012; Moll et al. 2018).

While extremely valuable, dietary summaries do not reveal the full story about ecology of an animal (Hayward et al. 2018). Food items that dominate the diet could do so simply because they are locally abundant, or they may be the result of active selection for them (Hayward and Kerley 2005). Hence, accounting for prey abundance is crucial to the interpretation of animal diets. If a species is consumed more frequently than expected based on its availability, it can be assumed that it is selected for, but if it is consumed less often than expected based on availability, it can be interpreted that the species is avoided (Hayward and Kerley 2005). Determining the selected prey of carnivores is important, because one of the main ecological drivers of carnivore densities across their range is the density of preferred prey (Miller et al. 2014). As such, the carrying capacity of carnivores can be better determined using the biomass of preferred prey, compared to total prey biomass (Hayward et al. 2007b). Here, we aimed to determine the selected prey of coyotes throughout their distribution, and to understand factors that affect this selection. We also compared the method we have used historically with a more modern iterative preference averaging (IPA) method (Nams and Hayward 2022). Finally, we hypothesized that reliance on scat and stomach content analyses among studies of coyote diets may bias prey selection estimates. In pursuit of that hypothesis, we tested whether prey selection varied with the frequency of occurrence of food items in scats compared to estimates of the number of prey individuals consumed to produce those scats.

## Materials and Methods

We obtained data on Coyote diet prior to December 2020 by searching Google Scholar, Web of Science, and gray literature such as dissertations and reports using keyword searches for diet* OR *predation* OR *food* AND *coyote* OR *Canis* AND *latrans*. Many studies had useful details on coyote diet but were excluded from the analysis due to insufficient information on prey densities (i.e., not recorded at all or recorded for <3 prey species), sample sizes <20, or an inability to locate these data from other sources. We used frequency of occurrence of food items in scats to document coyote diet.

### Selectivity indices.

There are many indices describing prey selectivity; however, all exhibit some degree of bias or increasing error with small sample sizes (Chesson 1978). We used Jacobs’ (1974) index $\left(D=\;\frac{\left(r_{i}-p_{i}\right)}{\left(r_{i}+p_{i}-2r_{i}p_{i}\right)}\right)$, where *r*_*i*_ is the proportion of food item *i* in the diet and *p*_*i*_ is the proportion of that food item present in the prey community (i.e., relative abundance), because it minimizes these biases and relates actual or relative prey abundance to actual or relative diet. Jacobs’ index ranges from +1 to −1, where +1 shows maximum selection and −1 shows maximum avoidance. We calculated relative frequency of occurrence as the measure of diet because we were focusing on the numerical selection of coyotes for prey species. The mean Jacobs’ index (*D*) for each prey species was calculated from all the sites and time periods available in our review, and these values were tested for significant selection or avoidance using *t*-tests against the mean of 0. This type of analysis is not biased by results from one particular area because, for a species to be significantly selected for or avoided, several studies must have produced similar results (Lyngdoh et al. 2014). We transformed the Jacobs’ index values with Fisher’s *Z-*transformation, so it was no longer bound between +1 and −1 and to facilitate testing of the drivers of coyote prey selection using linear models.

We also used Jacobs’ index, with the IPA method, that estimates prey consumption by taking into account missing data to derive estimates for the full suite of species within a prey community (Nams and Hayward 2022). Finally, several studies have reported on the defecation rates of wild canids with similar digestive systems (one study on coyotes and four on wolves) when fed specific food items, where one larger individual is likely to be represented in more coyote scats than small species (Floyd et al. 1978; Weaver and Hoffman 1979; Weaver 1993; Ruehe et al. 2003; Jethva and Jhala 2004). We determined the relationship between the number of scats produced by coyotes for prey of different body mass (biomass method) that enabled us to estimate dietary selection based on the likely number of prey individuals of each species consumed. We compared the three selection estimates (Jacobs’ index based on frequency of occurrence, Jacobs’ index based on IPA to account for missing species, and Jacobs’ index based on the biomass of the prey) using an ANOVA because they were normally distributed.

### Prey species characteristics.

We analyzed the drivers of coyote diet selection using general linear models with Gaussian distributions. Coyotes are generally believed to eat small- to medium-sized prey (Nowak 1999), so we used ¾ of mean adult female body mass to account for juveniles and subadults consumed in accordance with previous studies (e.g., Jooste et al. 2013; Table 1). Body mass data for each species were derived from public databases for amphibians (Oliveira et al. 2017), birds (Wilman et al. 2014), and mammals (Faurby et al. 2018). Body mass was estimated from the allometric relationship between mass and snout–vent length for lizards (Meiri 2010), and via total length for snakes (Pough 1980; Feldman and Meiri 2013). We log-transformed the body mass covariate to satisfy model assumptions and facilitate model selection.

**Table 1.** Summary results of coyote prey selection including prey species, preference/avoidance, Jacobs’ index, sample size, body mass, dietary records, and summary statistics. The symbol + indicates significant preference, - significant avoidance, and ~ no preference.

Species	Scientific name	Pref/avoid	Jacobs index	*n*	Body mass (kg)	Kills	*t*	d.f.	*P*
Beaver, American	*Castor canadensis*	*~*	*−*0.77 ± 0.14	2	21.82	0.03 ± 0.04	*−*5.58	1	0.110
Birds	Aves	*~*	*−*0.09 ± 0.20	6	0.2	0.05 ± 0.1	−0.45	5	0.670
Chipmunk, least	*Neotamias minimus*	−	−0.75 ± 0.06	7	0.14	0.02 ± 0.01	−12.72	6	0.000
Chipmunks	Sciuridae	*~*	−0.49 ± 0.18	7	0.11	0.01 ± 0.01	−2.79	6	0.030
Coyote	*Canis latrans*	−	−0.74 ± 0.04	5	13.41	0.02 ± 0.01	−17.9	4	0.000
Deer, mule	*Odocoileus hemionus*	*~*	0.14 ± 0.20	18	54.21	0.08 ± 0.03	0.7	17	0.490
Deer, white-tailed	*Odocoileus virginianus*	*+*	0.47 ± 0.10	38	55.51	0.16 ± 0.03	4.71	37	0.000
Elk	*Cervus canadensis*	*~*	0.23 ± 0.39	2	131.25	0.16 ± 0.12	0.58	1	0.660
Gopher, northern pocket	*Thomomys talpoides*	−	−0.56 ± 0.15	8	0.13	0.11 ± 0.04	−3.86	7	0.010
Grasshopper	Caelifera	*~*	−0.05 ± 0.55	3	0.01	0.06 ± 0.05	−0.09	2	0.930
Grouse, spruce	*Canachites canadensis*	−	−0.85 ± 0.06	5	0.47	0.03 ± 0.01	−13.85	4	0.000
Hare, snowshoe	*Lepus americanus*	*~*	0.2 ± 0.13	30	1.71	0.2 ± 0.04	1.52	29	0.140
Invertebrates	Invertebrata	*~* −	−0.71 ± 0.22	4	0	0.07 ± 0.05	−3.22	3	0.050
Jackrabbit	*Lepus spp.*	*+*	0.96 ± 0.01	4	2.45	0.1 ± 0.08	75.76	3	0.000
Jackrabbit, black-tailed	*Lepus californicus*	*~*	0.2 ± 0.11	29	2.42	0.15 ± 0.04	1.84	28	0.080
Lemming, southern bog	*Synaptomys cooperi*	*~*	0.62 ± 0.20	2	0.03	0.03 ± 0.08	3.12	1	0.200
Marmot, yellow-bellied	*Marmota flaviventris*	*~*	−0.43 ± 0.29	2	3.35	0.03 ± 0.02	−1.47	1	0.380
Moose	*Alces alces*	*~*	0.04 ± 0.20	7	357	0.11 ± 0.06	0.19	6	0.860
Mouse, California deer	*Peromyscus californicus*	*−*	−0.91 ± 0.09	4	0.02	0 ± 0	−9.92	3	0.000
Mouse, deer	*Peromyscus spp.*	*−*	−0.72 ± 0.06	64	0.02	0.03 ± 0.01	−11.86	63	0.000
Mouse, fulvous harvest	*Reithrodontomys fulvescens*	*~*	−0.39 ± 0.58	2	0.01	0.01 ± 0.01	−0.67	1	0.620
Mouse, Great Basin pocket	*Perognathus parvus*	*~*	−0.1 ± 0.20	12	0.02	0.03 ± 0.01	−0.5	11	0.630
Mouse, harvest	*Reithrodontomys spp.*	*−*	−0.83 ± 0.05	8	0.01	0.03 ± 0.02	−18.42	7	0.000
Mouse, hispid pocket	*Chaetodipus hispidus*	*~*	−0.37 ± 0.30	6	0.03	0.01 ± 0	−1.24	5	0.270
Mouse, northern grasshopper	*Onychomys leucogaster*	*~*	−0.63 ± 0.27	4	0.03	0.01 ± 0.01	−2.32	3	0.100
Mouse, pinyon deer	*Peromyscus truei*	*−*	−0.66 ± 0.18	4	0.03	0.01 ± 0	−3.66	3	0.040
Mouse, pocket	*Chaetodipus spp.*	*~*	−0.24 ± 0.16	10	0.03	0.04 ± 0.02	−1.52	9	0.160
Mouse, southern marsh harvest	*Reithrodontomys megalotis*	−	−0.79 ± 0.21	4	0.01	0.01 ± 0.01	−3.7	3	0.030
Mouse, western harvest	*Reithrodontomys megalotis*	*~*	0.14 ± 0.20	10	0.01	0.03 ± 0.02	0.72	9	0.490
Mouse, western jumping	*Zapus princeps*	*~*	−0.65 ± 0.35	2	0.02	0.03 ± 0.03	−1.85	1	0.320
Mouse, white-footed	*Peromyscus leucopus*	*−*	−0.56 ± 0.17	6	0.02	0.05 ± 0.02	−3.33	5	0.020
Muskrat	*Ondatra zibethicus*	*−*	−0.83 ± 0.08	8	1.07	0.02 ± 0.02	−9.86	7	0.000
Opossum, Virginia	*Didelphis virginiana*	*~ −*	−0.92 ± 0.08	2	2.2	0.01 ± 0.01	−12.19	1	0.050
Pheasant, ring-necked	*Phasianus colchicus*	*~*	0.34 ± 0.45	4	1.12	0.02 ± 0.02	0.75	3	0.510
Pronghorn	*Antilocapra americana*	*~*	−0.12 ± 0.35	4	46.08	0.03 ± 0.03	−0.34	3	0.760
Rabbit, desert cottontail	*Sylvilagus audubonii*	*~ +*	0.35 ± 0.16	14	0.89	0.08 ± 0.02	2.11	13	0.050
Rabbit, eastern cottontail	*Sylvilagus floridanus*	*+*	0.36 ± 0.09	15	1.17	0.13 ± 0.03	4.21	14	0.000
Rabbit, mountain cottontail	*Sylvilagus nuttallii*	*~ +*	0.67 ± 0.21	3	0.76	0.06 ± 0.04	3.23	2	0.080
Raccoon, northern	*Procyon lotor*	*−*	−0.94 ± 0.02	2	6.55	0.02 ± 0.04	−56.8	1	0.010
Rat, hispid cotton	*Sigmodon hispidus*	*~*	0.02 ± 0.10	27	0.09	0.81 ± 1.80	0.23	26	0.820
Rat, Ord’s kangaroo	*Dipodomys ordii*	*−*	−0.48 ± 0.10	11	0.06	0.04 ± 0.01	−4.67	10	0.000
Sheep	*Ovis aries*	*~*	−0.02 ± 0.13	6	70	0.04 ± 0.04	−0.13	5	0.900
Sheep, Dall	*Ovis dalli*	*+*	0.67 ± 0	3	55.65	0.06 ± 0.01	229.15	2	0.000
Skunk, striped	*Mephitis mephitis*	*~*	−0.41 ± 0.59	2	2.09	0.02 ± 0.03	−0.7	1	0.610
Small mammals	*Mammalia*	*−*	−0.55 ± 0.15	15	0.03	0.43 ± 0.9	−3.64	14	0.000
Squirrel, American red	*Tamiasciurus hudsonicus*	*−*	−0.82 ± 0.04	8	0.2	0.02 ± 0.01	−18.9	7	0.000
Squirrel, fox	*Sciurus niger*	*−*	−0.98 ± 0.02	2	0.7	0 ± 0	−42.69	1	0.010
Squirrel, golden-mantled ground	*Callospermophilus lateralis*	*~*	0.04 ± 0.29	3	0.19	0.03 ± 0.03	0.15	2	0.900
Squirrel, ground	Sciuridae	*~*	−0.48 ± 0.31	4	0.27	0.06 ± 0.07	−1.57	3	0.220
Squirrel, Townsend’s ground	*Urocitellus townsendii*	*~*	−0.24 ± 0.14	3	0.41	0.04 ± 0.02	−1.76	2	0.220
Squirrel, Uinta ground	*Urocitellus armatus*	*~*	0.12 ± 0.50	4	0.31	0.06 ± 0.03	0.25	3	0.820
Vole, California	*Microtus californicus*	*+*	0.25 ± 0.05	4	0.06	0.08 ± 0.05	5.38	3	0.010
Vole, meadow	*Microtus pennsylvanicus*	*−*	−0.82 ± 0.13	3	0.04	0.08 ± 0.05	−6.3	2	0.020
Vole, prairie	*Microtus ochrogaster*	*~*	−0.13 ± 0.18	11	0.04	0.1 ± 0.02	−0.74	10	0.470
Vole, southern red-backed	*Myodes gapperi*	−	−0.87 ± 0.10	6	0.02	0.03 ± 0.02	−8.43	5	0.000
Waterfowl	Anatidae	−	−0.97 ± 0.02	3	1	0.04 ± 0.04	−58.55	2	0.000
Woodchuck/groundhog	*Marmota monax*	*~*	−0.61 ± 0.16	2	3.81	0.05 ± 0.05	−3.84	1	0.160
Woodrat, desert	*Neotoma lepida*	*~*	−0.52 ± 0.38	2	0.16	0.05 ± 0.06	−1.35	1	0.410
Woodrat, eastern	*Neotoma floridana*	*~*	0.47 ± 0.05	2	0.24	0.02 ± 0.01	9.98	1	0.060
Woodrat, southern plains	*Neotoma micropus*	*~*	−0.07 ± 0.08	27	0.24	0.06 ± 0.01	−0.86	26	0.400

**Fig. 1.**
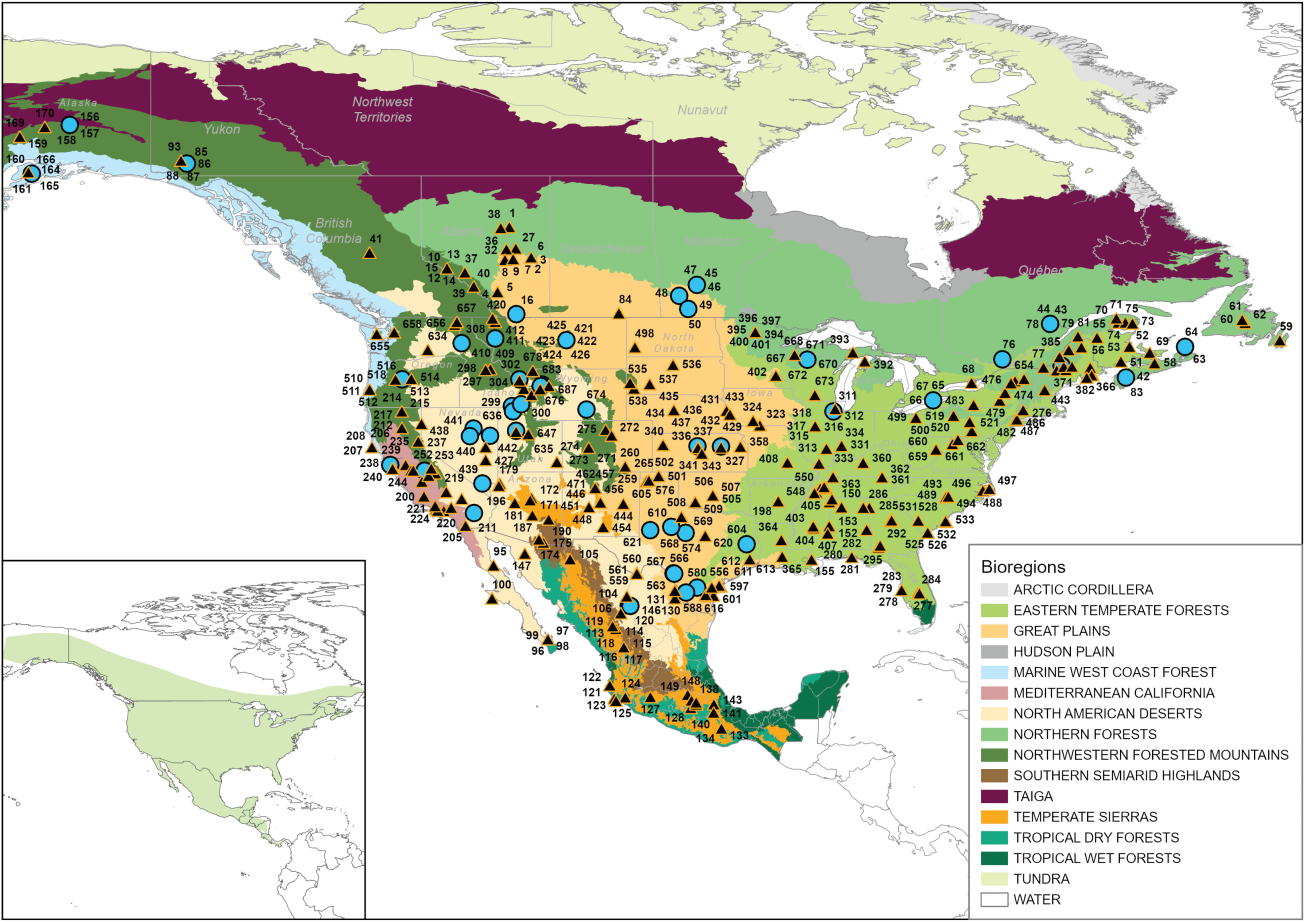
Map of the distribution of coyotes (shading in insert) and the location of studies assessed in this study stratified by bioregion. Coyote distribution is shown in the shaded area in the inset figure from the IUCN Red List. Site details are presented in Supplemental Data SD1.

The social organization of prey species, their habitat use, and their threat to predators can also affect the ability of a predator to capture the prey and ability of prey to detect predators (Hayward and Kerley 2005). Group size categories of prey were classified as 1 = solitary; 2 = pairs; 3 = small groups of 3–10; 4 = larger groups of 11–25; and 5 = very large groups >25 following previous studies (Table 1; Hayward et al. 2016, 2017). We modeled group size as a continuous variable in recognition that these categories grade upwards from solitary individuals. We subjectively estimated the likely threat of each prey species based on their possession of weaponry (antlers, horns, or toxins), aggressive nature, and body size—where 0 = no likelihood of injury; 1 = potential for injury; 2 = potential for death following our previous studies (Hayward et al. 2006b), and again this variable was modeled as continuous. Ecoregion types (Fig. 1), habitat (rural, suburban, urban, and wilderness), and the mean annual temperature and precipitation for each site were collated from Wiken et al. (2011). We log-transformed rainfall and body mass, and standardized rainfall and temperature using a *z*-score transformation to facilitate model selection.

It is difficult to distinguish kills from scavenging in scat and stomach content analyses, and how the item came to be eaten is rarely observed (Reynolds and Aebischer 1991). To address this constraint, we included the presence or absence of wolves and pumas (*Puma concolor*) as apex carnivores as covariates in the models, because these species may provide coyotes with large amounts of carrion in places where they are sympatric. We acknowledge that this ignores the carcasses available for coyotes to scavenge from human hunters, road kills, or natural causes (Wilmers et al. 2003b), but expect these to be more evenly dispersed across the range of coyotes than puma or wolf presence, although seasonal peaks in hunting sourced carcasses are likely. The “coywolf,” or eastern coyote, is considered a hybrid between coyotes and wolves (Kays et al. 2010; Thornton and Murray 2014), and they are larger in body mass than pure coyotes (Way 2013), so we hypothesized that this may affect diet selection and overall niche. Hence, we included whether the study occurred within coywolf distribution limits as a covariate in our models, based on eastern coyote distributional maps (Kays et al. 2010; Way 2013). We did not model Red Wolf (*C. rufus*) impacts on coyote prey selection because the former occurs at such low densities over such a small distribution that we were unlikely to identify any patterns. We hypothesized that the presence of deer may also influence prey selection as they may be selected for, so included deer presence at a site in our models. We included coyote density and home range size in our model to determine if these factors affected prey selection, and we obtained these data directly from the studies cited (Supplementary Data SD1). We also hypothesized that prey selection may change throughout the year, and thus fit a seasonal (Period = annual, spring, summer, fall, winter) dimension into our models. The studies we found relied on either DNA, scat, or stomach content analyses. Differential rates of digestion through the gut could lead to biases, so we tested whether there was a difference in selection for each species based on the method of data collection using a two-factor ANOVA.

### Models of prey selection.

In total, we considered 13 covariates and tested their influence on coyote prey selection using general linear models with Gaussian distributions on the Jacobs’ index values calculated from the frequency of occurrence data to enable comparisons with previous studies (Table 1). We ran partial correlations on covariates in the general linear model using the *ppcor* package (Kim 2015) and found no correlations. We evaluated all possible combinations of models derived from the covariates and conducted model selection via Akaike’s information criterion (AIC) within a maximum likelihood framework (Akaike 1973, 1974) using the *MuMIn* and *AICcmodavg* packages (Barton 2013; Mazerolle 2020). We used the sum of Akaike’s weights (*w*_*i*_) to determine the relative importance of each covariate (Burnham and Anderson 1998). We also present model-averaged parameter estimates using the full suite of models. Strongly supported relationships among individual variables were plotted using linear or loess best-fit models.

We determined the *accessible* prey weight range of coyotes using break point analysis with segmented models following Clements et al. (2014). We ran a Kruskal–Wallis test to compare the mean Jacobs’ index values of each group between the breakpoints of the segmented model. We calculated the *ideal* prey mass as the mean body mass of those species that were significantly selected for. We estimated the predator-to-prey mass ratio of coyotes using the mean body mass estimates of coyotes recorded in the studies we used, and their ideal prey mass. All analyses were conducted in R (R Core Development Team 2008).

## Results

### Number and distribution of studies.

We reviewed 445 studies on coyote diet and were able to use data from 283 studies from 691 separate time periods and/or places over a period of 684 cumulative study years with an average of one year per time and/or place (Fig. 1, Supplementary Data SD1). These reported a total of 216,353 dietary records from 121,789 scats, 91,598 stomach contents, and 103 DNA records (remainder were a mix of stomach and scats; Table 1) of 772 prey species, from which Jacobs’ index values could be estimated for 87 species. There were 93 studies from Canada (from eight provinces), 57 from Mexico (14 states), and 539 from the United States (40 states; Fig. 1). The majority of these studies were from rural areas (530), with 87 from wilderness areas, 19 from suburban areas, and 12 from urban areas (the remainder were from a range of sites, or were not specific enough in their location to define). Forty-eight of these studies reporting on 125 sites or times yielded dietary and prey abundance data, which enabled us to derive Jacobs’ index values of selection of 60 taxa based on 25,721 dietary records (Fig. 1). Twenty-five of these 125 data sets came from Canada, seven from Mexico, and 93 from the United States.

### Prey selection indices.

**Fig. 2.**
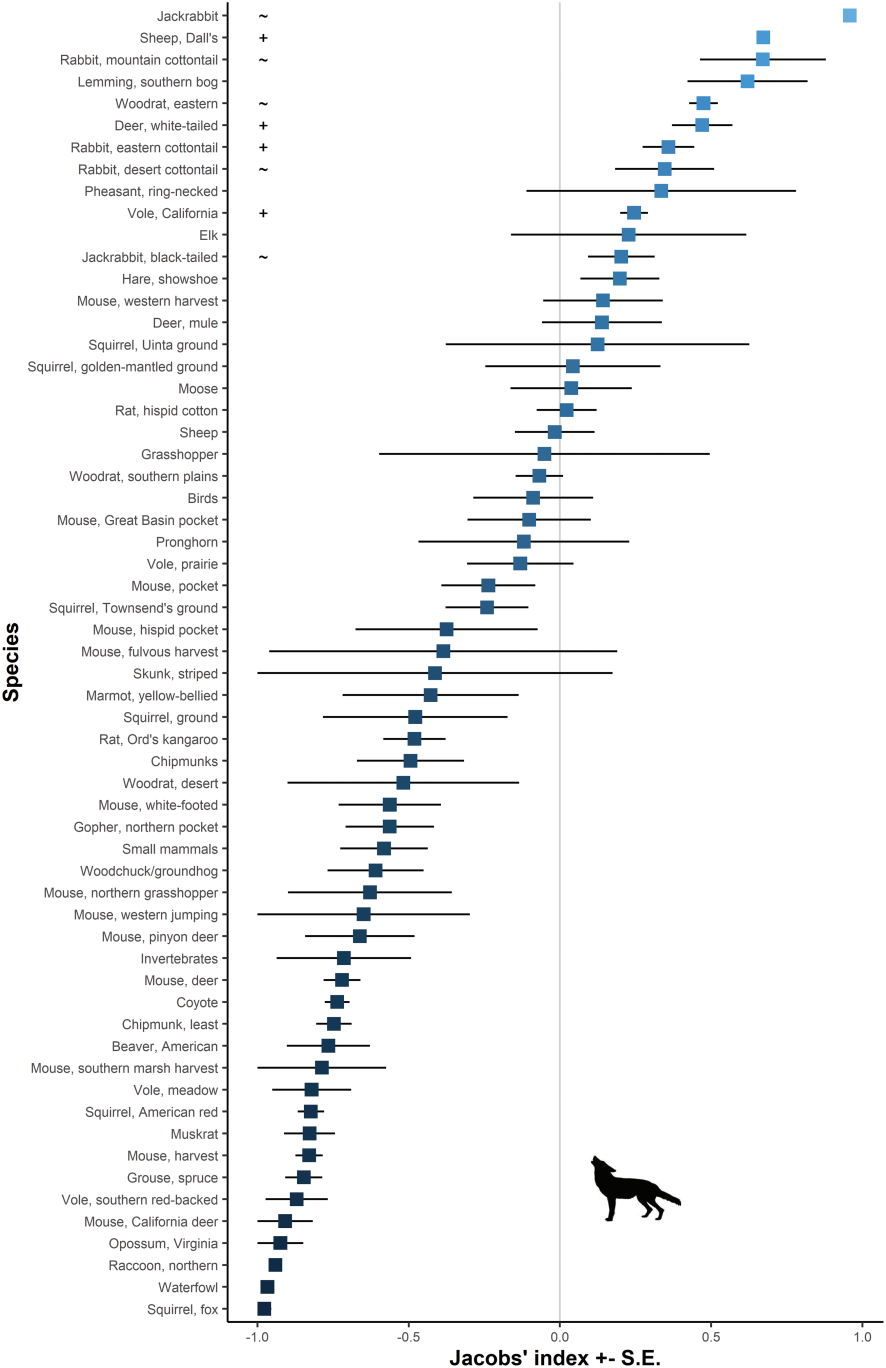
Coyote prey preferences with mean value shading scaled by the degree of preference for each prey species. Preferred prey are to the right of the grey line of no selection at D = 0, and significantly preferred prey are denoted with a ‘+’ and those likely to become significantly preferred with a larger size with ‘~’.

The three methods of determining selection (traditional, IPA on scats, and IPA on biomass) yielded significantly different results (*F*(species)_17, 951_ = 28.87, *P* < 0.001; *F*(method)_2, 951_ = 84.30, *P* < 0.001; *F*(interaction)_34, 951_ = 8.52, *P* < 0.001). Nonetheless, there was a significant correlation between methods (traditional vs. IPA scats *R* = 0.76, *P* < 0.001; traditional vs. IPA biomass *R* = 0.45, *P* < 0.001; IPA scats vs. biomass *R* = 0.36, *P* < 0.001; d.f. = 333 for all). Larger species were least selected for based on the biomass method (Fig. 2).

### Prey selection.

Mammals were the most frequently reported broad taxonomic group (7,199 dietary records), with vegetation found in 2,664 records, birds in 1,511, invertebrates in 1,257, and herpetofauna in 499 (Table 1). Rodents were the most commonly consumed Order of mammals (3,498 records), followed by lagomorphs (950; Table 1). White-tailed deer were the most frequently recorded species in coyote diet (317 dietary records), followed by snowshoe hares (175), hispid cotton rats (*Sigmodon hispidus*) (169), and mule deer (147; Table 1).

Mammals were the most abundant prey at the study sites, with rodents (notably mice), lagomorphs, and ungulates being most common (Table 1). White-tailed deer were the most commonly recorded potential prey (comprising 8.0 ± 0.03% of the available prey community where they occur), followed by snowshoe hares (31.1 ± 6.1%), black-tailed jackrabbits (26.8 ± 6.7%), and hispid cotton rats (5.1 ± 1.0%; Table 1).

**Fig. 3.**
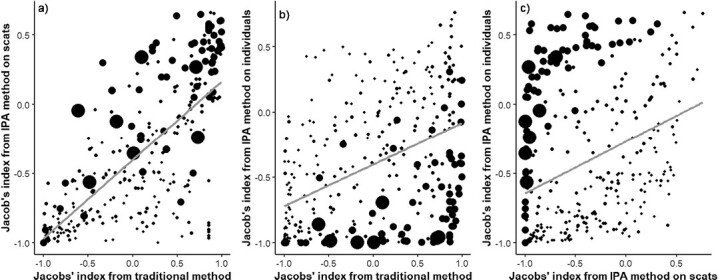
Relationships between the three methods of deriving Jacobs’ index estimates for: (a) the dominant prey species (those with *n* > 5); (b) traditional method used in previous studies of predator prey preferences (R = 0.76, *P* < 0.001); and (c) the iterative preference averaging (IPAScat) based on the frequency of occurrence of a prey item (as in the traditional method; R = 0.45, *P* < 0.001) and the estimated number of individuals the scats were derived from based on defecation rates (R = 0.36, *P* < 0.001). Point size is scaled by prey body mass.

Using the traditional method, Dall’s sheep (Jacobs’ index *D* = 0.67 ± 0.02), white-tailed deer (0.47 ± 0.10), eastern cottontail rabbits (0.36 ± 0.09), and California voles were significantly selected for (*t*_Dall’s sheep_ = 229.2, d.f. = 2, *P* < 0.001; *t*_white-tailed deer_ = 4.71, d.f. = 37, *P* < 0.001; *t*_eastern cottontail_ = 4.21, d.f. = 14, *P* = 0.001; *t*_California vole_ = 5.38, d.f. = 3, *P* = 0.013; Fig. 3). A larger sample size may also see black-tailed jackrabbit (0.20 ± 0.10; *t* = 1.84, d.f. = 28, *P* = 0.080), mountain cottontail rabbits (*S. nuttalli*) (0.67 ± 0.21; *t* = 3.23, d.f. = 2, *P* = 0.084), eastern woodrats (*Neotoma floridana*) (0.47 ± 0.10; *t* = 9.98, d.f. = 1, *P* = 0.064), and desert cottontail rabbits (*S. audubonii*) (0.35 ± 0.16; *t* = 2.11, d.f. = 13, *P* = 0.055) selected for by the coyote (Fig. 3). Jackrabbits, as a broader taxonomic unit, were also selected for (0.96 ± 0.1; *t* = 75.76, d.f. = 3, *P* < 0.001). Fox squirrels (*Sciurus niger*), northern raccoons (*Procyon lotor*), southern red-backed voles (*Myodes gapperi*), spruce grouse (*Canachites canadensis*), harvest mice (*Reithrodontomys* spp.), muskrat (*Ondatra zibethicus*), American red squirrels (*Tamiasciurus hudsonicus*), meadow voles (*Microtus* spp.), least chipmunks (*Neotamias minimus*), pinyon deer mice (*Peromyscus truei*), northern pocket gophers (*Thomomys talpoides*), white-footed mice (*P. leucopus*), chipmunks (*Tamias* spp., *Neotamias* spp.), and Ord’s kangaroo rats (*Dipodomys ordii*) were all significantly avoided (Fig. 3; Table 1). The remaining species, including livestock, were consumed in accordance with their relative abundance within the prey community.

There was a significant relationship between the body mass of a prey item and the number of scats produced by large canids (log_10_ Number of scats produced = 1.32 × log_10_ Carcass mass (kg) − 0.89: *r*^2^ = 0.932, *n* = 58, *P* < 0.001). The largest prey items yielded > 200 scats, while 10 individuals of the smallest prey species may be necessary before showing up in one scat.

The mean body mass of coyotes reported in the studies we used was 13.2 kg. This yields a predator-to-preferred prey weight ratio of 1:2.13 based on the traditional method of selection, identifying coyotes selection for consuming prey over twice as large as themselves on average.

**Fig. 4.**
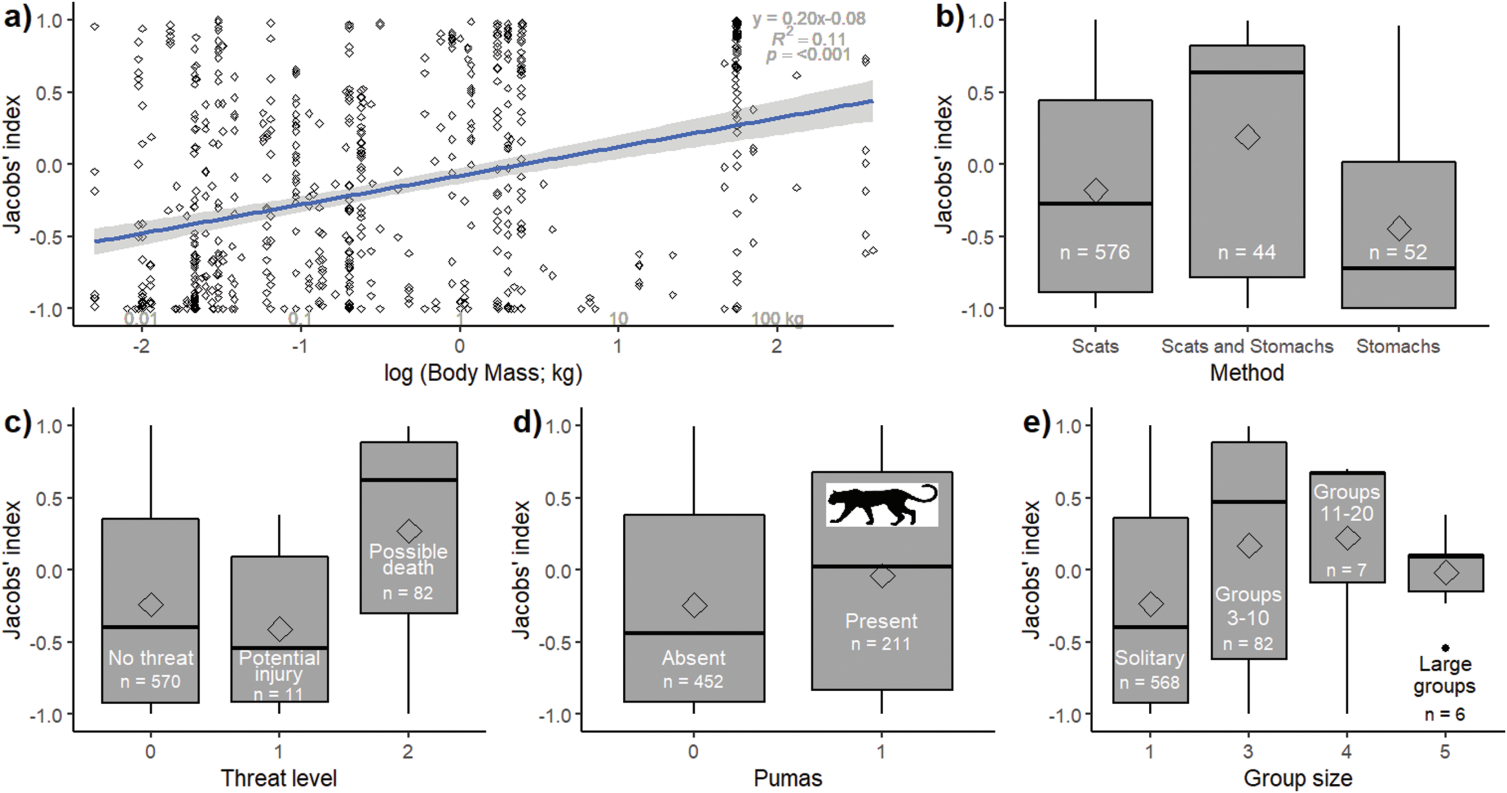
Drivers of coyote prey preferences based on the top four most influential variables from the linear modeling. These plots show Jacobs’ index related to: (a) body mass on the log_10_ scale (∑*w*_*i*_ = 1); (b) the method that coyote diet was ascertained (∑*w*_*i*_ = 1); (c) the level of threat posed by prey species (∑*w*_*i*_ = 0.92); (d) the presence and absence of pumas (∑*w*_*i*_ = 0.76); and (e) the size of groups these prey species occurred in (∑*w*_*i*_ = 0.74). The sample size (*n*) is shown on the boxplots.

The most supported linear models of the traditional prey selection estimates exhibited vastly improved model fit compared to the null model (ΔAIC_c_ = 85.2; Table 1). Prey body mass and the method of data collection were the most important drivers of coyote prey selection (∑*w*_*i*_ = 1 for both; Table 1). Coyotes increasingly selected larger prey species (Fig. 4a), and studies that used scats and stomachs yielded more selection than those that used each individually (Fig. 4b). The degree of threat a prey species posed was also influential (∑*w*_*i*_ = 0.90), along with the presence of pumas (0.76) and prey group size (∑*w*_*i*_ = 0.74; Table 1). Coyotes consumed species that were riskier to hunt (Fig. 4c) in the presence of pumas (Fig. 4d), and were least likely to hunt solitary species (Fig. 4e).

**Fig. 5.**
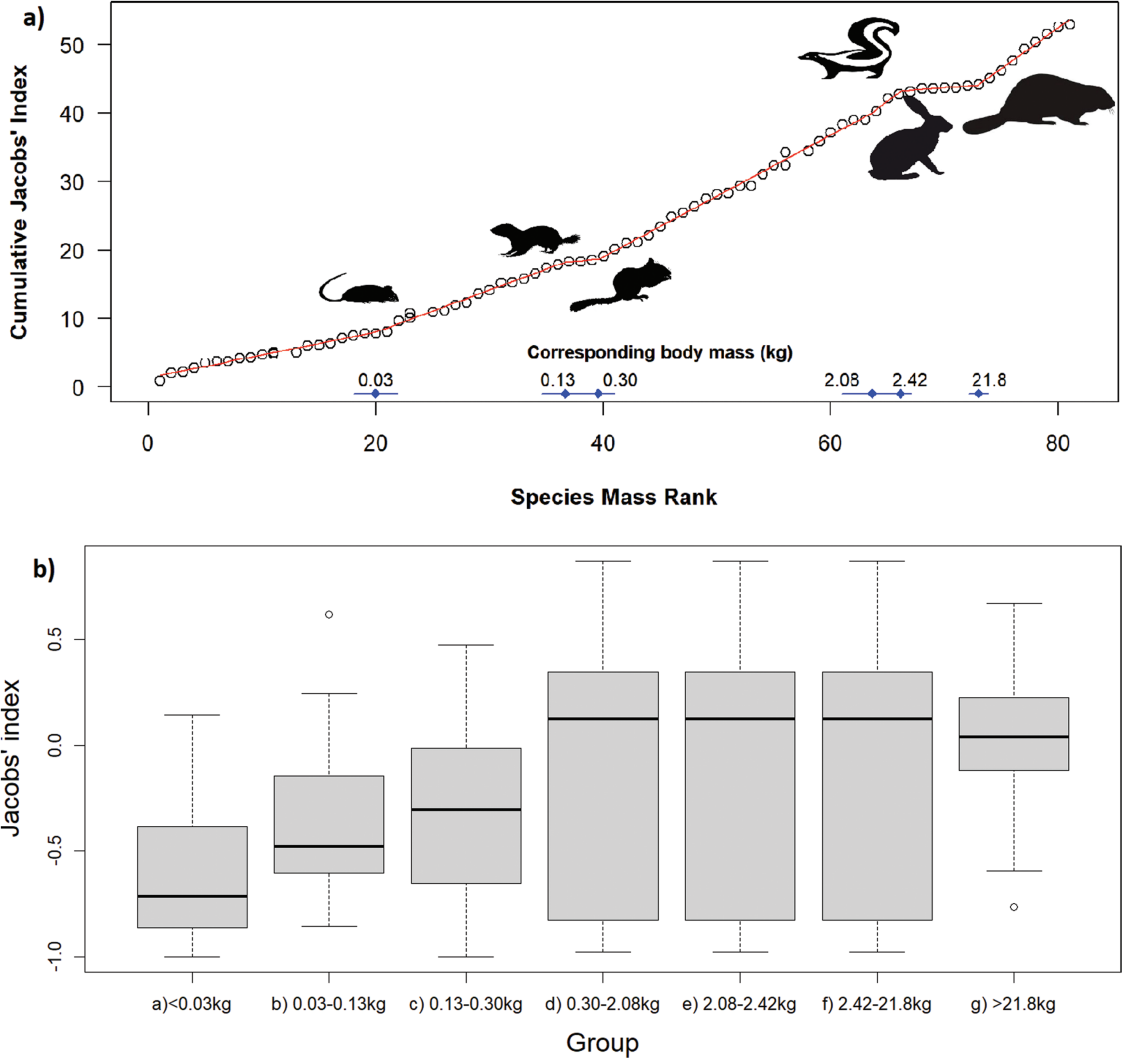
a) Segmented model results for coyote prey preferences with 6 breakpoints as the most supported model. The largest prey are significantly more preferred than the smallest group (<0.03 kg; Kruskal-Wallis = 12.209, d.f. = 4, *P* = 0.016). b) Box plot of Jacobs’ index values for each prey mass group identified in the segmented model.

Coyote selection for individual prey species varied across their range. Mule deer (*r*^2^ = 0.63, *n* = 18, *P* < 0.001) and snowshoe hares (*r*^2^ = 0.41, *n* = 30, *P* = 0.001) were significantly more selected for with increasing latitude, whereas black-tailed jackrabbit (*r*^2^ = 0.18, *n* = 29, *P* = 0.010) was increasingly selected for with decreasing latitude (Fig. 5). This relationship was not driven by rainfall (Table 1).

**Fig. 6.**
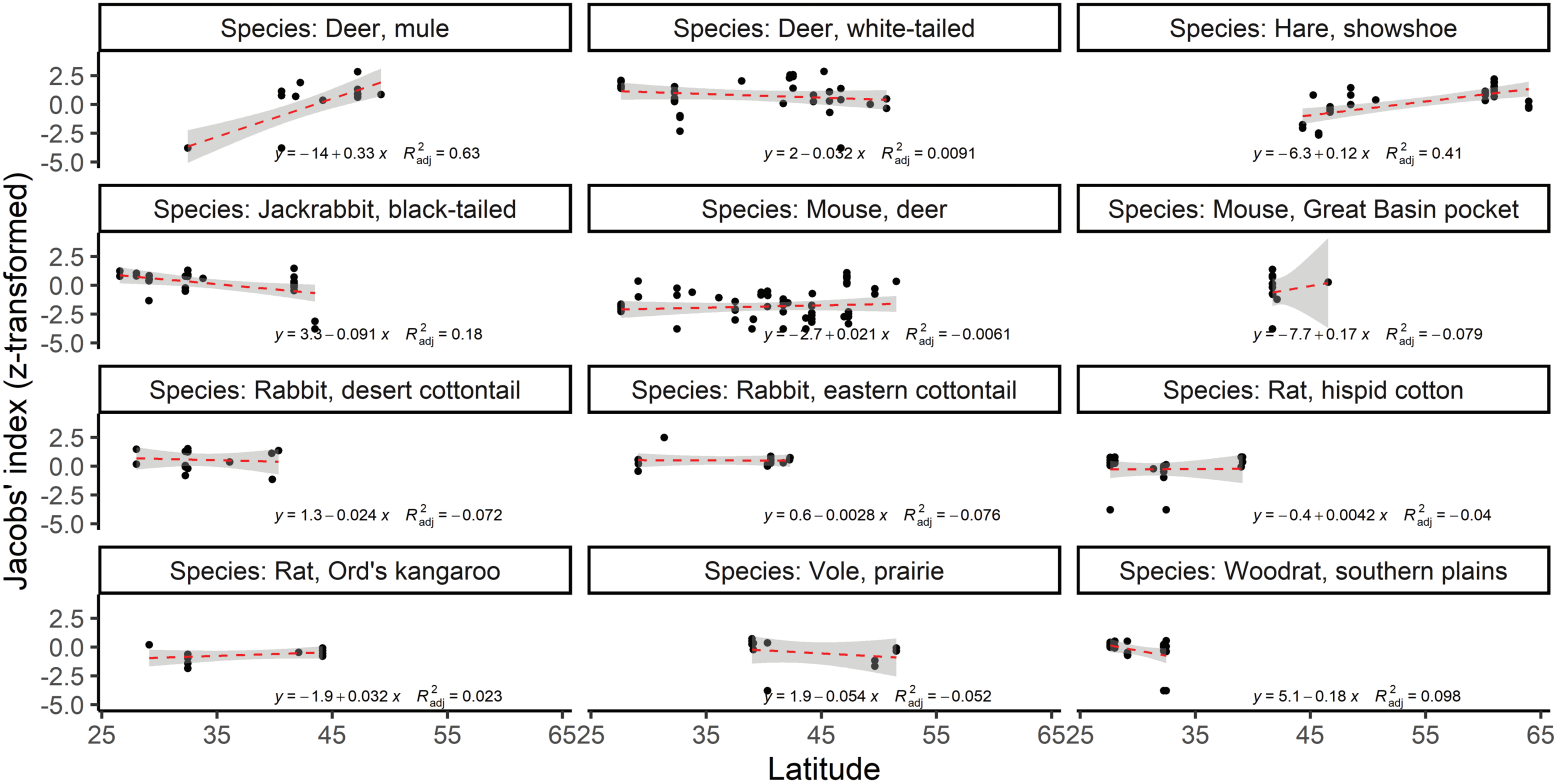
Relationship between the degree of preference coyotes exhibit to the most preyed upon species and latitude, using Fisher’s z-transformation.

While we detected no significant difference between coyote prey selection throughout the year (*F*_4, 241_ = 1.443, *P* = 0.220), coyote selection for individual prey species obviously varied by species for the 12 most commonly consumed prey (*F*_11, 241_ = 12.633, *P* < 0.001), but there was also an interaction showing specific selection varies seasonally (*F*_39, 241_ = 1.695, *P* = 0.009). Great Basin Pocket Mouse (*Perognathus mollipilosus*), Prairie Vole (*M. ochrogaster*), and Ord’s Kangaroo Rat disappeared from coyote diet altogether in the cooler months (black-tailed jackrabbit become highly avoided at this time), while mule deer were most selected for in these cooler months (Fig. 6).

**Fig. 7.**
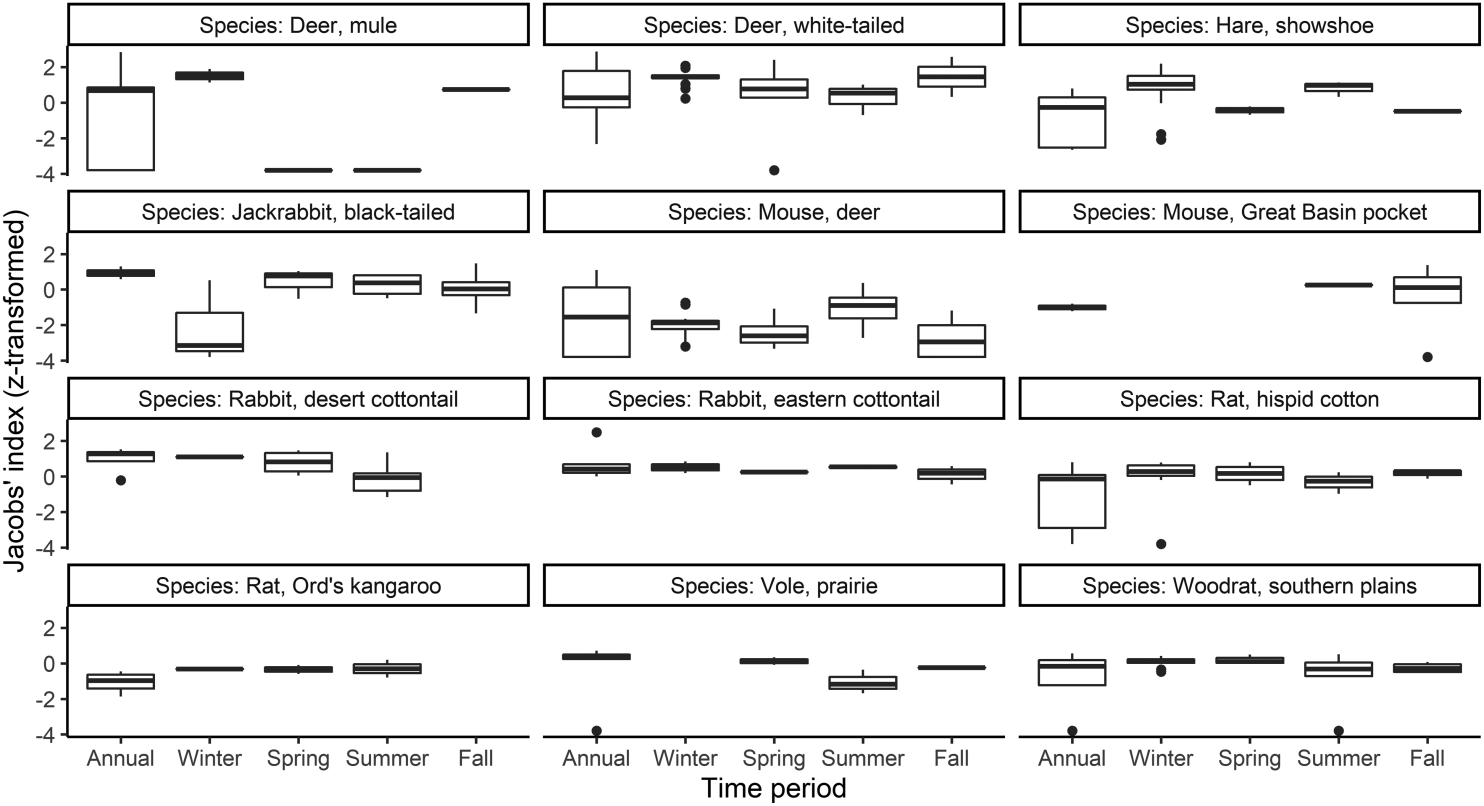
Differences in coyote prey preferences for the most preyed upon species stratified by the time period.

Coyote population density was related to the degree of selection for two prey species. Mule deer were increasingly selected for at higher coyote densities, while black-tailed jackrabbit were increasingly avoided with higher coyote densities (Fig. 7). There was no such relationship for white-tailed deer, deer mouse, hispid cotton rat, or Southern Plains Woodrat (*N. micropus*; Fig. 7). There was also no relationship between prey selection for any species and coyote home range size. At 18 sites where prey abundance data were available, mule deer were only consumed when wolves were absent, and white-tailed deer were only found in the diet when wolves were present.

**Fig. 8.**
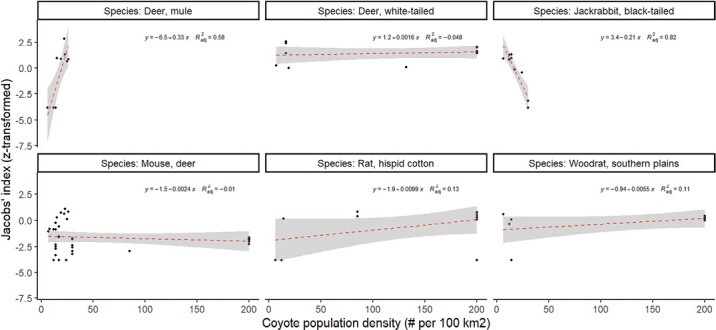
Relationship between the degree of preference exhibited by coyotes and Coyote population density for the six species with sufficient data using Fisher’s z-transformation.of the study using Fisher’s z-transformation.

The most supported segmented model of coyote prey selection had six breakpoints (AIC = 102.26), which had substantially more support than the next most supported model (three breakpoints; ΔAIC = 73.24). The breakpoints occurred at 0.03, 0.13, 0.30, 2.08, 2.42, and 21.8 kg (Fig. 8a). There was a significant difference in the selection coyotes exhibited to each breakpoint group (*F*_6, 89_ = 3.03, *P* = 0.009) with species weighing less than 0.03 kg significantly avoided compared to species exceeding 21.8 kg (Tukey’s *P* = 0.048). The preferred prey weight range of coyotes is 0.30–21.8 kg, and accessible prey body masses for coyotes are above 0.30 kg (~ chipmunks; Fig. 8b).

## Discussion

### Prey selection.

Coyotes exhibit dietary flexibility and this is reflected in their prey selection. On average, coyotes selected prey around twice their size, but consumed a diversity of prey species from ungulates exceeding 22 kg, down to rodents (Fig. 3). The extent of this preferred weight range likely reflects the behavioral plasticity of coyote hunting from individuals to pairs and packs (Bowen 1981; Bowyer 1987; Gese et al. 1988). Also, social status within coyote groups affects diets, as evidenced by large pups preying more on small rodents compared to adults (Gese et al. 1996), and only dominant adults killing sheep (*O. aries*; Blejwas et al. 2006). There is also evidence of resource partitioning among coyote group members, with juveniles (<18 months) feeding primarily on small rodents, and adults mainly selecting deer (Gese et al. 1996). Coyote selection for prey twice their size may be affected by eating the young of large species; however, the method of using ¾ adult female body mass does account for this to an extent. Dall’s sheep, white-tailed deer, eastern cottontail, and California vole were significantly selected prey and reflect the dietary flexibility of coyotes—so too does the segmented model results that show the most selected for body masses of coyote prey were above 300 g (Fig. 8). We hypothesize that larger prey species are selectively hunted by dominant individuals or coyotes in groups, whereas solitary foraging coyotes, such as juveniles or transients, select smaller species. We acknowledge that coyotes could scavenge the larger selected species (Thornton et al. 2004; Prugh 2005; Schrecengost et al. 2008), but note also that these species have been regularly documented as being killed by coyotes (e.g., Gese et al. 1996; Scotton 1998; Patterson and Messier 2000; Prugh 2004; Van de Kerk et al. 2020).

The selective predation on Dall’s sheep is interesting. This may be because Dall’s sheep are about the same body mass as mule and white-tailed deer, and the only meso-ungulate present in the habitats where it occurs. There is additional evidence that coyotes target Dall’s sheep because reducing coyote abundance in Alaska led to an increase in Dall’s sheep abundance (Mitchell et al. 2015, 2022). In addition, extreme northern regions have fewer prey species present, and many of these hibernate or live under deep snow and thus are only seasonally available, or exhibit population cycles when they may be extremely scarce for prolonged periods. These factors may explain coyote selection for Dall’s sheep.

Vegetation is clearly an important component of coyote diet (Jensen et al. 2022). No studies reported the abundance of vegetation, so we were unable to determine the selection for it, but given the widespread availability of vegetation, we doubt that it is actively selected for over meat.

The linear models support these findings with coyote selection for larger prey species (Fig. 4a) being detected irrespective of the degree of threat that the prey possess (Fig. 4c), although if they are selecting for more vulnerable individuals, such as the young of these species, this threat might be reduced. The selection for larger species may also reflect a tendency to scavenge from puma-provided carcasses. The chance of finding at least one vulnerable individual in a larger aggregation, which are easier to detect (Hamilton 1971), might also help explain coyote selection for hunting larger groups. Coyotes generally avoid solitary prey species (Fig. 4b), but we attribute this result to an avoidance of solitary rodents, as the larger lagomorphs are selected for (Fig. 4d). There is likely some energetic trade-off in hunting small, scarce prey versus small, abundant prey or slightly larger prey including lagomorphs. The importance of the method used to determine coyote diet is counterintuitive and appears to reflect small sample sizes from the few studies that used both scats and stomachs, because the studies using these two methods alone revealed no differences (Fig. 4b).

We found a variety of influential factors affecting coyote selection for individual prey species. Mule deer and snowshoe hares are increasingly selected for in higher latitudes, whereas black-tailed jackrabbits are increasingly selected for in lower latitudes (Fig. 5). This variation in selection for individual species likely reflects the relative geographic distribution of these prey species as well as variation in susceptibility of each prey species in different conditions—in essence, this illustrates how biotic factors (i.e., predation) limit the realized niche of species.

The seasonal pattern of coyote prey selection that we detected may depict temporal patterns of prey availability as much as it does the susceptibility of prey to coyote predation. Prey species that become inactive at particular times of year (colder months), or become more inaccessible because of snow, disappear from the diet of coyotes entirely (e.g., Great Basin pocket mouse, and Ord’s kangaroo rat; Fig. 6). Conversely, mule deer appear to be increasingly susceptible to coyotes in the cooler months, as indicated in peaks in coyote selection (Fig. 6). This may be due to direct predation by coyotes or scavenging the carcasses of animals that died in the harsh seasonal conditions or during fall hunting seasons, although coyotes are likely more successful at hunting these larger prey when deep snow constrains prey movements (Gese and Grothe 1995).

Coyote density affected the selection for two prey species—mule deer and black-tailed jackrabbits—but with contrasting effects. As coyote density increased, so too did selection for mule deer, while black-tailed jackrabbits were avoided. These contrasting results might be the result of bottom-up factors affecting coyote densities. In the American West, the distribution of black-tailed jackrabbits and mule deer largely overlap, and both are common prey of coyotes in the region. However, food subsidies across the American West, in the form of livestock carrion and big game carcasses, result in elevated coyote densities, which ultimately may lead to suppressed hare numbers due to excessive predation by coyotes (Ripple et al. 2011, 2013). Consequently, higher densities of coyotes may become increasingly dependent on mule deer (the dominant ungulate in the region), via predation or hunter-killed carcasses, because the mule deer likely provide higher prey biomass compared to suppressed hare populations. Given the positive relationship between coyote density and pack size (Gese 2005), it could be argued that larger packs have more ability and need to hunt larger prey; however, more research is needed to test this hypothesis.

The presence of wolves (∑*w*_*i*_ = 0.30) had minimal impacts on coyote prey selection (∑*w*_*i*_ = 0.41; Table 1). Conversely, puma presence was related to higher prey selection (∑*w*_*i*_ = 0.76; Table 1). Although they are dominant to coyotes (Elbroch and Kusler 2018), the solitary nature of puma may mean that they are more easily displaced from carcasses that retain edible content than groups of wolves. In fact, research has shown that in some sites coyotes can obtain a majority of their diet from puma-killed prey, despite some coyotes being killed by pumas at carcasses (Ruprecht et al. 2021). Our results showed that eastern coyotes do not have stronger prey selection than pure coyotes, despite the former carrying wolf DNA (Way 2013). In contrast to previous findings, our results show that eastern coyotes do eat larger prey compared to pure coyotes (Jensen et al. 2022). While the presence of wolves did not substantially increase prey selection overall, there were insufficient data to investigate this for individual species beyond mule deer, which were consumed more frequently by coyotes when wolves were absent, and white-tailed deer and deer mice taken more when sympatric with wolves, at least in sites where prey abundance was available. We also caution that our results could have been an artifact of the current wolf distribution. Wolves are largely absent from the American West where mule deer are the primary ungulate, whereas wolves are present around the Great Lakes and southeastern Canada where white-tailed deer are the primary ungulate and deer mice are common. Other studies have shown that pumas and wolves perform a context-specific role of facilitating scavenging opportunities for coyotes (Wilmers et al. 2003a; Ruprecht et al. 2021), as reflected in the increased ungulate consumption when coyotes are sympatric with wolves (Jensen et al. 2022).

Coyotes consumed domestic sheep in proportion to their availability, supporting the notion that coyotes do not select domesticated sheep over wild prey. Our results support those of Sacks and Neale (2002), who found that coyotes killed sheep in proportion to their abundance within their territories, reinforcing that coyotes do not specialize on sheep. Our results demonstrate that sheep predation by coyotes can be reduced by augmenting natural prey numbers, especially those of their most selected prey. Augmenting natural prey numbers to reduce livestock also has been suggested for other carnivore species that select wild prey over sheep (Kamler et al. 2012; Odden et al. 2013; Burgas et al. 2014; Soofi et al. 2019; Cassaigne et al. 2021).

### Prey selection indices.

The variation attributable to the method of selection estimator is particularly interesting. It is heartening, given historical research using Jacobs’ index, that the traditional method yields results that are strongly related to the new IPA that accounts for species missing at individual sites (Nams and Hayward 2022). When we incorporate prey biomass and the likely number of scats this yields, correlation between methods decreases, although they are still significantly correlated. This suggests that prey selection studies relying on scats may overestimate the selection for larger species because one large individual may lead to up to 200 scats being produced by predators. Obviously, this assumes that the predator consumes the entire carcass (i.e., no intraguild competition at carcasses), and the researchers collect all available scats from the one location. Even if researchers collect only a couple of scats from the one location, but keep finding larger species in the diet of coyotes from sites further afield, this seems to suggest coyotes are selecting this larger species. That there is no difference in selection between scats and stomach contents analysis further suggests that this may not be a large problem, particularly given that morphological methods of diet determination from scats are challenging when no hair is included in the scats, which is common when larger animals are consumed. We have previously argued that the biases to large prey in scats are countered by underestimation of small prey species abundance (Hayward et al. 2006a). Finally, the fact that only two large species (>3 kg) were significantly selected (along with one weighing 1.2 kg and the other at 0.06 kg), while several others with similar ecological traits were not—for example, beavers (*Castor canadensis*), ground squirrels (*Sciuridae*), marmots (*Marmota* spp.), moose, pronghorn (*Antilocapra americana*), and mule deer (Fig. 3) reinforce this view. Nonetheless, more work is required to confirm our observation that these results are robust, and this could take the form of testing predictions of coyote diet as has been done for other predators (Hayward et al. 2007a).

There are spatial biases in our data set. Research of coyote diet is dominated by the largest three range countries (Fig. 1). Understanding coyote prey selection at its southern distribution limit in Guatemala, Belize, El Salvador, Honduras, Nicaragua, Costa Rica, and Panama would likely be informative given the different potential prey communities there. Nevertheless, while the coyote is one of the most studied carnivores in the world, we have provided the first formal assessment of the most selected prey species and the factors that influence prey selection have been identified, and they reinforce the ecological flexibility of coyotes. Ultimately, this information on coyotes can be used to predict their population sizes, home range sizes, and the impact of perturbations on their diet (Hayward et al. 2007a, 2007b, 2009).

## Supplementary Data

Supplementary data are available at *Journal of Mammalogy* online.

**Supplementary Data SD1.**—Study site details including Site reference, habitat, Bioregion, site name, map reference number, region/state, period, years, duration, country, method, sample size, wolf presence, puma presence, other carnivores, and study citation.

gyad094_suppl_Supplementary_Data_SD1

gyad094_suppl_Supplementary_Data_SD2

## Data Availability

Data used in this paper are stored in https://doi.org/10.5061/dryad.xgxd254k9.
