# Supplementary material for: Diet selection in the Coyote Canis latrans
Source: J Mammal. 2023 Nov 4;104(6):1338–52. doi: 10.1093/jmammal/gyad094 (PMC10697429; doi:10.1093/jmammal/gyad094)
Supplement: gyad094_suppl_Supplementary_Data_SD1 [file gyad094_suppl_supplementary_data_sd1.docx]

**Supplementary Materials Table S1. Study site details**

| Site # | Habitat | Bio-region | Site name | Map# Fig. 1 | Region / State | Period | Years | Duration | Country | Method | N | Citation |
| --- | --- | --- | --- | --- | --- | --- | --- | --- | --- | --- | --- | --- |
| 1 |  |  |  | 315 | Illinois | Autumn | 1993-1994 | 2 | USA |  |  | [1] |
| 2 |  |  |  | 318 | Illinois | Winter | 1993-1994 | 2 | USA |  |  | [1] |
| 3 |  |  |  | 316 | Illinois | Spring | 1993-1994 | 2 | USA |  |  | [1] |
| 4 |  |  |  | 317 | Illinois | Summer | 1993-1994 | 2 | USA |  |  | [1] |
| 5 |  |  |  | 319 | Illinois | Autumn | 1994-1995 | 2 | USA |  |  | [1] |
| 6 |  |  |  | 322 | Illinois | Winter | 1994-1995 | 2 | USA |  |  | [1] |
| 7 |  |  |  | 320 | Illinois | Spring | 1994-1995 | 2 | USA |  |  | [1] |
| 8 |  |  |  | 321 | Illinois | Summer | 1994-1995 | 2 | USA |  |  | [1] |
| 9 | Rural |  |  | 661 | West Virginia | Annual | 2009-2011 | 3 | USA | Scats | 693 | [2] |
| 10 | Rural |  |  | 662 | West Virginia | Annual | 2009-2011 | 3 | USA | Stomachs | 276 | [2] |
| 11 | Rural |  | Steuben | 483 | New York | Summer | 2007 | 0.5 | USA | Scats | 89 | [3] |
| 12 | Rural |  | Steuben | 485 | New York | Winter | 2007-2008 | 0.5 | USA | Scats | 108 | [3] |
| 13 | Rural |  | Steuben | 484 | New York | Summer | 2008 | 0.5 | USA | Scats | 65 | [3] |
| 14 | Rural |  | Oswego | 479 | New York | Summer | 2007 | 0.5 | USA | Scats | 94 | [3] |
| 15 | Rural |  | Oswego | 481 | New York | Winter | 2007-2008 | 0.5 | USA | Scats | 70 | [3] |
| 16 | Rural |  | Oswego | 480 | New York | Summer | 2008 | 0.5 | USA | Scats | 101 | [3] |
| 17 | Rural |  | Callaghan Ranch | 568 | Texas | Autumn | 1974 | 0.25 | USA | Scats | 81 | [4] |
| 18 | Rural |  | Callaghan Ranch | 574 | Texas | Winter | 1974-5 | 0.25 | USA | Scats | 232 | [4] |
| 19 | Rural |  | Callaghan Ranch | 570 | Texas | Spring | 1975 | 0.25 | USA | Scats | 318 | [4] |
| 20 | Rural |  | Callaghan Ranch | 571 | Texas | Summer | 1975 | 0.25 | USA | Scats | 170 | [4] |
| 21 | Rural |  | Callaghan Ranch | 569 | Texas | Autumn | 1975 | 0.25 | USA | Scats | 152 | [4] |
| 22 | Rural |  | Callaghan Ranch | 575 | Texas | Winter | 1975-1976 | 0.25 | USA | Scats | 589 | [4] |
| 23 | Rural |  | Callaghan Ranch | 572 | Texas | Spring | 1976 | 0.25 | USA | Scats | 287 | [4] |
| 24 | Rural |  | Callaghan Ranch | 573 | Texas | Summer | 1976 | 0.25 | USA | Scats | 77 | [4] |
| 25 | Rural |  | Killam Ranch | 580 | Texas | Autumn | 1976 | 0.25 | USA | Scats | 50 | [4] |
| 26 | Rural |  | Killam Ranch | 583 | Texas | Winter | 1976-1977 | 0.25 | USA | Scats | 210 | [4] |
| 27 | Rural |  | Killam Ranch | 581 | Texas | Spring | 1977 | 0.25 | USA | Scats | 59 | [4] |
| 28 | Rural |  | Killam Ranch | 582 | Texas | Summer | 1977 | 0.25 | USA | Scats | 54 | [4] |
| 29 | Rural |  | Central New York | 476 | New York | Winter | 1986-1989 | 0.75 | USA | Scats | 143 | [5] |
| 30 | Rural |  | Central New York | 474 | New York | Spring | 1986-1989 | 0.75 | USA | Scats | 385 | [5] |
| 31 | Rural |  | Central New York | 475 | New York | Summer | 1986-1989 | 0.75 | USA | Scats | 284 | [5] |
| 32 | Rural |  | Central New York | 473 | New York | Autumn | 1986-1989 | 0.75 | USA | Scats | 195 | [5] |
| 33 | Rural |  | Brush | 108 | Dur, Coa, Chi | Annual | 2000 | 1 | Mexico | Scats | 32 | [6] |
| 34 | Rural |  | Brush | 109 | Dur, Coa, Chi | Annual | 2002 | 1 | Mexico | Scats | 49 | [6] |
| 35 | Rural |  | Pasture | 111 | Dur, Coa, Chi | Annual | 2002 | 1 | Mexico | Scats | 25 | [6] |
| 36 | Rural |  | Pasture | 112 | Dur, Coa, Chi | Annual | 2003 | 1 | Mexico | Scats | 24 | [6] |
| 37 | Rural |  | Brush | 110 | Dur, Coa, Chi | Annual | 2003 | 1 | Mexico | Scats | 40 | [6] |
| 38 | Rural |  | Northern Utah | 648 | Utah | Annual | 1968 | 1 | USA | Stomachs | 41 | [7] |
| 39 | Rural |  | Northern Utah | 650 | Utah | Summer | 1968-1970 | 3 | USA | Scats | 30 | [7] |
| 40 | Rural |  | Northern Utah | 649 | Utah | Fall | 1968-1970 | 3 | USA | Scats | 69 | [7] |
| 41 | Rural |  | Joshua Tree National Monument | 211 | California | Annual | 1976-1978 | 3 | USA | Scats | 215 | [8] |
| 42 | Rural |  | North-central | 502 | Oklahoma | Spring | 1952 | 0.25 | USA | Scats | 86 | [9] |
| 43 | Rural |  | North-central | 503 | Oklahoma | Summer | 1952 | 0.25 | USA | Scats | 53 | [9] |
| 44 | Rural |  | North-central | 501 | Oklahoma | Fall | 1952 | 0.25 | USA | Scats | 87 | [9] |
| 45 | Rural |  | North-central | 504 | Oklahoma | Winter | 1952-1953 | 0.25 | USA | Scats | 132 | [9] |
| 46 | Rural |  | Cat Island | 525 | South Carolina | Annual | 2009-2011 | 3 | USA | Scats | 106 | [10] |
| 47 | Rural |  | South Island | 532 | South Carolina | Annual | 2009-2011 | 3 | USA | Scats | 199 | [10] |
| 48 | Rural |  | San Joaquin Experimental Research Station | 242 | California | Annual | 1939-1941 | 3 | USA | Scats | 1173 | [11] |
| 49 | Rural |  | Western Tennessee | 554 | Tennessee | Winter | 1990-1992 | 0.5 | USA | Scats | 75 | [12] |
| 50 | Rural |  | Western Tennessee | 552 | Tennessee | Spring | 1990-1992 | 0.5 | USA | Scats | 73 | [12] |
| 51 | Rural |  | Western Tennessee | 553 | Tennessee | Summer | 1990-1992 | 0.5 | USA | Scats | 94 | [12] |
| 52 | Rural |  | Western Tennessee | 551 | Tennessee | Fall | 1990-1992 | 0.5 | USA | Scats | 88 | [12] |
| 53 | Rural |  | Roy | 421 | Montana | Annual | 1976 | 1 | USA | Scat/Stomach | 39 | [13, 14] |
| 54 | Rural |  | Roy | 422 | Montana | Annual | 1977 | 1 | USA | Scats | 73 | [13, 14] |
| 55 | Rural |  | Roy | 423 | Montana | Annual | 1978 | 1 | USA | Scats | 97 | [13, 14] |
| 56 | Rural |  | Roy | 424 | Montana | Annual | 1979 | 1 | USA | Scats | 130 | [13, 14] |
| 57 | Rural |  | Roy | 425 | Montana | Annual | 1980 | 1 | USA | Scats | 61 | [13, 14] |
| 58 | Rural |  | Roy | 426 | Montana | Annual | 1981 | 1 | USA | Scats | 51 | [13, 14] |
| 59 | Rural |  | University of Texas field station (Control) | 621 | Texas | Annual | 1990 | 1 | USA | Stomachs | 30 | [15, 16] |
| 60 | Rural |  | Curlew Valley | 299 | Idaho | Annual | 1973-1975 | 3 | USA | Scats | 666 | [17] |
| 61 | Rural | 10.1.5 | Robinson Mountain | 441 | Nevada | Summer | 1975 | 0.25 | USA | Scats | 73 | [18] |
| 62 | Rural | 10.1.5 | Garden Valley | 440 | Nevada | Winter | 1975-1976 | 0.3 | USA | Scats | 198 | [18] |
| 63 | Rural | 10.1.5 | Garden Valley | 439 | Nevada | May | 1976 | 0.0833 | USA | Scat/Stomach | 40 | [18] |
| 64 | Rural | 10.1.5 | Schell Creek Range | 442 | Nevada | Summer | 1976 | 0.25 | USA | Scats | 137 | [18] |
| 65 | Rural | 10.1.5 | Milk River Range | 16 | Alberta | Annual | 1994-1996 | 3 | Canada | Scats | 333 | [19] |
| 66 | Rural | 11.1.1 | San Francisco State Fish and Wildlife Refuge | 240 | California | Winter | 2000 | 0.25 | USA | Scats | 104 | [20] |
| 67 | Rural | 11.1.1 | San Francisco State Fish and Wildlife Refuge | 238 | California | Spring | 2000 | 0.25 | USA | Scats | 99 | [20] |
| 68 | Rural | 11.1.1 | San Francisco State Fish and Wildlife Refuge | 239 | California | Summer | 2000 | 0.25 | USA | Scats | 92 | [20] |
| 69 | Rural | 5.3.1 | Moosehead Plateau | 379 | Maine | Annual | 1979-1982 | 4 | USA | Scats | 531 | [21, 22] |
| 70 | Wilderness | 5.4.1 | Riding Mountain National Park | 48 | Manitoba | Annual | 1975-1979 | 5 | Canada | Scats | 1265 | [23] |
| 71 | Rural | 8.3.7 | Pineywoods | 604 | Texas | Annual | 2009-2011 | 3 | USA | Scats | 841 | [24] |
| 72 | Wilderness | 6.1.5 | Kluane Boreal Forest Research Project | 85 | Yukon | Winter | 1987 | 0.25 | Canada | Scats | 102 | [25, 26] |
| 73 | Wilderness | 6.1.5 | Kluane Boreal Forest Research Project | 86 | Yukon | Winter | 1988 | 0.25 | Canada | Scats | 60 | [25, 26] |
| 74 | Wilderness | 6.1.5 | Kluane Boreal Forest Research Project | 87 | Yukon | Winter | 1989 | 0.25 | Canada | Scats | 39 | [25, 26] |
| 75 | Wilderness | 6.1.5 | Kluane Boreal Forest Research Project | 88 | Yukon | Winter | 1990 | 0.25 | Canada | Scats | 55 | [25, 26] |
| 76 | Wilderness | 6.1.5 | Kluane Boreal Forest Research Project | 89 | Yukon | Winter | 1991 | 0.25 | Canada | Scats | 30 | [25, 26] |
| 77 | Wilderness | 6.1.5 | Kluane Boreal Forest Research Project | 90 | Yukon | Winter | 1992 | 0.25 | Canada | Scats | 31 | [25, 26] |
| 78 | Wilderness | 6.1.5 | Kluane Boreal Forest Research Project | 91 | Yukon | Winter | 1993 | 0.25 | Canada | Scats | 32 | [25, 26] |
| 79 | Wilderness | 6.1.5 | Kluane Boreal Forest Research Project | 92 | Yukon | Winter | 1994 | 0.25 | Canada | Scats | 39 | [25, 26] |
| 80 | Rural | 9.2.1 | Harcus | 45 | Manitoba | Summer | 1971 | 0.25 | Canada | Scats | 25 | [27] |
| 81 | Rural | 9.2.1 | Spruce Woods | 49 | Manitoba | Summer | 1971 | 0.25 | Canada | Scats | 49 | [27] |
| 82 | Rural | 9.2.1 | Harcus | 46 | Manitoba | Spring | 1972 | 0.25 | Canada | Scats | 64 | [27] |
| 83 | Rural | 9.2.1 | Harcus | 47 | Manitoba | Summer | 1972 | 0.25 | Canada | Scats | 58 | [27] |
| 84 | Rural | 9.2.1 | Spruce Woods | 50 | Manitoba | Summer | 1972 | 0.25 | Canada | Scats | 86 | [27] |
| 85 | Wilderness | 6.1.2 | Central Alaska Range | 156 | Alaska | Annual | 1999-2000 | 1 | USA | Scats | 376 | [28-30] |
| 86 | Wilderness | 6.1.2 | Central Alaska Range | 157 | Alaska | Annual | 2000-2001 | 1 | USA | Scats | 537 | [28-30] |
| 87 | Wilderness | 6.1.2 | Central Alaska Range | 158 | Alaska | Annual | 2001-2002 | 1 | USA | Scats | 499 | [28-30] |
| 88 | Rural | 9.6.1 | Brackettville 26 | 567 | Texas | Summer | 1974 | 0.25 | USA | Scats | 31 | [31] |
| 89 | Rural | 9.6.1 | Brackettville 14 | 563 | Texas | Fall | 1974 | 0.25 | USA | Scats | 24 | [31] |
| 89 | Rural | 9.6.1 | Brackettville 25 | 566 | Texas | Fall | 1974 | 0.25 | USA | Scats | 24 | [31] |
| 90 | Rural | 9.6.1 | Brackettville 12 | 562 | Texas | Winter | 1974-5 | 0.25 | USA | Scats | 26 | [31] |
| 91 | Rural | 9.6.1 | Brackettville 2 | 565 | Texas | Summer | 1974 | 0.25 | USA | Scats | 28 | [31] |
| 92 | Rural | 9.6.1 | Brackettville 2 | 564 | Texas | Fall | 1974 | 0.25 | USA | Scats | 23 | [31] |
| 93 | Rural | 9.6.1 | Nuevo Leon | 126 | Mexico | Fall | 1997-1998 | 0.25 | Mexico | Scats | 488 | [32] |
| 94 | Rural | 7.1.3 | Kenai Peninsula | 166 | Alaska | Winter | 1987-1990 | 1.25 | USA | Scats | 179 | [33] |
| 95 | Rural | 7.1.3 | Kenai Peninsula | 165 | Alaska | Summer | 1987-1990 | 1.25 | USA | Scats | 183 | [33] |
| 96 | Rural | 7.1.3 | Kenai Peninsula | 164 | Alaska | Winter | 1987-1988 | 0.4167 | USA | Scats | 28 | [33] |
| 97 | Rural | 7.1.3 | Kenai Peninsula | 161 | Alaska | Summer | 1988 | 0.4167 | USA | Scats | 109 | [33] |
| 98 | Rural | 7.1.3 | Kenai Peninsula | 167 | Alaska | Winter | 1988-1989 | 0.4167 | USA | Scats | 97 | [33] |
| 99 | Rural | 7.1.3 | Kenai Peninsula | 162 | Alaska | Summer | 1989 | 0.4167 | USA | Scats | 67 | [33] |
| 100 | Rural | 7.1.3 | Kenai Peninsula | 168 | Alaska | Winter | 1989-1990 | 0.4167 | USA | Scats | 54 | [33] |
| 101 | Rural | 7.1.3 | Kenai Peninsula | 163 | Alaska | Winter | 1990 | 0.4167 | USA | Scats | 7 | [33] |
| 102 | Rural | 10.1.2 | Washington | 634 | USA | Summer | 1974 | 0.0027 | USA | Scats | 47 | [34] |
| 103 | Rural | 8.1.2 | Long Point | 67 | Ontario | Winter | 1984-1985 | 0.25 | Canada | Scats | 60 | [35] |
| 104 | Rural | 8.1.2 | Long Point | 65 | Ontario | Autumn | 1984-1985 | 0.25 | Canada | Scats | 130 | [35] |
| 105 | Rural | 8.1.2 | Long Point | 66 | Ontario | Spring | 1984-1985 | 0.25 | Canada | Scats | 160 | [35] |
| 106 | Rural | 5.4.1 | Rochester | 27 | Alberta | Spring | 1965-1968 | 0.25 | Canada | Scats | 20 | [36] |
| 107 | Rural | 5.4.1 | Rochester | 25 | Alberta | Spring | 1973 | 0.25 | Canada | Scats | 41 | [36] |
| 108 | Rural | 5.4.1 | Rochester | 17 | Alberta | Summer | 1964 | 0.25 | Canada | Scats | 45 | [36] |
| 109 | Rural | 5.4.1 | Rochester | 21 | Alberta | Summer | 1966 | 0.25 | Canada | Scats | 48 | [36] |
| 110 | Rural | 5.4.1 | Rochester | 19 | Alberta | Summer | 1965 | 0.25 | Canada | Scats | 48 | [36] |
| 111 | Rural | 5.4.1 | Rochester | 23 | Alberta | Summer | 1972 | 0.25 | Canada | Scats | 92 | [36] |
| 112 | Rural | 5.4.1 | Rochester | 26 | Alberta | Summer | 1973 | 0.25 | Canada | Scats | 51 | [36] |
| 113 | Rural | 5.4.1 | Rochester | 18 | Alberta | Fall | 1965 | 0.25 | Canada | Scats | 21 | [36] |
| 114 | Rural | 5.4.1 | Rochester | 20 | Alberta | Fall | 1966 | 0.25 | Canada | Scats | 67 | [36] |
| 115 | Rural | 5.4.1 | Rochester | 22 | Alberta | Fall | 1972 | 0.25 | Canada | Scats | 288 | [36] |
| 116 | Rural | 5.4.1 | Rochester | 24 | Alberta | Fall | 1973 | 0.25 | Canada | Scats | 35 | [36] |
| 117 | Rural | 5.4.1 | Rochester | 28 | Alberta | Winter | 1971-1972 | 0.25 | Canada | Scats | 101 | [36] |
| 118 | Rural | 5.4.1 | Rochester | 29 | Alberta | Winter | 1972-1973 | 0.25 | Canada | Scats | 339 | [36] |
| 119 | Rural | 5.4.1 | Rochester | 30 | Alberta | Winter | 1973-1974 | 0.25 | Canada | Scats | 176 | [36] |
| 120 | Rural | 5.4.1 | Rochester | 31 | Alberta | Winter | 1974-1975 | 0.25 | Canada | Scats | 37 | [36] |
| 121 | Rural | 6.2.10 | Potholes | 683 | Wyoming | Summer | 1973-1974 | 0.25 | USA | Scats | 185 | [37] |
| 122 | Rural | 6.2.10 | National Elk Refuge | 680 | Wyoming | Summer | 1973 | 0.25 | USA | Scats | 259 | [37] |
| 123 | Rural | 6.2.10 | National Elk Refuge | 679 | Wyoming | Fall | 1973 | 0.25 | USA | Scats | 90 | [37] |
| 124 | Rural | 6.2.10 | National Elk Refuge | 682 | Wyoming | Summer | 1974 | 0.25 | USA | Scats | 192 | [37] |
| 125 | Rural | 6.2.10 | National Elk Refuge | 681 | Wyoming | Fall | 1974 | 0.25 | USA | Scats | 80 | [37] |
| 126 | Rural | 6.2.10 | Shadow Mountain | 685 | Wyoming | Summer | 1973 | 0.25 | USA | Scats | 61 | [37] |
| 127 | Rural | 6.2.10 | Shadow Mountain | 684 | Wyoming | Fall | 1973 | 0.25 | USA | Scats | 23 | [37] |
| 128 | Rural | 6.2.10 | Shadow Mountain | 686 | Wyoming | Summer | 1974 | 0.25 | USA | Scats | 35 | [37] |
| 129 | Rural | 9.4.2 | Rolling Plains Quail Research Ranch | 610 | Texas | Annual | 2008-09 | 1 | USA | Scats | 380 | [38] |
| 130 | Rural | 9.4.2 | Rolling Plains Quail Research Ranch | 608 | Texas | Annual | 2010 | 1 | USA | Scats | 350 | [38] |
| 131 | Rural | 9.4.2 | Rolling Plains Quail Research Ranch | 609 | Texas | Annual | 2011 | 1 | USA | Scats | 350 | [38] |
| 132 | Rural | 9.5.1 | Welder Wildlife Refuge | 630 | Texas | Annual | 1961-1962 | 1 | USA | Scats | 2590 | [37] |
| 133 | Rural | 9.5.1 | Welder Wildlife Refuge | 631 | Texas | Annual | 1973-1974 | 1 | USA | Scats | 570 | [37] |
| 134 | Rural | 9.5.1 | Welder Wildlife Refuge | 632 | Texas | Annual | 1975-1976 | 1 | USA | Scats | 584 | [37] |
| 135 | Rural | 8.3.3 | Land between the lakes | 363 | KY TN | Summer | 1985 | 0.083 | USA | Scats & Stomach | 31 | [39] |
| 136 | Rural | 8.3.5 | Sumtner Farm | 153 | Alabama | Summer | 1985 | 0.083 | USA | Scats & Stomach | 34 | [39] |
| 137 | Rural | 8.3.3 | TWMA | 154 | Alabama | Summer | 1986 | 0.083 | USA | Scats & Stomach | 64 | [39] |
| 138 | Rural | 8.3.5 | DSWMA | 405 | Mississippi | Summer | 1985 | 0.083 | USA | Scats & Stomach | 48 | [39] |
| 139 | Rural | 8.3.5 | PCSA | 406 | Mississippi | Summer | 1986 | 0.083 | USA | Scats & Stomach | 24 | [39] |
| 140 | Rural | 8.3.3 | BWWMA | 150 | Alabama | Summer | 1986 | 0.083 | USA | Scats & Stomach | 28 | [39] |
| 141 | Rural | 9.4.4 | KPRNA | 331 | Kansas | june - December | 1988 | 0.5 | USA | Scats | 222 | [40] |
| 142 | Rural | 9.4.4 | Konza Prairie RNA | 332 | Kansas | ANNUAL | 1989 | 1 | USA | Scats | 476 | [40] |
| 143 | Rural | 9.4.4 | Konza Prairie RNA | 333 | Kansas | ANNUAL | 1990 | 1 | USA | Scats | 607 | [40] |
| 144 | Rural | 9.4.4 | Konza Prairie RNA | 334 | Kansas | ANNUAL | 1991 | 1 | USA | Scats | 516 | [40] |
| 145 | Rural | 10.2.1 | Naval Petroleum Reserve | 233 | California | ANNUAL | 1985 | 1 | USA | Stomach | 24 | [41] |
| 146 | Rural | 10.2.1 | Naval Petroleum Reserve | 234 | California | ANNUAL | 1986 | 1 | USA | Stomach | 33 | [41] |
| 147 | Rural | 9.2.4 | University of Kansas Fitch Natural History Reservation | 358 | Kansas | 4 years | 1948-1952 | 4 | USA | Scats | 118 | [42] |
| 148 | Rural | 10.1.8 | Sheep Experiment Station | 310 | Idaho | Winter | 1976-1978 | 0.5 | USA | Scats | 64 | [43] |
| 149 | Rural | 8.3.6 | AMES | 540 | Tennessee | Summer | 1993 | 0.25 | USA | Scats | 83 | [44] |
| 150 | Rural | 8.3.3 | LBL | 542 | Tennessee | Summer | 1993 | 0.25 | USA | Scats | 77 | [44] |
| 151 | Rural | 8.3.3 | Milan | 544 | Tennessee | Summer | 1993 | 0.25 | USA | Scats | 93 | [44] |
| 152 | Rural | 8.3.6 | Ames | 539 | Tennessee | Fall | 1993 | 0.25 | USA | Scats | 55 | [44] |
| 153 | Rural | 8.3.3 | LBL | 541 | Tennessee | Fall | 1993 | 0.25 | USA | Scats | 45 | [44] |
| 154 | Rural | 8.3.3 | Milan | 543 | Tennessee | Fall | 1993 | 0.25 | USA | Scats | 46 | [44] |
| 155 | Urban |  | Los Angeles | 220 | California | Dry | 2016-2018 | 3 | USA | Scats | 848 | [45] |
| 156 | Urban |  | Los Angeles | 222 | California | Wet | 2016-2018 | 3 | USA | Scats | 693 | [45] |
| 157 | Suburban |  | Los Angeles | 221 | California | Dry | 2016-2018 | 3 | USA | Scats | 818 | [45] |
| 158 | Rural |  | Kenauk Nature | 76 | Quebec | Summer | 2016 | 0.25 | Canada | Scats | 50 | [46] |
| 159 | Rural |  | Cape Breton Highlands National Park | 63 | Nova Scotia | Fall | 2012-2013 | 0.4 | Canada | Scats | 40 | [47] |
| 160 | Rural |  | Cape Breton Highlands National Park | 64 | Nova Scotia | Spring | 2012-2013 | 0.4 | Canada | Scats | 64 | [47] |
| 161 | Rural |  | Pend Oreille and Stevens Counties | 656 | Washington | Annual | 2015-2017 | 1 | USA | DNA | 103 | [48] |
| 162 | Suburban |  | Rockefeller State Park and Preserve | 482 | New York | Annual | 2017-2018 | 1 | USA | Scats | 59 | [49] |
| 163 | Rural | 10.2.4 | Reserva de la Biosfera de Mapimi | 136 | Pasture habitat, Chihuahua | Annual | 2003 | 1 | Mexico | Scats | 24 | [6] |
| 164 | Rural | 10.2.4 | Reserva de la Biosfera de Mapimi | 103 | Chihuahua | Annual | 2003 | 1 | Mexico | Scats | 40 | [6] |
| 165 | Rural | 10.2.4 | Reserva de la Biosfera de Mapimi | 135 | Chihuahua | Annual | 2002 | 1 | Mexico | Scats | 25 | [6] |
| 166 | Rural | 10.2.4 | Reserva de la Biosfera de Mapimi | 102 | Chihuahua | Annual | 2002 | 1 | Mexico | Scats | 49 | [6] |
| 167 | Rural | 10.2.4 | Reserva de la Biosfera de Mapimi | 101 | Chihuahua | Annual | 2000 | 1 | Mexico | Scats | 32 | [6] |
| 168 | Wilderness | 10.2.4 | Reserva de la Biosfera de Mapimi | 120 | Chihuahua | Annual | 2000-2007 | 8 | Mexico | Scats | 182 | [50] |
| 169 | Wilderness | 10.2.4 | Reserva de la Biosfera de Mapimi | 146 | Chihuahua | Annual | 2000-2007 | 8 | Mexico | Scats | 182 | [50] |
| 170 | Rural | 10.1.8 | Idaho National Engineering Laboratory | 258 | Idaho | Jan-Jun | 1978 | 0.5 | USA | Scats | 48 | [51, 52] |
| 171 | Rural | 10.1.8 | Idaho National Engineering Laboratory | 633 | Idaho | Jan-Jun | 1978 | 0.5 | USA | Scats | 64 | [51, 52] |
| 172 | Rural | 5.3.1 | Kejimkujik National Park | 83 | Quebec | Winter | 1992-1997 | 1.3 | Canada | Scats | 133 | [53, 54] |
| 173 | Rural | 5.3.1 | Cape Breton Highlands National Park | 42 | Cape Breton | Winter | 1992-1997 | 1.3 | Canada | Scats | 133 | [53, 54] |
| 174 | Rural | 5.3.1 | St Lawrence River, Quebec | 43 | Quebec | Winter | 1996 | 0.25 | Canada | Scats | 32 | [55] |
| 175 | Rural | 5.3.1 | St Lawrence River | 78 | Quebec | Winter | 1997 | 0.25 | Canada | Scats | 123 | [55] |
| 176 | Rural | 5.3.1 | St Lawrence River, Quebec | 44 | Quebec | Winter | 1997 | 0.25 | Canada | Scats | 55 | [55] |
| 177 | Rural | 10.1.5 | Curlew Valley | 636 | Utah | Autumn | 1977 | 0.25 | USA | Scats | 56 | [56-58] |
| 178 | Rural | 10.1.5 | Curlew Valley | 637 | Utah | Autumn | 1979 | 0.25 | USA | Scats | 28 | [56-58] |
| 179 | Rural | 10.1.5 | Curlew Valley | 638 | Utah | Autumn | 1980 | 0.25 | USA | Scats | 58 | [56-58] |
| 180 | Rural | 10.1.5 | Curlew Valley | 639 | Utah | Autumn | 1981 | 0.25 | USA | Scats | 56 | [56-58] |
| 181 | Rural | 10.1.5 | Curlew Valley | 640 | Utah | Autumn | 1982 | 0.25 | USA | Scats | 57 | [56-58] |
| 182 | Rural | 10.1.5 | Curlew Valley | 641 | Utah | Autumn | 1983 | 0.25 | USA | Scats | 53 | [56-58] |
| 183 | Rural | 10.1.5 | Curlew Valley | 642 | Utah | Autumn | 1984 | 0.25 | USA | Scats | 52 | [56-58] |
| 184 | Rural | 10.1.5 | Curlew Valley | 643 | Utah | Autumn | 1985 | 0.25 | USA | Scats | 39 | [56-58] |
| 185 | Rural | 10.1.5 | Curlew Valley | 644 | Utah | Autumn | 1986 | 0.25 | USA | Scats | 56 | [56-58] |
| 186 | Rural | 8.3.3 | Regional | 545 | Tennessee | Annual | 1985-1986 | 2 | USA | Scats | 523 | [39] |
| 187 | Rural | 9.4.4 | North-central Kansas | 336 | Kansas | Annual | 1988 | 1 | USA | Scats | 222 | [40] |
| 188 | Rural | 9.4.4 | North-central Kansas | 337 | Kansas | Annual | 1989 | 1 | USA | Scats | 476 | [40] |
| 190 | Rural | 9.4.4 | North-central Kansas | 338 | Kansas | Annual | 1990 | 1 | USA | Scats | 607 | [40] |
| 191 | Rural | 9.4.4 | North-central Kansas | 339 | Kansas | Annual | 1991 | 1 | USA | Scats | 516 | [40] |
| 192 | Rural | 10.1.3 | GMU 73A | 300 | Idaho | Jan-Mar | 1998 | 0.25 | USA | Stomachs | 44 | [59, 60] |
| 193 | Rural | 10.1.8 | Idaho National Engineering Laboratory | 302 | Idaho | Spring | 1975 | 0.5 | USA | Scats | 143 | [61] |
| 194 | Rural | 10.1.8 | Idaho National Engineering Laboratory | 303 | Idaho | Winter | 1975 | 0.5 | USA | Scats | 152 | [61] |
| 195 | Rural | 10.1.8 | Idaho National Engineering Laboratory | 304 | Idaho | Spring | 1976 | 0.5 | USA | Scats | 115 | [61] |
| 196 | Rural | 10.1.8 | Idaho National Engineering Laboratory | 305 | Idaho | Summer | 1976 | 0.25 | USA | Scats | 54 | [61] |
| 197 | Rural | 10.1.8 | Idaho National Engineering Laboratory | 307 | Idaho | Winter | 1977 | 0.25 | USA | Scats | 216 | [61] |
| 198 | Rural | 10.1.8 | Idaho National Engineering Laboratory | 306 | Idaho | Spring | 1977 | 0.25 | USA | Scats | 82 | [61] |
| 199 | Rural | 8.3.4 | Grant WMU | 292 | Georgia | Spring | 2010 | 0.25 | USA | Scats | 55 | [62] |
| 200 | Rural | 8.3.4 | Grant WMU | 293 | Georgia | Summer | 2010 | 0.25 | USA | Scats | 89 | [62] |
| 201 | Rural | 8.3.4 | Grant WMU | 294 | Georgia | Summer Late | 2010 | 0.166666667 | USA | Scats | 89 | [62] |
| 202 | Rural | 8.3.4 | Grant WMU | 291 | Georgia | Autumn | 2010 | 0.25 | USA | Scats | 113 | [62] |
| 203 | Rural | 8.3.4 | Cedar Creek | 288 | Georgia | Spring | 2010 | 0.25 | USA | Scats | 22 | [62] |
| 204 | Rural | 8.3.4 | Cedar Creek | 289 | Georgia | Summer | 2010 | 0.25 | USA | Scats | 54 | [62] |
| 205 | Rural | 8.3.4 | Cedar Creek | 287 | Georgia | Autumn | 2010 | 0.25 | USA | Scats | 133 | [62] |
| 206 | Rural |  | Statewide | 408 | Missouri | Annual | 1948-1956 | 9 | USA | Scat/Stomach | 1096 | [63] |
| 207 | Rural | 10.1.5 | Dugmore Military Area | 646 | Utah | Annual | 1999-2001 | 3 | USA | Scats | 1139 | [64] |
| 208 | Wilderness | 10.2.4 | Big Bend National Park | 560 | Texas | Spring | 1972-1974 | 3 | USA | Scats | 92 | [65] |
| 209 | Wilderness | 10.2.4 | Big Bend National Park | 561 | Texas | Spring | 1980-1981 | 2 | USA | Scats | 129 | [65] |
| 210 | Rural | 6.2.3 | Northwest | 308 | Idaho | Annual | 1981-1982 | 2 | USA | Scats | 41 | [66] |
| 211 | Urban | 8.2.3 | Chicago | 312 | Illinois | Annual | 2000-2002 | 3 | USA | Scats | 1429 | [67, 68] |
| 212 | Rural | 5.2.1 | Northwest | 670 | Wisconsin | Annual | 1971 | 1 | USA | Scats | 415 | [69] |
| 213 | Rural | 5.2.1 | Northwest | 671 | Wisconsin | Annual | 1972 | 1 | USA | Scats | 556 | [69] |
| 214 | Rural | 5.2.1 | Northwest | 672 | Wisconsin | Annual | 1973 | 1 | USA | Scats | 537 | [69] |
| 215 | Rural | 5.4.1 | Rochester | 32 | Alberta | Summer | 1964 | 0.25 | Canada | Stomachs | 45 | [70] |
| 216 | Rural | 5.4.1 | Rochester | 34 | Alberta | Summer | 1965 | 0.25 | Canada | Stomachs | 48 | [70] |
| 217 | Rural | 5.4.1 | Rochester | 36 | Alberta | Summer | 1966 | 0.25 | Canada | Stomachs | 48 | [70] |
| 218 | Rural | 5.4.1 | Rochester | 33 | Alberta | Autumn | 1965 | 0.25 | Canada | Stomachs | 48 | [70] |
| 219 | Rural | 5.4.1 | Rochester | 35 | Alberta | Autumn | 1966 | 0.25 | Canada | Stomachs | 67 | [70] |
| 220 | Rural | 10.2.1 | Lokern Natural Area | 219 | California | Annual | 2001-2004 | 3 | USA | Scats | 396 | [70] |
| 221 | Wilderness | 5.3.1 | Gaspe Peninsula | 74 | Quebec | Annual | 1992 | 1 | Canada | Scats | 319 | [71] |
| 222 | Wilderness | 6.2.10 | National Bison Reserve | 409 | Montana | Autumn | 1972-1975 | 1 | USA | Scats | 315 | [72] |
| 223 | Wilderness | 6.2.10 | National Bison Reserve | 412 | Montana | Winter | 1972-1975 | 1 | USA | Scats | 250 | [72] |
| 224 | Wilderness | 6.2.10 | National Bison Reserve | 410 | Montana | Spring | 1972-1975 | 1 | USA | Scats | 211 | [72] |
| 225 | Wilderness | 6.2.10 | National Bison Reserve | 411 | Montana | Summer | 1972-1975 | 1 | USA | Scats | 164 | [72] |
| 226 | Rural | 10.1.4 | Haystack Mountains | 674 | Wyoming | Annual | 1977 | 1 | USA | Scats | 404 | [73, 74] |
| 227 | Wilderness | 6.1.4 | Shakwak Trench | 93 | Yukon | Annual | 1980-1982 | 3 | Canada | Scats | 119 | [75] |
| 228 | Rural | 9.4.1 | Statewide | 342 | Kansas | Winter | 1948 | 0.417 | USA | Stomachs | 130 | [76] |
| 229 | Rural | 9.4.1 | Statewide | 344 | Kansas | Winter | 1949 | 0.417 | USA | Stomachs | 175 | [76] |
| 230 | Rural | 9.4.1 | Statewide | 346 | Kansas | Winter | 1950 | 0.417 | USA | Stomachs | 222 | [76] |
| 231 | Rural | 9.4.1 | Statewide | 348 | Kansas | Winter | 1951 | 0.417 | USA | Stomachs | 136 | [76] |
| 232 | Rural | 9.4.1 | Statewide | 350 | Kansas | Winter | 1952 | 0.417 | USA | Stomachs | 128 | [76] |
| 233 | Rural | 9.4.1 | Statewide | 352 | Kansas | Winter | 1953 | 0.417 | USA | Stomachs | 80 | [76] |
| 234 | Rural | 5.4.1 | South-east | 37 | Alberta | Winter | 1974-1975 | 0.25 | Canada | Stomachs | 37 | [77] |
| 235 | Rural | 5.3.1 | St Lawrence River - mixed land use | 81 | Quebec | Spring | 1995 | 0.25 | Canada | Scats | 149 | [78] |
| 236 | Rural | 5.3.1 | St Lawrence River - forest | 79 | Quebec | Spring | 1995 | 0.25 | Canada | Scats | 97 | [78] |
| 237 | Rural | 5.3.1 | St Lawrence River - mixed land use | 82 | Quebec | Spring | 1995 | 0.25 | Canada | Scats | 119 | [78] |
| 238 | Rural | 5.3.1 | St Lawrence River - forest | 80 | Quebec | Spring | 1995 | 0.25 | Canada | Scats | 202 | [78] |
| 239 | Rural | 6.2.9 | Klah Klahnee Wildlife Sanctuary | 513 | Oregon | Annual | 1976 | 1 | USA | Scats | 308 | [79] |
| 240 | Rural | 11.1.2 | Carrizo Plain National Monument | 200 | California | Wet | 1989 | 0.5 | USA | Scats | 24 | [80] |
| 241 | Rural | 11.1.2 | Carrizo Plain National Monument | 199 | California | Dry | 1989 | 0.5 | USA | Scats | 91 | [80] |
| 242 | Rural | 11.1.2 | Carrizo Plain National Monument | 201 | California | Dry | 1990 | 0.5 | USA | Scats | 45 | [80] |
| 243 | Rural | 11.1.2 | Carrizo Plain National Monument | 203 | California | Dry | 1991 | 0.5 | USA | Scats | 45 | [80] |
| 244 | Rural | 11.1.2 | Carrizo Plain National Monument | 202 | California | Wet | 1990 | 0.5 | USA | Scats | 45 | [80] |
| 245 | Rural | 6.2.10 | Jackson Hole | 676 | Wyoming | Annual | 1998-1999 | 1 | USA | Scats | 169 | [81] |
| 246 | Suburban | 6.2.10 | Jackson Hole | 677 | Wyoming | Annual | 1998-1999 | 1 | USA | Scats | 170 | [81] |
| 247 | Rural | 9.6.1 | Laredo | 588 | Texas | Winter | 1979 | 0.25 | USA | Scats | 2335 | [82] |
| 248 | Rural | 9.6.1 | Laredo | 589 | Texas | Winter | 1980 | 0.25 | USA | Scats |  | [82] |
| 249 | Rural | 9.6.1 | Laredo | 590 | Texas | Winter | 1981 | 0.25 | USA | Scats |  | [82] |
| 250 | Rural | 9.6.1 | Laredo | 591 | Texas | Winter | 1982 | 0.25 | USA | Scats |  | [82] |
| 251 | Rural | 9.6.1 | Laredo | 592 | Texas | Winter | 1983 | 0.25 | USA | Scats |  | [82] |
| 252 | Rural | 9.6.1 | Laredo | 593 | Texas | Winter | 1984 | 0.25 | USA | Scats |  | [82] |
| 253 | Rural | 9.6.1 | Laredo | 594 | Texas | Winter | 1985 | 0.25 | USA | Scats |  | [82] |
| 254 | Rural | 9.6.1 | Laredo | 595 | Texas | Winter | 1986 | 0.25 | USA | Scats |  | [82] |
| 255 | Rural | 10.1.5 | Dugmore Military Area | 645 | Utah | Annual | 2013 | 3 | USA | Scats | 776 | [83] |
| 256 | Rural |  | Mojave Desert | 228 | California | Annual | 2009 | 1 | USA | Scats | 625 | [84] |
| 257 | Rural |  | Mojave Desert | 229 | California | Annual | 2010 | 1 | USA | Scats | 474 | [84] |
| 258 | Rural |  | Mojave Desert | 230 | California | Annual | 2011 | 1 | USA | Scats | 631 | [84] |
| 259 | Rural |  | Mojave Desert | 231 | California | Annual | 2012 | 1 | USA | Scats | 801 | [84] |
| 260 | Rural |  | Mojave Desert | 232 | California | Annual | 2013 | 1 | USA | Scats | 715 | [84] |
| 261 |  |  | Sierra de Tepotzotlán State Park | 148 | Estado de Mexico | Dry | 2008-2009 | 1 | Mexico | Scats | 57 | [85] |
| 262 |  |  | Sierra de Tepotzotlán State Park | 149 | Estado de Mexico | Wet | 2008-2009 | 1 | Mexico | Scats | 57 | [85] |
| 263 | Rural |  | Statewide | 284 | Florida | Annual | 2011-2015 | 5 | USA | Stomachs | 263 | [86] |
| 264 |  |  | Valles Caldera National Preserve | 457 | New Mexico | Spring | 2005 | 0.25 | USA | Scats | 39 | [87] |
| 265 |  |  | Valles Caldera National Preserve | 458 | New Mexico | Summer | 2005 | 0.25 | USA | Scats | 101 | [87] |
| 266 |  |  | Valles Caldera National Preserve | 456 | New Mexico | Autumn | 2005 | 0.25 | USA | Scats | 113 | [87] |
| 267 |  |  | Valles Caldera National Preserve | 462 | New Mexico | Winter | 2006 | 0.25 | USA | Scats | 80 | [87] |
| 268 |  |  | Valles Caldera National Preserve | 460 | New Mexico | Spring | 2006 | 0.25 | USA | Scats | 79 | [87] |
| 269 |  |  | Valles Caldera National Preserve | 461 | New Mexico | Summer | 2006 | 0.25 | USA | Scats | 105 | [87] |
| 270 |  |  | Valles Caldera National Preserve | 459 | New Mexico | Autumn | 2006 | 0.25 | USA | Scats | 120 | [87] |
| 271 |  |  | Valles Caldera National Preserve | 466 | New Mexico | Winter | 2007 | 0.25 | USA | Scats | 81 | [87] |
| 272 |  |  | Valles Caldera National Preserve | 464 | New Mexico | Spring | 2007 | 0.25 | USA | Scats | 114 | [87] |
| 273 |  |  | Valles Caldera National Preserve | 465 | New Mexico | Summer | 2007 | 0.25 | USA | Scats | 119 | [87] |
| 274 |  |  | Valles Caldera National Preserve | 463 | New Mexico | Autumn | 2007 | 0.25 | USA | Scats | 109 | [87] |
| 275 |  |  | Valles Caldera National Preserve | 470 | New Mexico | Winter | 2008 | 0.25 | USA | Scats | 56 | [87] |
| 276 |  |  | Valles Caldera National Preserve | 468 | New Mexico | Spring | 2008 | 0.25 | USA | Scats | 65 | [87] |
| 277 |  |  | Valles Caldera National Preserve | 469 | New Mexico | Summer | 2008 | 0.25 | USA | Scats | 101 | [87] |
| 278 |  |  | Valles Caldera National Preserve | 467 | New Mexico | Autumn | 2008 | 0.25 | USA | Scats | 103 | [87] |
| 279 |  |  | Gray's Lake National Wildlife Refuge | 301 | Idaho | Annual | 2003-2004 | 2 | USA | Scats | 117 | C.D. Mitchell unpubl. data |
| 280 |  |  | Anderson Mesa | 172 | Arizona | Winter | 1978 | 0.25 | USA | Scats | 65 | [88] |
| 281 |  |  | Anderson Mesa | 171 | Arizona | Summer | 1978 | 0.5 | USA | Scats | 102 | [88] |
| 282 |  |  | Fort Bragg Military Installation | 493 | North Carolina | Summer | 2011-2012 | 0.25 | USA | Scats | 77 | [89] |
| 283 |  |  | Fort Bragg Military Installation | 489 | North Carolina | Autumn | 2011-2012 | 0.25 | USA | Scats | 164 | [89] |
| 284 |  |  | Fort Bragg Military Installation | 495 | North Carolina | Winter | 2011-2012 | 0.25 | USA | Scats | 40 | [89] |
| 285 |  |  | Fort Bragg Military Installation | 491 | North Carolina | Spring | 2011-2012 | 0.25 | USA | Scats | 34 | [89] |
| 286 |  |  | Pend Oreille county | 657 | Washington | Annual | 2015-2017 | 3 | USA | Scats | 103 | [48] |
| 287 |  |  | Bounty area | 2 | Alberta | Spring | 2016-2017 | 0.5 | Canada | Scats | 60 | [90] |
| 288 |  |  | Bounty area | 3 | Alberta | Summer | 2016-2026 | 0.5 | Canada | Scats | 78 | [90] |
| 289 |  |  | Control | 7 | Alberta | Summer | 2016-2026 | 0.5 | Canada | Scats | 31 | [90] |
| 290 |  |  | Control | 6 | Alberta | Spring | 2016-2026 | 0.5 | Canada | Scats | 35 | [90] |
| 291 | Wilderness |  | Denali National Park | 159 | Alaska | Winter | 2013-2014 | 0.5 | USA | Scats | 106.5 | [91] |
| 292 | Wilderness |  | Susitna River Basin | 169 | Alaska | Winter | 2013-2014 | 0.5 | USA | Scats | 106.5 | [91] |
| 293 | Wilderness |  | Interface - Midpeninsula Regional Open Space District | 210 | California | Annual | 2014-2015 | 0.33 | USA | Scats | 152 | [92] |
| 293 | Wilderness |  | Wildland - Midpeninsula Regional Open Space District | 257 | California | Annual | 2014-2015 | 0.33 | USA | Scats | 152 | [92] |
| 294 | Urban |  | Los Angeles - urban | 226 | California | Wet | 2016-2018 | 1 | USA | Scats | 693 | [93] |
| 295 | Urban |  | Los Angeles - urban | 225 | California | Dry | 2016-2018 | 1 | USA | Scats | 848 | [93] |
| 296 | Suburban |  | Los Angeles - suburban | 224 | California | Wet | 2016-2018 | 1 | USA | Scats | 788 | [93] |
| 298 | Rural |  | Joseph Jones Ecological Research Centre | 295 | Georgia | Annual | 2007-2012 | 4 | USA | Scats | 673 | [94] |
| 299 | Rural |  | San Luis Gonzaga Island | 95 | Baja California | Spring | 1997 | 0.25 | Mexico | Scats | 239 | [95] |
| 300 | Rural | 9.2.3 | Southern counties | 323 | Iowa | Winter | 1972-1973 | 0.33 | USA | Stomachs | 222 | [96] |
| 301 | Rural | 5.1.4 | Newfoundland Island | 62 | Newfoundland | Winter | 2008-2009 | 0.5 | Canada | Stomachs | 292 | [97] |
| 302 | Rural | 13.4.2 | Sierra del Ajusco | 128 | Morales | Annual | 1992-1993 | 1 | Mexico | Scats | 238 | [98] |
| 303 | Wilderness | 6.2.4 | North Fork, Flathead River | 413 | Montana | Summer | 1994 | 0.5 | USA | Scats | 23 | [99] |
| 304 | Wilderness | 6.2.4 | North Fork, Flathead River | 414 | Montana | Winter | 1994 | 0.5 | USA | Scats | 26 | [99] |
| 305 | Wilderness | 6.2.4 | North Fork, Flathead River | 415 | Montana | Summer | 1995 | 0.5 | USA | Scats | 33 | [99] |
| 306 | Wilderness | 6.2.4 | North Fork, Flathead River | 416 | Montana | Winter | 1995 | 0.5 | USA | Scats | 29 | [99] |
| 307 | Wilderness | 6.2.4 | North Fork, Flathead River | 417 | Montana | Summer | 1996 | 0.5 | USA | Scats | 77 | [99] |
| 308 | Wilderness | 6.2.4 | North Fork, Flathead River | 418 | Montana | Winter | 1996 | 0.5 | USA | Scats | 33 | [99] |
| 309 | Wilderness | 6.2.4 | North Fork, Flathead River | 419 | Montana | Winter | 1997 | 0.5 | USA | Scats | 43 | [99] |
| 310 | Rural | 10.2.3 | Cape region | 97 | Baja California Sur | Spring | 1986 | 0.25 | Mexico | Scats | 88 | [100] |
| 311 | Rural | 10.2.3 | Cape region | 98 | Baja California Sur | Summer | 1986 | 0.25 | Mexico | Scats | 134 | [100] |
| 312 | Rural | 10.2.3 | Cape region | 96 | Baja California Sur | Autumn | 1986 | 0.25 | Mexico | Scats | 76 | [100] |
| 313 | Rural | 10.2.3 | Cape region | 99 | Baja California Sur | Winter | 1987 | 0.25 | Mexico | Scats | 135 | [100] |
| 314 | Wilderness | 5.2.4 | Big Bend National Park | 559 | Texas | Annual | 1971-1972 | 1 | USA | Scats | 28 | [101] |
| 315 | Rural | 9.3.1 | Prairie Pothole region | 84 | Saskatchewan | Spring | 2001 | 0.25 | Canada | Stomachs | 21 | [102] |
| 316 | Rural |  | Statewide | 514 | Oregon | Annual | 1917-1920 | 3 | USA | Stomachs | 450 | [103] |
| 317 | Rural | 11.1.2 | San Joaquin River National Wildlife Reserve | 243 | California | Annual | 2013-2014 | 1 | USA | Scats | 123 | [104] |
| 318 | Rural | 11.1.2 | San Luis National Wildlife Refuge | 244 | California | Annual | 2013-2014 | 1 | USA | Scats | 127 | [104] |
| 319 | Rural | 11.1.2 | Merced National Wildlife Refuge | 227 | California | Annual | 2013-2014 | 1 | USA | Scats | 113 | [104] |
| 320 | Rural | 5.2.1 | Northern | 394 | Minnesota | Winter | 1968-1969 | 0.333 | USA | Stomachs | 95 | [105] |
| 321 | Rural | 5.2.1 | Northern | 395 | Minnesota | Winter | 1969-1970 | 0.333 | USA | Stomachs | 110 | [105] |
| 322 | Rural | 5.2.1 | Northern | 396 | Minnesota | Winter | 1970-1971 | 0.333 | USA | Stomachs | 126 | [105] |
| 323 | Rural | 5.2.1 | Northern | 397 | Minnesota | Winter | 1971-1972 | 0.333 | USA | Stomachs | 287 | [105] |
| 324 | Rural | 5.2.1 | Northern | 398 | Minnesota | Winter | 1972-1973 | 0.333 | USA | Stomachs | 93 | [105] |
| 325 | Rural | 5.2.1 | Northern | 399 | Minnesota | Winter | 1973-1974 | 0.333 | USA | Stomachs | 214 | [105] |
| 326 | Rural | 5.2.1 | Northern | 400 | Minnesota | Winter | 1974-1975 | 0.333 | USA | Stomachs | 99 | [105] |
| 327 | Rural | 5.2.1 | Northern | 401 | Minnesota | Winter | 1975-1976 | 0.333 | USA | Stomachs | 180 | [105] |
| 328 | Rural | 8.1.1 | South-eastern | 77 | Quebec | Annual | 1975-1977 | 3 | Canada | Stomachs | 84 | [106] |
| 329 | Rural | 9.4.2 | South-eastern | 507 | Oklahoma | Winter | 1970 | 0.25 | USA | Stomachs | 70 | [107] |
| 330 | Rural | 9.4.2 | South-eastern | 506 | Oklahoma | Spring | 1970 | 0.25 | USA | Stomachs | 28 | [107] |
| 331 | Rural | 9.4.2 | South-eastern | 505 | Oklahoma | Autumn | 1970 | 0.25 | USA | Stomachs | 24 | [107] |
| 332 | Rural | 8.4.1 | South-central | 521 | Pennsylvania | Annual | 1994-1995 | 1 | USA | Scats | 184 | [108] |
| 333 | Suburban | 8.1.7 | Westchester County | 487 | New York | Spring | 2006-2008 | 1 | USA | Scats | 169 | [109] |
| 334 | Suburban | 8.1.7 | Westchester County | 486 | New York | Autumn | 2006-2008 | 1 | USA | Scats | 111 | [109] |
| 335 | Wilderness | 5.3.1 | Gaspe Peninsula | 75 | Quebec | Annual | 2003-2004 | 1 | Canada | Scats | 100 | [110] |
| 336 | Suburban | 8.1.0 | Cuyahoga Valley National Park | 500 | Ohio | Annual | 2002-2003 | 3 | USA | Scats | 944 | [111] |
| 337 | Rural | 6.2.8 | Lava Beds National Monument | 217 | California | Annual | 1937-1938 | 1.083333333 | USA | Scats | 273 | [112] |
| 338 | Wilderness | 6.2.4 | Jasper National Park | 10 | Alberta | Summer | 1974 | 0.5 | Canada | Scats | 349 | [113, 114] |
| 339 | Wilderness | 6.2.4 | Jasper National Park | 12 | Alberta | Winter | 1975 | 0.5 | Canada | Scats | 357 | [113, 114] |
| 340 | Wilderness | 6.2.4 | Jasper National Park | 11 | Alberta | Summer | 1975 | 0.5 | Canada | Scats | 434 | [113, 114] |
| 341 | Wilderness | 6.2.4 | Jasper National Park | 14 | Alberta | Winter | 1976 | 0.5 | Canada | Scats | 370 | [113, 114] |
| 342 | Wilderness | 6.2.4 | Jasper National Park | 13 | Alberta | Summer | 1976 | 0.5 | Canada | Scats | 400 | [113, 114] |
| 343 | Wilderness | 6.2.4 | Jasper National Park | 15 | Alberta | Winter | 1977 | 0.5 | Canada | Scats | 40 | [113, 114] |
| 344 | Rural | 11.1.3 | Cuyamaca Rancho State Park | 204 | California | Annual | 1977-1979 | 1.5 | USA | Scats | 223 | [115] |
| 345 | Wilderness | 6.2.4 | North Fork, Flathead River - USA/Canada border | 420 | Montana | Annual | 1978-1981 | 3 | USA | Scats | 181 | [116] |
| 346 | Rural | 5.1.4 | Newfoundland Island | 60 | Newfoundland | Annual | 2001-2002 | 1 | Canada | Stomachs | 21 | [117] |
| 347 | Rural | 5.1.4 | Newfoundland Island | 61 | Newfoundland | Annual | 2002-2003 | 1 | Canada | Stomachs | 42 | [117] |
| 348 | Rural | 9.4.2 | Wilson Lake | 359 | Kansas | Annual | 1990-1991 | 1 | USA | Scats | 268 | [118] |
| 349 | Rural | 9.4.2 | Lucas Road | 335 | Kansas | Annual | 1990-1991 | 1 | USA | Scats | 552 | [118] |
| 350 | Rural | 9.4.2 | Count Road | 325 | Kansas | Annual | 1990-1991 | 1 | USA | Scats | 201 | [118] |
| 351 | Rural | 9.4.2 | Glen Elder | 330 | Kansas | Annual | 1990-1991 | 1 | USA | Scats | 368 | [118] |
| 352 | Urban | 8.2.3 | Chicago | 311 | Illinois | Annual | 1995-1997 | 2 | USA | Scats |  | [119] |
| 353 | Rural | 8.1.8 | East Maine | 371 | Maine | Winter | 1979-1981 | 0.75 | USA | Scats | 125 | [120] |
| 354 | Rural | 8.1.8 | East Maine | 369 | Maine | Autumn | 1979-1981 | 0.75 | USA | Scats | 236 | [120] |
| 355 | Rural | 8.1.8 | East Maine | 370 | Maine | Autumn | 1979-1981 | 0.75 | USA | Scats | 236 | [120] |
| 356 | Rural | 8.1.8 | West Maine | 391 | Maine | Winter | 1979-1981 | 0.75 | USA | Scats | 54 | [120] |
| 357 | Rural | 8.1.8 | West Maine | 390 | Maine | Summer | 1979-1981 | 0.75 | USA | Scats | 49 | [120] |
| 358 | Rural | 8.1.8 | West Maine | 389 | Maine | Autumn | 1979-1981 | 0.75 | USA | Scats | 42 | [120] |
| 359 | Urban | 8.1.10 | Cuyahoga Valley National Park | 499 | Ohio | Annual | 1998-1999 | 1.08 | USA | Scats | 50 | [121] |
| 360 | Rural | 6.2.10 | Wind Cave National Park | 538 | South Dakota | Annual | 2004 | 0.97 | USA | Scats | 46 | [122] |
| 361 | Wilderness | 6.2.4 | Banff National Park | 40 | British Columbia | Winter | 1942-1943 | 0.5 | Canada | Scats | 123 | [123] |
| 362 | Wilderness | 6.2.4 | Banff National Park | 39 | British Columbia | Summer | 1943 | 0.5 | Canada | Scats | 49 | [123] |
| 363 | Rural | 8.4.2 | Redbird | 362 | Kentucky | Annual | 1999-2001 | 3 | USA | Scats | 219 | [124] |
| 364 | Rural | 8.4.2 | Laurel Fork | 361 | Kentucky | Annual | 1999-2001 | 3 | USA | Scats | 261 | [124] |
| 365 | Wilderness | 6.2.10 | Lamar Valley, Yellowstone National Park | 678 | Wyoming | Annual | 1990-1995 | 6 | USA | Scats |  | [125] |
| 366 | Rural | 5.3.1 | Gaspe Peninsula | 73 | Quebec | Annual | 1991 | 1 | Canada | Scats | 301 | [126] |
| 367 | Rural | 8.4.2 | Randolph County | 659 | West Virginia | Summer | 2006-2008 | 1.5 | USA | Scats | 86 | [127] |
| 368 | Rural | 8.4.2 | Randolph County | 660 | West Virginia | winter | 2006-2008 | 1.5 | USA | Scats | 42 | [127] |
| 369 | Rural | 11.1.2 | Site 1, Fresno County | 254 | California | Annual | 1983-1985 | 3 | USA | Scats | 1117 | [128] |
| 370 | Rural | 11.1.2 | Site 2, Fresno County | 255 | California | Annual | 1983-1985 | 3 | USA | Scats | 1117 | [128] |
| 371 | Rural | 8.3.3 | Central | 360 | Kentucky | winter | 1986-1987 | 0.25 | USA | Scats | 60 | [129] |
| 372 | Rural | 13.5.2 | de la Sierra Norte de Oaxaca | 134 | Oaxaca | Wet | 2002-2003 | 0.25 | Mexico | Scats | 25 | [130] |
| 373 | Rural | 13.5.2 | de la Sierra Norte de Oaxaca | 133 | Oaxaca | Dry | 2002-2003 | 0.25 | Mexico | Scats | 48 | [130] |
| 374 | Wilderness | 13.4.2 | Parque Nacional Iztaccihuatl | 94 | Estado de Mexico | Annual | 2010 | 0.5 | Mexico | Scats | 328 | [131] |
| 375 | Rural | 13.1.1 | Mazatzal Mountains - unburnt | 187 | Arizona | Annual | 1996 | 1 | USA | Scats | 25 | [132] |
| 376 | Rural | 13.1.1 | Mazatzal Mountains - burnt | 185 | Arizona | Annual | 1998 | 1 | USA | Scats | 33 | [132] |
| 377 | Rural | 13.1.1 | Mazatzal Mountains - unburnt | 188 | Arizona | Annual | 1998 | 1 | USA | Scats | 21 | [132] |
| 378 | Rural | 13.1.1 | Mazatzal Mountains - unburnt | 189 | Arizona | Annual | 1999 | 1 | USA | Scats | 32 | [132] |
| 379 | Rural | 13.1.1 | Mazatzal Mountains - burnt | 186 | Arizona | Annual | 1999 | 1 | USA | Scats | 22 | [132] |
| 380 | Rural | 10.2.4 | Mapimi Biosphere Reserve | 106 | Chihuahua | Annual | 1981-1982 | 2 | Mexico | Scats | 53 | [133] |
| 381 | Wilderness | 5.3.1 | Pierce Pond | 388 | Maine | Winter | 1979-1983 | 1 | USA | Scats | 350 | [134] |
| 382 | Wilderness | 5.3.1 | Pierce Pond | 387 | Maine | Summer | 1979-1983 | 1 | USA | Scats | 350 | [134] |
| 383 | Wilderness | 5.3.1 | Pierce Pond | 386 | Maine | Autumn | 1979-1983 | 1 | USA | Scats | 350 | [134] |
| 384 | Wilderness | 5.3.1 | Cherryfield | 366 | Maine | Autumn | 1979-1983 | 1 | USA | Scats | 350 | [134] |
| 385 | Wilderness | 5.3.1 | Cherryfield | 368 | Maine | Winter | 1979-1983 | 1 | USA | Scats | 350 | [134] |
| 386 | Wilderness | 5.3.1 | Cherryfield | 367 | Maine | Summer | 1979-1983 | 1 | USA | Scats | 350 | [134] |
| 387 | Wilderness | 6.2.10 | Togwotee Pass | 687 | Wyoming | Winter | 2006-2008 | 1 | USA | Scats | 470 | [135, 136] |
| 388 | Rural | 10.2.2 | Santa Rita Experimental Range | 191 | Arizona | Annual | 1970-1973 | 4 | USA | Stomachs | 101 | [137] |
| 389 | Rural | 8.1.9 | Kouchibouguac National Park - outside | 53 | New Brunswick | Annual | 1996-1997 | 2 | Canada | Scats | 364 | [138, 139] |
| 390 | Rural | 8.1.9 | Kouchibouguac National Park - inside | 52 | New Brunswick | Annual | 1996-1997 | 2 | Canada | Scats | 364 | [138, 139] |
| 391 | Rural | 8.3.5 | Central | 404 | Mississippi | Annual | 1993-1995 | 3 | USA | Scats | 82 | [140] |
| 392 | Rural | 6.1.2 | Usibelli Coal Mine | 170 | Alaska | Annual | 1981-1982 | 2 | USA | Scats | 41 | [141] |
| 393 | Wilderness | 6.2.15 | River of No Return Wilderness Area | 309 | Idaho | Summer | 1977-1978 | 0.5 | USA | Scats | 51 | [142] |
| 394 | Rural | 13.5.1 | Sierra del Manantlan | 124 | Jalisco | Annual | 1988-1989 | 2 | Mexico | Scats | 88 | [143] |
| 395 | Rural | 8.5.3 | Cat Island | 526 | South Carolina | Annual | 2009-2011 | 3 | USA | Scats | 106 | [10] |
| 396 | Rural | 8.5.3 | South Island | 533 | South Carolina | Annual | 2009-2011 | 3 | USA | Scats | 199 | [10] |
| 398 | Suburban | 11.1.1 | Santa Monica Mountains - North | 247 | California | Summer | 1997-1998 | 1 | USA | Scats | 141 | [144, 145] |
| 399 | Suburban | 11.1.1 | Santa Monica Mountains - North | 248 | California | Winter | 1997-1998 | 1 | USA | Scats | 145 | [144, 145] |
| 400 | Suburban | 11.1.1 | Santa Monica Mountains - Central | 246 | California | Winter | 1997-1998 | 1 | USA | Scats | 113 | [144, 145] |
| 401 | Suburban | 11.1.1 | Santa Monica Mountains - Central | 245 | California | Summer | 1997-1998 | 1 | USA | Scats | 137 | [144, 145] |
| 402 | Suburban | 11.1.1 | Santa Monica Mountains - South | 249 | California | Summer | 1997-1998 | 1 | USA | Scats | 121 | [144, 145] |
| 403 | Suburban | 11.1.1 | Santa Monica Mountains - South | 250 | California | Winter | 1997-1998 | 1 | USA | Scats | 104 | [144, 145] |
| 404 | Rural | 9.3.4 | Sandhills | 437 | Nebraska | Winter | 1947-1951 | 1 | USA | Scats | 1189 | [146] |
| 405 | Rural | 9.3.4 | Sandhills | 435 | Nebraska | Spring | 1947-1951 | 1 | USA | Scats | 698 | [146] |
| 406 | Rural | 9.3.4 | Sandhills | 436 | Nebraska | Summer | 1947-1951 | 1 | USA | Scats | 176 | [146] |
| 407 | Rural | 9.3.4 | Sandhills | 434 | Nebraska | Autumn | 1947-1951 | 1 | USA | Scats | 96 | [146] |
| 408 | Rural | 9.4.2 | Loess Hills | 433 | Nebraska | Winter | 1947-1951 | 1 | USA | Scats | 41 | [146] |
| 409 | Rural | 9.4.2 | Loess Hills | 431 | Nebraska | Spring | 1947-1951 | 1 | USA | Scats | 102 | [146] |
| 410 | Rural | 9.4.2 | Loess Hills | 432 | Nebraska | Summer | 1947-1951 | 1 | USA | Scats | 28 | [146] |
| 411 | Rural | 9.4.2 | Loess Hills | 430 | Nebraska | Autumn | 1947-1951 | 1 | USA | Scats | 23 | [146] |
| 412 | Rural | 8.1.9 | Prince Edward Island | 69 | Prince Edward | Winter | 2001-2003 | 0.75 | Canada | Stomachs | 99 | [147] |
| 413 | Rural | 8.4.1 | Western state | 550 | Tennessee | Winter | 1983-1992 | 2.25 | USA | Stomachs | 63 | [12] |
| 414 | Rural | 8.4.2 | Western state | 549 | Tennessee | Autumn | 1983-1992 | 2.25 | USA | Stomachs | 42 | [12] |
| 415 | Urban | 8.2.1 | University of Wisconsin-Madison Arboretum | 673 | Wisconsin | Winter | 2014 | 0.25 | USA | Scats | 24 | [148] |
| 416 | Wilderness | 6.2.10 | Black Hills | 535 | South Dakota | Annual | 1998 | 1 | USA | Scats | 100 | [149] |
| 417 | Rural | 9.4.3 | Pinyon Canyon | 260 | Colorado | Spring | 1983 | 0.25 | USA | Scats | 119 | [150, 151] |
| 418 | Rural | 9.4.3 | Pinyon Canyon | 261 | Colorado | Summer | 1983 | 0.25 | USA | Scats | 76 | [150, 151] |
| 419 | Rural | 9.4.3 | Pinyon Canyon | 259 | Colorado | Autumn | 1983 | 0.25 | USA | Scats | 116 | [150, 151] |
| 420 | Rural | 9.4.3 | Pinyon Canyon | 265 | Colorado | Winter | 1984 | 0.25 | USA | Scats | 320 | [150, 151] |
| 421 | Rural | 9.4.3 | Pinyon Canyon | 263 | Colorado | Spring | 1984 | 0.25 | USA | Scats | 221 | [150, 151] |
| 422 | Rural | 9.4.3 | Pinyon Canyon | 264 | Colorado | Summer | 1984 | 0.25 | USA | Scats | 238 | [150, 151] |
| 423 | Rural | 9.4.3 | Pinyon Canyon | 262 | Colorado | Autumn | 1984 | 0.25 | USA | Scats | 120 | [150, 151] |
| 424 | Rural | 9.4.3 | Pinyon Canyon | 269 | Colorado | Winter | 1985 | 0.25 | USA | Scats | 137 | [150, 151] |
| 425 | Rural | 9.4.3 | Pinyon Canyon | 267 | Colorado | Spring | 1985 | 0.25 | USA | Scats | 301 | [150, 151] |
| 426 | Rural | 9.4.3 | Pinyon Canyon | 268 | Colorado | Summer | 1985 | 0.25 | USA | Scats | 255 | [150, 151] |
| 427 | Rural | 9.4.3 | Pinyon Canyon | 266 | Colorado | Autumn | 1985 | 0.25 | USA | Scats | 421 | [150, 151] |
| 429 | Rural | 9.4.3 | Pinyon Canyon | 270 | Colorado | Spring | 1986 | 0.25 | USA | Scats | 189 | [150, 151] |
| 430 | Rural | 9.4.1 | Statewide | 341 | Kansas | Annual | 1948 | 1 | USA | Stomachs | 177 | [76, 152] |
| 431 | Rural | 9.4.1 | Statewide | 343 | Kansas | Annual | 1949 | 1 | USA | Stomachs | 177 | [76, 152] |
| 432 | Rural | 9.4.1 | Statewide | 345 | Kansas | Annual | 1950 | 1 | USA | Stomachs | 177 | [76, 152] |
| 433 | Rural | 9.4.1 | Statewide | 347 | Kansas | Annual | 1951 | 1 | USA | Stomachs | 177 | [76, 152] |
| 434 | Rural | 9.4.1 | Statewide | 349 | Kansas | Annual | 1952 | 1 | USA | Stomachs | 177 | [76, 152] |
| 435 | Rural | 9.4.1 | Statewide | 351 | Kansas | Annual | 1953 | 1 | USA | Stomachs | 177 | [76, 152] |
| 436 | Rural | 9.4.1 | Statewide | 353 | Kansas | Annual | 1954 | 1 | USA | Stomachs | 177 | [76, 152] |
| 437 | Rural | 9.4.1 | Statewide | 354 | Kansas | Annual | 1955 | 1 | USA | Stomachs | 177 | [76, 152] |
| 438 | Rural | 9.4.1 | Statewide | 355 | Kansas | Annual | 1956 | 1 | USA | Stomachs | 177 | [76, 152] |
| 439 | Rural | 9.4.1 | Statewide | 356 | Kansas | Annual | 1957 | 1 | USA | Stomachs | 177 | [76, 152] |
| 440 | Rural | 9.4.1 | Statewide | 357 | Kansas | Annual | 1958-1962 | 5 | USA | Stomachs | 177 | [76, 152] |
| 441 | Rural | 6.2.14 | Valles Caldera National Preserve | 471 | New Mexico | Annual | 2005-2008 | 4 | USA | Scats | 1385 | [153] |
| 442 | Rural |  | Statewide | 198 | Arkansas | Annual | 1969-1974 | 6 | USA | Scats | 168 | [153] |
| 443 | Rural |  | Statewide | 620 | Texas | Annual | 1994-1997 | 4 | USA | Stomachs | 208 | [154] |
| 444 | Rural | 5.3.1 | North-west | 276 | Connecticut | Spring | 1975 | 0.333 | USA | Scats | 55 | [155] |
| 445 | Rural | 12.1.2 | Laguna de Santiaguillo | 114 | Durango | Annual | 2007 | 1 | Mexico | Scats | 1023 | [156] |
| 446 | Rural | 10.2.4 | San Ignacio | 119 | Durango | Summer | 2000-2001 | 0.5 | Mexico | Scats | 50 | [157] |
| 447 | Rural | 10.2.4 | La Soledad | 113 | Durango | Summer | 2000-2001 | 0.5 | Mexico | Scats | 83 | [157] |
| 448 | Rural | 10.2.1 | Boulder Dam | 438 | Nevada | Annual | 1938-1943 | 6 | USA | Scats | 176 | [158] |
| 449 | Rural | 14.5.2 | Cabo Corrientes | 122 | Jalisco | Winter | 1999-2001 | 1.5 | Mexico | Scats | 69 | [159] |
| 450 | Rural | 14.5.2 | Cabo Corrientes | 121 | Jalisco | Summer | 1999-2001 | 1.5 | Mexico | Scats | 155 | [159] |
| 451 | Rural | 8.3.7 | Statewide | 365 | Louisiana | Annual | 1975-1978 | 4 | USA | Both | 1239 | [160] |
| 452 | Rural | 5.3.1 | Adirondack Mountains | 472 | New York | Annual | 1956-1961 | 6 | USA | Scats | 1500 | [161] |
| 453 | Rural | 8.1.8 | Eastern | 373 | Maine | Summer | 1981 | 0.25 | USA | Scats | 171 | [162] |
| 454 | Rural | 8.1.8 | Eastern | 372 | Maine | Autumn | 1981 | 0.25 | USA | Scats | 251 | [162] |
| 455 | Rural | 6.2.12 | Sagehen Creek basin | 237 | California | Winter | 1966-1969 | 1 | USA | Scats | 65 | [163, 164] |
| 456 | Rural | 6.2.12 | Sagehen Creek basin | 235 | California | Spring | 1966-1969 | 1 | USA | Scats | 30 | [163, 164] |
| 457 | Rural | 6.2.12 | Sagehen Creek basin | 236 | California | Summer | 1966-1969 | 1 | USA | Scats | 59 | [163, 164] |
| 458 | Rural | 9.5.1 | Southern | 616 | Texas | Spring | 1994 | 0.25 | USA | Scats | 200 | [165] |
| 459 | Rural | 9.5.1 | Southern | 617 | Texas | Summer | 1995 | 0.25 | USA | Scats | 98 | [165] |
| 460 | Rural | 9.5.1 | Southern | 618 | Texas | Autumn | 1997 | 0.25 | USA | Scats | 42 | [165] |
| 461 | Rural | 9.5.1 | Southern | 619 | Texas | Winter | 1998 | 0.25 | USA | Scats | 67 | [165] |
| 462 | Rural | 10.2.4 | Bolson de Mapimi | 104 | Chihuahua | Annual | 1985-1986 | 2 | Mexico | Scats | 508 | [166] |
| 463 | Wilderness | 10.2.2 | Sonoran Desert | 147 | Sonora | Annual | 1982 | 1 | Mexico | Scats | 223 | [167] |
| 464 | Wilderness | 10.1.7 | Sevilleta National Wildlife Refuge | 448 | New Mexico | Winter | 1992 | 0.25 | USA | Scats | 30 | [168] |
| 465 | Wilderness | 10.1.7 | Sevilleta National Wildlife Refuge | 446 | New Mexico | Spring | 1992 | 0.25 | USA | Scats | 33 | [168] |
| 466 | Wilderness | 10.1.7 | Sevilleta National Wildlife Refuge | 447 | New Mexico | Summer | 1992 | 0.25 | USA | Scats | 20 | [168] |
| 467 | Wilderness | 10.1.7 | Sevilleta National Wildlife Refuge | 445 | New Mexico | Autumn | 1992 | 0.25 | USA | Scats | 75 | [168] |
| 468 | Wilderness | 10.1.7 | Sevilleta National Wildlife Refuge | 451 | New Mexico | Winter | 1993 | 0.25 | USA | Scats | 45 | [168] |
| 469 | Wilderness | 10.1.7 | Sevilleta National Wildlife Refuge | 450 | New Mexico | Spring | 1993 | 0.25 | USA | Scats | 28 | [168] |
| 470 | Wilderness | 10.1.7 | Sevilleta National Wildlife Refuge | 449 | New Mexico | Autumn | 1993 | 0.25 | USA | Scats | 35 | [168] |
| 471 | Wilderness | 10.1.7 | Sevilleta National Wildlife Refuge | 453 | New Mexico | Winter | 1994 | 0.25 | USA | Scats | 132 | [168] |
| 472 | Wilderness | 10.1.7 | Sevilleta National Wildlife Refuge | 452 | New Mexico | Spring | 1994 | 0.25 | USA | Scats | 20 | [168] |
| 473 | Rural | 13.4.2 | Popocatepetl | 144 | Puebla | Annual | 2001-2003 | 3 | Mexico | Scats | 130 | [169] |
| 474 | Rural | 13.4.2 | Iztaccihuatl | 137 | Puebla | Annual | 2001-2003 | 3 | Mexico | Scats | 115 | [169] |
| 475 | Rural | 13.4.2 | Tlaloc | 145 | Puebla | Annual | 2001-2003 | 3 | Mexico | Scats | 37 | [169] |
| 476 | Rural | 14.5.2 | Chamela-Cuixmala Biosphere Reserve | 123 | Jalisco | Annual | 1996-1997 | 2 | Mexico | Scats | 59 | [170, 171] |
| 477 | Rural | 5.3.1 | Northern state | 383 | Maine | Annual | 1974-1975 | 2 | USA | Stomachs | 80 | [172] |
| 478 | Wilderness | 5.3.1 | North-west | 385 | Maine | Winter | 1974-1975 | 0.4 | USA | Both | 138 | [173] |
| 479 | Wilderness | 5.3.1 | North-west | 384 | Maine | Summer | 1974-1975 | 0.4 | USA | Both | 82 | [173] |
| 480 | Rural | 8.5.1 | Eastern | 488 | North Carolina | Annual | 2009-2011 | 3 | USA | Scats | 554 | [174] |
| 481 | Rural | 8.3.5 | Upper coastal plain | 155 | Alabama | Annual | 1988-1989 | 2 | USA | Scats | 292 | [175] |
| 482 | Rural | 9.4.2 | Wichita Mountains National Wildlife Refuge | 508 | Oklahoma | Annual | 1975-1976 | 1 | USA | Scats | 671 | [176] |
| 483 | Rural | 9.2.3 | De Soto National Wildlife Refuge | 429 | Nebraska | Annual | 1994-1995 | 1 | USA | Scats | 490 | [177] |
| 484 | Rural | 5.2.1 | Chequamegon National Forest | 667 | Wisconsin | Winter | 1975 | 0.25 | USA | Scats | 27 | [178] |
| 485 | Rural | 5.2.1 | Chequamegon National Forest | 668 | Wisconsin | Winter | 1976 | 0.25 | USA | Scats | 92 | [178] |
| 486 | Rural | 5.2.1 | Chequamegon National Forest | 669 | Wisconsin | Winter | 1977 | 0.25 | USA | Scats | 37 | [178] |
| 487 | Wilderness | 10.2.1 | Grand Wash Cliffs, Grand Canyon - North | 179 | Arizona | Spring | 1975 | 0.083333333 | USA | Scats | 112 | [179] |
| 488 | Wilderness | 10.2.1 | Grand Wash Cliffs, Grand Canyon - South | 180 | Arizona | Spring | 1975 | 0.083333333 | USA | Scats | 112 | [179] |
| 489 | Rural | 9.4.2 | Fort Riley Military Reservation | 327 | Kansas | Spring | 1996-1998 | 0.75 | USA | Scats | 48 | [180-182] |
| 490 | Rural | 9.4.2 | Fort Riley Military Reservation | 328 | Kansas | Summer | 1996-1998 | 0.75 | USA | Scats | 153 | [180-182] |
| 491 | Rural | 9.4.2 | Fort Riley Military Reservation | 326 | Kansas | Autumn | 1996-1998 | 0.75 | USA | Scats | 176 | [180-182] |
| 492 | Rural | 9.4.2 | Fort Riley Military Reservation | 329 | Kansas | Winter | 1996-1998 | 0.75 | USA | Scats | 238 | [180-182] |
| 493 | Rural | 9.4.1 | Rita Blanca National Grasslands | 605 | Texas | Annual | 1998-2000 | 2 | USA | Scats | 482 | [183, 184] |
| 494 | Rural | 9.4.1 | Northwest Texas | 576 | Texas | Winter | 1999 | 0.25 | USA | Scats | 71 | [183, 185] |
| 495 | Rural | 9.4.1 | Northwest Texas | 578 | Texas | Spring | 2000 | 0.25 | USA | Scats | 35 | [183, 185] |
| 496 | Rural | 9.4.1 | Northwest Texas | 579 | Texas | Summer | 2000 | 0.25 | USA | Scats | 68 | [183, 185] |
| 497 | Rural | 9.4.1 | Northwest Texas | 577 | Texas | Autumn | 2000 | 0.25 | USA | Scats | 106 | [183, 185] |
| 498 | Rural | 8.3.4 | B.F. Grant Wildlife Management Area | 285 | Georgia | Annual | 2010 | 1 | USA | Scats | 207 | [186] |
| 499 | Rural | 8.3.4 | Cedar Creek Wildlife Management Area | 290 | Georgia | Annual | 2010 | 1 | USA | Scats | 146 | [186] |
| 500 | Rural | 9.4.3 | Pinyon Canyon | 271 | Colorado | Annual | 1997-1998 | 1 | USA | Scats | 469 | [187, 188] |
| 501 | Rural | 9.5.1 | South-east | 612 | Texas | Spring | 1961 | 0.25 | USA | Scats | 618 | [189] |
| 502 | Rural | 9.5.1 | South-east | 613 | Texas | Summer | 1961 | 0.25 | USA | Scats | 582 | [189] |
| 503 | Rural | 9.5.1 | South-east | 611 | Texas | Autumn | 1961 | 0.25 | USA | Scats | 469 | [189] |
| 504 | Rural | 9.5.1 | South-east | 615 | Texas | Winter | 1962 | 0.25 | USA | Scats | 367 | [189] |
| 505 | Rural | 9.5.1 | South-east | 614 | Texas | Spring | 1962 | 0.25 | USA | Scats | 367 | [189] |
| 506 | Wilderness | 6.2.15 | Big Creek | 297 | Idaho | Summer | 1980-1985 | 1.5 | USA | Scats | 30 | [190] |
| 507 | Wilderness | 6.2.15 | Big Creek | 298 | Idaho | Winter | 1980-1985 | 1.5 | USA | Scats | 143 | [190] |
| 508 | Rural | 11.1.2 | Lemoore Naval Airforce Base | 218 | California | Annual | 1992-1993 | 1 | USA | Scats | 97 | [191] |
| 509 | Rural | 8.1.9 | Kings County | 51 | New Brunswick | Annual | 1979-1982 | 1.5 | Canada | Stomachs | 128 | [192] |
| 510 | Rural | 5.4.1 | Athabaska River | 1 | Alberta | Spring | 2006-2008 | 0.5 | Canada | Scats | 67 | [193, 194] |
| 511 | Rural | 8.4.1 | Statewide | 546 | Tennessee | Annual | 1981-1984 | 4 | USA | Stomachs | 262 | [195] |
| 512 | Rural | 9.3.3 | Billings County | 498 | North Dakota | Annual | 1985 | 1 | USA | Scats | 152 | [196] |
| 513 | Rural | 10.2.4 | Janos-Casas Grandes | 105 | Chihuahua | Annual | 1994-1996 | 2 | Mexico | Scats | 76 | [197] |
| 514 | Rural | 9.4.2 | Wichita Mountains National Wildlife Refuge | 509 | Oklahoma | Annual | 1976-1977 | 1 | USA | Scats | 253 | [198] |
| 515 | Rural | 8.1.8 | Eastern | 376 | Maine | Spring | 1979-1984 | 1.5 | USA | Scats | 279 | [199] |
| 516 | Rural | 8.1.8 | Eastern | 377 | Maine | Summer | 1979-1984 | 1.5 | USA | Scats | 460 | [199] |
| 517 | Rural | 8.1.8 | Eastern | 375 | Maine | Autumn | 1979-1984 | 1.5 | USA | Scats | 317 | [199] |
| 518 | Rural | 8.1.8 | Eastern | 378 | Maine | Winter | 1979-1984 | 1.5 | USA | Scats | 93 | [199] |
| 519 | Rural | 9.6.1 | San Jose | 130 | Nuevo Leon | Spring | 1997-1998 | 0.25 | Mexico | Scats | 159 | [32, 200] |
| 520 | Rural | 9.6.1 | San Jose | 131 | Nuevo Leon | Summer | 1997-1998 | 0.25 | Mexico | Scats | 269 | [32, 200] |
| 521 | Rural | 9.6.1 | San Jose | 129 | Nuevo Leon | Autumn | 1997-1998 | 0.25 | Mexico | Scats | 488 | [32, 200] |
| 522 | Rural | 9.6.1 | San Jose | 132 | Nuevo Leon | Winter | 1997-1998 | 0.25 | Mexico | Scats | 201 | [32, 200] |
| 523 | Rural | 10.2.4 | Rancho el Macho | 107 | Coahuila | Winter | 1991-992 | 0.25 | Mexico | Scats | 56 | [201] |
| 524 | Rural | 8.3.5 | Central | 403 | Mississippi | Annual | 1993-1994 | 1 | USA | Scats | 82 | [202] |
| 525 | Urban | 9.2.1 | Calgary | 4 | Alberta | Annual | 2006-2007 | 1 | Canada | Scats | 484 | [203, 204] |
| 526 | Wilderness | 6.2.14 | Rocky Mountain National Park | 275 | Colorado | Winter | 1979-1980 | 0.5 | USA | Scats | 61 | [205] |
| 527 | Suburban | 8.5.3 | Brooker Creek Preserve | 278 | Florida | Summer | 2005-2007 | 1.5 | USA | Scats | 27 | [206] |
| 528 | Suburban | 8.5.3 | Brooker Creek Preserve | 279 | Florida | Winter | 2005-2007 | 1.5 | USA | Scats | 22 | [206] |
| 529 | Suburban | 8.5.3 | Pinellas County | 283 | Florida | Summer | 2005-2007 | 1.5 | USA | Scats | 53 | [206] |
| 530 | Rural | 13.4.2 | Izta-Popo National Park | 138 | Puebla | Annual | 2004-2005 | 1 | Mexico | Scats | 70 | [207] |
| 531 | Rural | 13.4.2 | Parque Nacionale Pico de Orizaba | 143 | Puebla | Annual | 2000-2001 | 1 | Mexico | Scats | 136 | [208] |
| 532 | Rural | 9.2.3 | South-western | 324 | Iowa | Summer | 1970-1972 | 1.5 | USA | Scats | 147 | [209] |
| 533 | Rural | 5.1.4 | Maritime Barrens | 59 | Newfoundland | Summer | 2009 | 0.33 | Canada | Scats | 84 | [210] |
| 534 | Rural | 13.1.1 | Mazatzal Mountains | 181 | Arizona | Annual | 2000 | 1 | USA | Scats | 91 | [211] |
| 535 | Rural | 13.1.1 | Mazatzal Mountains | 182 | Arizona | Annual | 2001 | 1 | USA | Scats | 174 | [211] |
| 536 | Rural | 13.1.1 | Mazatzal Mountains | 183 | Arizona | Annual | 2002 | 1 | USA | Scats | 237 | [211] |
| 537 | Rural | 13.1.1 | Mazatzal Mountains | 184 | Arizona | Annual | 2003 | 1 | USA | Scats | 161 | [211] |
| 538 | Rural |  | Statewide | 256 | California | Annual | 1932-1933 | 1 | USA | Stomachs | 240 | [212] |
| 539 | Rural | 8.5.1 | Red Wolf Experimental Area | 497 | North Carolina | Winter | 2009-2010 | 0.25 | USA | Scats | 64 | [213] |
| 540 | Rural | 9.4.3 | Rolling Plains bioregion | 606 | Texas | Annual | 1971-1972 | 1 | USA | Scats | 223 | [214] |
| 541 | Rural | 9.4.3 | Rolling Plains bioregion | 607 | Texas | Annual | 1972-1973 | 1 | USA | Scats | 291 | [214] |
| 542 | Rural | 5.3.1 | Eastern Townships region | 72 | Quebec | Annual | 1976-1979 | 4 | Canada | Scats | 860 | [215, 216] |
| 543 | Rural | 8.3.7 | North-western uplands | 364 | Louisiana | Annual |  | 1 | USA | Scats | 130 | [217] |
| 544 | Rural | 13.4.2 | La Comunidad Indígena de Nuevo San Juan Parangaricutiro | 140 | Michoacan | Spring | 1998 | 0.25 | Mexico | Scats | 207 | [218] |
| 545 | Rural | 13.4.2 | La Comunidad Indígena de Nuevo San Juan Parangaricutiro | 141 | Michoacan | Summer | 1998 | 0.25 | Mexico | Scats | 207 | [218] |
| 546 | Rural | 13.4.2 | La Comunidad Indígena de Nuevo San Juan Parangaricutiro | 139 | Michoacan | Autumn | 1998 | 0.25 | Mexico | Scats | 208 | [218] |
| 547 | Rural | 13.4.2 | La Comunidad Indígena de Nuevo San Juan Parangaricutiro | 142 | Micoacan | Winter | 1998 | 0.25 | Mexico | Scats | 208 | [218] |
| 548 | Rural |  | Region | 58 | New Brunswick - Nova Scotia | Annual | 1979-1981 | 3 | Canada | Stomachs | 265 | [219] |
| 549 | Rural | 8.1.1 | Prince Edward County | 68 | Ontario | Winter | 1967-1969 | 0.75 | Canada | Stomachs | 42 | [219] |
| 550 | Wilderness | 6.2.10 | Jackson Hole | 675 | Wyoming | Annual | 1927-1930 | 3 | USA | Scats | 714 | [220] |
| 551 | Wilderness | 6.2.10 | Yellowstone National Park | 688 | Wyoming | Annual | 1937-39 | 3 | USA | Scats |  | [221] |
| 552 | Wilderness | 6.2.1 | St. George region | 41 | British Columbia | Annual | 1933-1934 | 2 | Canada | Scats | 311 | [222] |
| 553 | Rural | 10.2.2 | San Carlos Reservation | 190 | Arizona | Annual | 1943-1944 | 2 | USA | Scats | 3981 | [223] |
| 554 | Urban | 9.2.1 | Edmonton | 8 | Alberta | Annual | 2009-2012 | 4 | Canada | Scats | 531 | [224, 225] |
| 555 | Urban | 9.2.1 | Calgary | 5 | Alberta | Annual | 2006-2007 | 2 | Canada | Scats | 484 | [224, 225] |
| 556 | Rural | 9.2.1 | Elk Island National Park | 9 | Alberta | Annual | 1994-2000 | 7 | Canada | Scats | 1224 | [224, 225] |
| 557 | Rural | 5.4.1 | Wabasca | 38 | Alberta | Annual | 2005-2007 | 3 | Canada | Scats | 120 | [224, 225] |
| 558 | Rural | 7.1.8 | Hopland Experimental Research Area | 207 | California | Summer | 1994 | 0.25 | USA | Scats | 113 | [226-228] |
| 559 | Rural | 7.1.8 | Hopland Experimental Research Area | 206 | California | Autumn | 1994 | 0.25 | USA | Scats | 123 | [226-228] |
| 560 | Rural | 7.1.8 | Hopland Experimental Research Area | 209 | California | Winter | 1995 | 0.25 | USA | Scats | 80 | [226-228] |
| 561 | Rural | 7.1.8 | Hopland Experimental Research Area | 208 | California | Spring | 1995 | 0.25 | USA | Scats | 103 | [226-228] |
| 562 | Rural | 8.1.8 | Mt Desert Island | 382 | Maine | Winter | 1989-1991 | 0.75 | USA | Scats | 76 | [229] |
| 563 | Rural | 8.1.8 | Mt Desert Island | 380 | Maine | Spring | 1989-1991 | 0.75 | USA | Scats | 175 | [229] |
| 564 | Rural | 8.1.8 | Mt Desert Island | 381 | Maine | Summer | 1989-1991 | 0.75 | USA | Scats | 250 | [229] |
| 566 | Rural | 12.1.1 | Appleton-Whittell Research Ranch | 174 | Arizona | Spring | 1982 | 0.25 | USA | Scats | 215 | [230] |
| 567 | Rural | 12.1.1 | Appleton-Whittell Research Ranch | 175 | Arizona | Summer | 1982 | 0.25 | USA | Scats | 59 | [230] |
| 568 | Rural | 12.1.1 | Appleton-Whittell Research Ranch | 173 | Arizona | Autumn | 1982 | 0.25 | USA | Scats | 210 | [230] |
| 569 | Rural | 12.1.1 | Appleton-Whittell Research Ranch | 177 | Arizona | Winter | 1983 | 0.25 | USA | Scats | 200 | [230] |
| 570 | Rural | 12.1.1 | Appleton-Whittell Research Ranch | 176 | Arizona | Spring | 1983 | 0.25 | USA | Scats | 75 | [230] |
| 571 | Rural | 8.4.1 | Berry College Campus | 286 | Georgia | Annual | 2005-2006 | 1 | USA | Scats | 127 | [231] |
| 572 | Rural | 5.2.1 | Beaver Island | 392 | Michigan | Winter | 1956-1965 | 2.5 | USA | Scats | 48 | [232] |
| 573 | Rural | 5.2.1 | Shingleton | 393 | Michigan | Winter | 1956-1965 | 2.5 | USA | Scats | 44 | [232] |
| 574 | Rural | 9.3.3 | South-west | 537 | South Dakota | Summer | 1998 | 0.583333333 | USA | Scats | 25 | [233] |
| 575 | Rural | 9.3.3 | North-west | 536 | South Dakota | Summer | 1998 | 0.583333333 | USA | Scats | 25 | [233] |
| 576 | Wilderness | 5.3.1 | Northern | 55 | New Brunswick | Spring | 1983 | 0.25 | Canada | Scats | 53 | [234] |
| 577 | Wilderness | 5.3.1 | Northern | 56 | New Brunswick | Summer | 1983 | 0.25 | Canada | Scats | 114 | [234] |
| 578 | Wilderness | 5.3.1 | Northern | 54 | New Brunswick | Autumn | 1983 | 0.25 | Canada | Scats | 61 | [234] |
| 579 | Wilderness | 5.3.1 | Northern | 57 | New Brunswick | Winter | 1984 | 0.25 | Canada | Scats | 115 | [234] |
| 580 | Suburban | 8.3.6 | Western | 548 | Tennessee | Annual | 1997-1998 | 1 | USA | Scats | 675 | [235] |
| 581 | Rural | 10.1.6 | Blue Mountains | 635 | Utah | Annual | 1977-1979 | 3 | USA | Scats | 318 | [236] |
| 581 | Rural | 10.1.6 | Elk Ridge | 647 | Utah | Annual | 1977-1979 | 3 | USA | Scats | 142 | [236] |
| 582 | Rural | 6.2.12 | Lassen Peak | 212 | California | Annual | 1998-2002 | 4 | USA | Scats | 58 | [237] |
| 583 | Rural | 8.1.1 | Champlain Valley | 654 | Vermont | Winter | 1984-1988 | 1 | USA | Scats | 90 | [238] |
| 584 | Rural | 8.1.1 | Champlain Valley | 652 | Vermont | Spring | 1984-1988 | 1 | USA | Scats | 100 | [238] |
| 585 | Rural | 8.1.1 | Champlain Valley | 653 | Vermont | Summer | 1984-1988 | 1 | USA | Scats | 59 | [238] |
| 586 | Rural | 8.1.1 | Champlain Valley | 651 | Vermont | Autumn | 1984-1988 | 1 | USA | Scats | 57 | [238] |
| 587 | Rural | 8.3.2 | Clay County | 313 | Illinois | Winter | 1977-1978 | 0.25 | USA | Stomachs | 89 | [239] |
| 588 | Rural | 9.4.1 | East-central | 444 | New Mexico | Annual | 1977-1978 | 1 | USA | Stomachs | 84 | [240] |
| 589 | Urban | 7.1.7 | Snohomish | 658 | Washington | Annual | 1989-1990 | 1 | USA | Scats |  | [241] |
| 590 | Rural | 9.4.1 | Rocky Flats Nuclear Weapons Plant | 272 | Colorado | Summer | 1976 | 0.33 | USA | Scats | 54 | [242] |
| 591 | Rural | 5.3.1 | Eastern | 374 | Maine | Annual | 1968-1973 | 6 | USA | Stomachs | 51 | [173] |
| 592 | Rural | 10.2.4 | Statewide | 455 | New Mexico | Annual | 1964 | 1 | USA | Stomachs | 52 | [243] |
| 593 | Rural | 13.4.2 | Michoacan estate | 127 | Michoacan | Annual | 1984-1986 | 3 | Mexico | Scats | 170 | [244] |
| 594 | Rural | 5.3.1 | Chic-Chocs Game Reserve | 70 | Quebec | Summer | 1988 | 0.25 | Canada | Scats | 231 | [245] |
| 595 | Rural | 5.3.1 | Chic-Chocs Game Reserve | 71 | Quebec | Summer | 1991 | 0.25 | Canada | Scats | 435 | [245] |
| 596 | Rural | 10.2.3 | Vizcaino Desert | 100 | Baja California Sur | Winter | 1984 | 0.25 | Mexico | Stomachs | 34 | [246] |
| 597 | Rural | 13.1.1 | South-west | 454 | New Mexico | Annual | 1973 | 1 | USA | Scats | 546 | [240] |
| 598 | Rural | 14.5.2 | Tenacatita | 125 | Jalisco | Annual | 1994-1995 | 1 | Mexico | Scats | 115 | [240] |
| 599 | Suburban | 8.3.4 | Lee County | 152 | Alabama | Annual | Undated | 1 | USA | Scats | 159 | [247] |
| 600 | Rural | 8.3.5 | Pinebloom | 296 | Georgia | Summer | 2001-2002 | 0.5 | USA | Stomachs | 22 | [248] |
| 601 | Rural | 8.3.4 | National Environmental Research Park | 528 | South Carolina | Summer | 2005 | 0.25 | USA | Scats | 136 | [249, 250] |
| 602 | Rural | 8.3.4 | National Environmental Research Park | 527 | South Carolina | Spring | 2005 | 0.25 | USA | Scats | 84 | [249, 250] |
| 603 | Rural | 8.3.4 | National Environmental Research Park | 531 | South Carolina | Winter | 2006 | 0.25 | USA | Scats | 70 | [249, 250] |
| 604 | Rural | 8.3.4 | National Environmental Research Park | 529 | South Carolina | Spring | 2006 | 0.25 | USA | Scats | 82 | [249, 250] |
| 605 | Rural | 8.3.4 | National Environmental Research Park | 530 | South Carolina | Summer | 2006 | 0.25 | USA | Scats | 40 | [249, 250] |
| 606 | Rural | 13.2.1 | Reserva Biosfera de la Michilia | 116 | Durango | Spring | 1986 | 0.25 | Mexico | Scats | 70 | [251] |
| 607 | Rural | 13.2.1 | Reserva Biosfera de la Michilia | 117 | Durango | Summer | 1986 | 0.25 | Mexico | Scats | 62 | [251] |
| 608 | Rural | 13.2.1 | Reserva Biosfera de la Michilia | 115 | Durango | Autumn | 1986 | 0.25 | Mexico | Scats | 54 | [251] |
| 609 | Rural | 13.2.1 | Reserva Biosfera de la Michilia | 118 | Durango | Winter | 1987 | 0.25 | Mexico | Scats | 144 | [251] |
| 610 | Wilderness | 6.2.8 | Lava Beds National Monument | 214 | California | Spring | 1983 | 0.25 | USA | Scats | 80 | [252] |
| 611 | Wilderness | 6.2.8 | Lava Beds National Monument | 215 | California | Summer | 1983 | 0.25 | USA | Scats | 68 | [252] |
| 612 | Wilderness | 6.2.8 | Lava Beds National Monument | 213 | California | Autumn | 1983 | 0.25 | USA | Scats | 72 | [252] |
| 613 | Wilderness | 6.2.8 | Lava Beds National Monument | 216 | California | Winter | 1983 | 0.25 | USA | Scats | 75 | [252] |
| 614 | Rural | 12.1.1 | Santa Rita Experimental Reserve | 195 | Arizona | Winter | 1975 | 0.25 | USA | Scats | 283 | [253] |
| 615 | Rural | 12.1.1 | Santa Rita Experimental Reserve | 193 | Arizona | Spring | 1975 | 0.25 | USA | Scats | 360 | [253] |
| 616 | Rural | 12.1.1 | Santa Rita Experimental Reserve | 194 | Arizona | Summer | 1975 | 0.25 | USA | Scats | 99 | [253] |
| 617 | Rural | 12.1.1 | Santa Rita Experimental Reserve | 192 | Arizona | Autumn | 1975 | 0.25 | USA | Scats | 218 | [253] |
| 618 | Rural | 10.1.7 | Spider Ranch | 196 | Arizona | Spring | 1970 | 0.25 | USA | Scats | 113 | [254] |
| 619 | Rural | 10.1.7 | Spider Ranch | 197 | Arizona | Summer | 1970 | 0.25 | USA | Scats | 109 | [254] |
| 620 | Rural | 6.1.12 | Sierra National Forest | 252 | California | Spring | 1986 | 0.25 | USA | Scats | 60 | [255] |
| 621 | Rural | 6.1.12 | Sierra National Forest | 253 | California | Summer | 1986 | 0.25 | USA | Scats | 216 | [255] |
| 622 | Rural | 6.1.12 | Sierra National Forest | 251 | California | Autumn | 1986 | 0.25 | USA | Scats | 199 | [255] |
| 623 | Rural | 8.3.6 | Western | 547 | Tennessee | Annual | 1979-1981 | 3 | USA | Stomachs | 54 | [256] |
| 624 | Rural | 9.5.1 | Padre Island | 601 | Texas | Summer | 1995 | 0.25 | USA | Scats | 75 | [257] |
| 625 | Rural | 9.5.1 | Padre Island | 600 | Texas | Autumn | 1995 | 0.25 | USA | Scats | 42 | [257] |
| 626 | Rural | 9.5.1 | Padre Island | 603 | Texas | Winter | 1996 | 0.25 | USA | Scats | 97 | [257] |
| 627 | Rural | 9.5.1 | Padre Island | 602 | Texas | Spring | 1996 | 0.25 | USA | Scats | 64 | [257] |
| 628 | Rural | 9.5.1 | King Ranch | 585 | Texas | Summer | 1995 | 0.25 | USA | Scats | 144 | [257] |
| 629 | Rural | 9.5.1 | King Ranch | 584 | Texas | Autumn | 1995 | 0.25 | USA | Scats | 73 | [257] |
| 630 | Rural | 9.5.1 | King Ranch | 587 | Texas | Winter | 1996 | 0.25 | USA | Scats | 93 | [257] |
| 631 | Rural | 9.5.1 | King Ranch | 586 | Texas | Spring | 1996 | 0.25 | USA | Scats | 93 | [257] |
| 632 | Rural | 9.5.1 | Matagorda Island | 597 | Texas | Summer | 1995 | 0.25 | USA | Scats | 81 | [257] |
| 633 | Rural | 9.5.1 | Matagorda Island | 596 | Texas | Autumn | 1995 | 0.25 | USA | Scats | 151 | [257] |
| 634 | Rural | 9.5.1 | Matagorda Island | 599 | Texas | Winter | 1996 | 0.25 | USA | Scats | 149 | [257] |
| 635 | Rural | 9.5.1 | Matagorda Island | 598 | Texas | Spring | 1996 | 0.25 | USA | Scats | 146 | [257] |
| 636 | Rural | 9.5.1 | Aransas National Wildlife Refuge | 556 | Texas | Summer | 1995 | 0.25 | USA | Scats | 108 | [257] |
| 637 | Rural | 9.5.1 | Aransas National Wildlife Refuge | 555 | Texas | Autumn | 1995 | 0.25 | USA | Scats | 78 | [257] |
| 638 | Rural | 9.5.1 | Aransas National Wildlife Refuge | 558 | Texas | Winter | 1996 | 0.25 | USA | Scats | 120 | [257] |
| 639 | Rural | 9.5.1 | Aransas National Wildlife Refuge | 557 | Texas | Spring | 1996 | 0.25 | USA | Scats | 89 | [257] |
| 640 | Rural | 8.5.3 | Yawkey Wildlife Centre | 534 | South Carolina | Annual | 2009-2011 | 2 | USA | Scats | 72 | [258] |
| 641 | Rural | 9.4.1 | Sherman County | 340 | Kansas | Annual | 1996 | 0.7 | USA | Scats | 23 | [259] |
| 642 |  |  | Western USA | 427 | Multiple | Autumn | 1931-1932 | 0.5 | USA | Stomachs | 1453 | [260] |
| 643 |  |  | Western USA | 428 | Multiple | Winter | 1931-1934 | 1 | USA | Stomachs | 1697 | [261] |
| 644 |  |  | Statewide | 522 | Pennsylvania | Annual | 2002-2003; 2005-2007 | 3 | USA | Stomachs | 98 | [262] |
| 645 | Rural | 8.5.3 | Eglin Air Force Base | 281 | Florida | Summer | 1994-1996 | 0.75 | USA | Scats | 58 | [263] |
| 646 | Rural | 8.5.3 | Eglin Air Force Base | 280 | Florida | Autumn | 1994-1996 | 0.75 | USA | Scats | 77 | [263] |
| 647 | Rural | 8.5.3 | Eglin Air Force Base | 282 | Florida | Winter | 1994-1996 | 0.75 | USA | Scats | 23 | [263] |
| 648 | Rural | 8.3.5 | Fort Bragg Military Installation | 494 | North Carolina | Summer | 2011-2012 | 0.25 | USA | Scats | 77 | [89] |
| 649 | Rural | 8.3.5 | Fort Bragg Military Installation | 490 | North Carolina | Autumn | 2011-2012 | 0.25 | USA | Scats | 164 | [89] |
| 650 | Rural | 8.3.5 | Fort Bragg Military Installation | 496 | North Carolina | Winter | 2011-2012 | 0.25 | USA | Scats | 40 | [89] |
| 651 | Rural | 8.3.5 | Fort Bragg Military Installation | 492 | North Carolina | Spring | 2011-2012 | 0.25 | USA | Scats | 34 | [89] |
| 652 | Rural | 8.5.3 | Avon Park Air Force Range | 277 | Florida | Annual | 2001-2002 | 1 | USA | Scats | 86 | [264] |
| 653 | Wilderness | 7.1.3 | Kenai National Wildlife Refuge | 160 | Alaska | Annual | 1976-1980 | 5 | USA | Scats | 107 | [265] |
| 654 | Rural | 6.2.7 | Willamette National Forest | 518 | Oregon | Winter | 1982-1984 | 0.75 | USA | Scats | 190 | [266] |
| 655 | Rural | 6.2.7 | Willamette National Forest | 516 | Oregon | Spring | 1982-1984 | 0.75 | USA | Scats | 192 | [266] |
| 656 | Rural | 6.2.7 | Willamette National Forest | 517 | Oregon | Summer | 1982-1984 | 0.75 | USA | Scats | 324 | [266] |
| 657 | Rural | 6.2.7 | Willamette National Forest | 515 | Oregon | Autumn | 1982-1984 | 0.75 | USA | Scats | 128 | [266] |
| 658 | Rural | 13.1.1 | Beaver Ck, Coconino National Forest | 178 | Arizona | Annual | 1972-1974 | 1 | USA | Scats | 55 | [267] |
| 659 | Rural | 8.1.5 | Wabasha County | 402 | Minnesota | Annual | 2001-2003 | 2 | USA | Scats | 66 | [268] |
| 660 | Wilderness | 6.2.14 | Rocky Mountain Biological Laboratory | 273 | Colorado | Spring | 1984-1989 | 1.5 | USA | Scats | 116 | [269] |
| 661 | Wilderness | 6.2.14 | Rocky Mountain Biological Laboratory | 274 | Colorado | Summer | 1984-1989 | 1.5 | USA | Scats | 129 | [269] |
| 662 | Rural | 8.4.1 | Cherokee County | 151 | Alabama | Annual | 2006-2007 | 1 | USA | Scats | 150 | [270] |
| 663 | Rural | 11.1.3 | San Gabriel Mountains | 241 | California | Annual | 1948-1952 | 5 | USA | Scats | 39 | [271] |
| 664 | Rural | 8.3.1 | Huntington Wildlife Forest | 478 | New York | Winter | 2009-2011 | 0.5 | USA | Scats | 74 | [272] |
| 665 | Rural | 8.3.1 | Huntington Wildlife Forest | 477 | New York | Summer | 2009-2011 | 0.5 | USA | Scats | 100 | [272] |
| 666 | Rural | 11.1.3 | Cuyamaca Rancho State Park | 205 | California | Annual | 1983-1984 | 1 | USA | Scats | 114 | [273] |
| 667 | Rural | 8.3.2 | Crab Orchard National Wildlife Refuge | 314 | Illinois | Annual | 1980 | 1 | USA | Scats | 123 | [274] |
| 668 | Rural | 5.3.1 | Statewide | 443 | New Hampshire | Annual | 1972-1975 | 4 | USA | Stomachs | 54 | [275] |
| 669 | Wilderness | 6.2.5 | Olympic National Park | 655 | Washington | Annual | 2005-2006 | 1 | USA | Scats | 260 | [276, 277] |
| 670 | Rural | 7.1.8 | Elliott State Forest | 510 | Oregon | Spring | 2001-2002 | 0.25 | USA | Scats | 100 | [278] |
| 671 | Rural | 7.1.8 | Elliott State Forest | 511 | Oregon | Summer | 2001-2002 | 0.25 | USA | Scats | 113 | [278] |
| 672 | Rural | 7.1.8 | Elliott State Forest | 512 | Oregon | Winter | 2001-2002 | 0.25 | USA | Scats | 96 | [278] |
| 673 | Rural | 8.4.3 | Elk County | 519 | Pennsylvania | Annual | 1991 | 1 | USA | Scats | 63 | [279] |
| 674 | Rural | 8.4.2 | Franklin County | 520 | Pennsylvania | Annual | 1991 | 1 | USA | Scats | 110 | [279] |
| 675 | Rural | 5.3.3 | Wyoming County | 523 | Pennsylvania | Annual | 1991 | 1 | USA | Scats | 31 | [279] |
| 676 | Rural | 5.3.3 | Wyoming County | 524 | Pennsylvania | Annual | 1992 | 1 | USA | Scats | 94 | [279] |
| 677 | Rural | 8.3.5 | Cross border | 407 | Mississippi - Alabama | Annual | 1980-1984 | 5 | USA | Stomachs | 100 | [280] |
| 678 | Rural |  | Regional | 666 | Western USA | Winter | 1919-1928 | 2.5 | USA | Stomachs | 20000 | [281] |
| 679 | Rural |  | Regional | 664 | Western USA | Spring | 1919-1928 | 2.5 | USA | Stomachs | 20000 | [281] |
| 680 | Rural |  | Regional | 665 | Western USA | Summer | 1919-1928 | 2.5 | USA | Stomachs | 20000 | [281] |
| 681 | Rural |  | Regional | 663 | Western USA | Autumn | 1919-1928 | 2.5 | USA | Stomachs | 20000 | [281] |
| 682 | Rural | 9.5.1 | Welder Wildlife Refuge | 625 | Texas | Winter | 1978-1979 | 0.5 | USA | Scats | 679 | [282] |
| 683 | Rural | 9.5.1 | Welder Wildlife Refuge | 623 | Texas | Spring | 1978-1979 | 0.5 | USA | Scats | 679 | [282] |
| 684 | Rural | 9.5.1 | Welder Wildlife Refuge | 624 | Texas | Summer | 1978-1979 | 0.5 | USA | Scats | 679 | [282] |
| 685 | Rural | 9.5.1 | Welder Wildlife Refuge | 622 | Texas | Autumn | 1978-1979 | 0.5 | USA | Scats | 679 | [282] |
| 686 | Rural | 9.5.1 | Welder Wildlife Refuge | 629 | Texas | Winter | 2003-2004 | 0.5 | USA | Scats | 309 | [282] |
| 687 | Rural | 9.5.1 | Welder Wildlife Refuge | 627 | Texas | Spring | 2003-2004 | 0.5 | USA | Scats | 309 | [282] |
| 688 | Rural | 9.5.1 | Welder Wildlife Refuge | 628 | Texas | Summer | 2003-2004 | 0.5 | USA | Scats | 309 | [282] |
| 689 | Rural | 9.5.1 | Welder Wildlife Refuge | 626 | Texas | Autumn | 2003-2004 | 0.5 | USA | Scats | 309 | [282] |
| 689 |  |  | Altiplano | 689 | Zacatecas | Annual | 2007-2008 | 2 | Mexico | Scats | 76 | [283] |
| 690 |  |  | Tensas River NWR | 690 | Louisiana | Annual | 2013-2014 | 2 | USA | Scats | 274 | [284] |
| 999 | Suburban |  | Los Angeles | 223 | California | Wet | 2016-2018 | 3 | USA | Scats | 788 | [45] |

**References**

1. Randa L. A., Cooper, D. M., Meserve, P. L., Yunger, J. A. 2009. Prey switching of sympatric canids in response to variable prey abundance. Journal of Mammalogy 90:594-603.

2. Albers, G. 2012. Coyote diets in West Virginia. Master's thesis, West Virginia University, Morgantown, West Virginia, USA.

3. Boser, C. L 2010. Diet and hunting behavior of Coyotes in agricultural-forest landscapes of New York state. State University of New York, Syracuse, New York, USA.

4. Brown, K. L. 1977. Coyote food habits in relation to a fluctuating prey base in South Texas. Master's Thesis, Texas A&M University, College Station, Texas, USA.

5. Brundige, G.C. 1993. Predation ecology of the eastern coyote, *Canis latrans* var., in the Adirondacks, New York. Dissertation, State University of New York, Syracuse, New York, USA.

6. Martínez Calderas, J.M., 2005. Forrajeo optimo del coyote en la Reserva de la Biosfera de Mapimi. Bachelor's thesis, Universidad Autónoma de Ciudad Juárez, Ciudad Juárez, Chihuahua, México.

7. Clark, F.W. 1972. Influence of jackrabbit density on coyote population change. The Journal of Wildlife Management 36:343-356.

8. Cornely, J.E. 1980. Home ranges and diets of coyotes in Joshua Tree National Monument in relation to prey densities. Cooperative National Park Resources Studies Unit, University of Nevada, Las Vegas, Nevada, USA.

9. Ellis, R.J. 1959. Food habits and control of coyotes in northcentral Oklahoma. Master's thesis, Oklahoma State University, Stillwater, Oklahoma, USA.

10. Etheredge, C. 2013. Ecology and impacts of Coyotes (*Canis latrans*) in the southeastern United States.Dissertation. Clemson University, Clemson, South Carolina, USA.

11. Fitch, H.S. 1948. A study of coyote relationships on cattle range. The Journal of Wildlife Management 12:73-78.

12. Gabor, T.M. 1993. An assessment of the feeding ecology of coyotes in Western Tennessee. Master's thesis, Memphis State University, Memphis, Tennessee, USA.

13. Hamlin K. L., Riley, S.J., Pyrah, D., Dood, A. R., Mackie, R. J. 1984. Relationships among mule deer fawn mortality, coyotes, and alternate prey species during summer. The Journal of Wildlife Management 48: 489-99.

14. Hamlin, K. L., Mackie, R. J. 1989. Mule deer in the Missouri River Breaks, Montana: a study of population dynamics in a fluctuating environment. Montana Department of Fish, Widlife and Parks, Bozeman, Montana, USA.

15. Henke, S. E. 1992. Effect of coyote removal on the faunal community ecology of a short-grass prairie. Dissertation, Texas Tech University, Lubbock, Texas, USA.

16. Henke, S. E., Bryant, F. C. 1999. Effects of coyote removal on the faunal community in western Texas. The Journal of Wildlife Management 63:1066-1081.

17. Hoffman, S.W. 1979. Coyote-prey relationships in Curlew Valley during a period of low jackrabbit density. Master's thesis, Utah State University, Logam Utah, USA.

18. Kauffeld, J. D. 1977. Availability of natural prey and it's relationship to coyote predation on domestic sheep. Master's thesis, University of Nevada, Reno, Nevada, USA.

19. Lingle, S. 2000. Seasonal variation in coyote feeding behaviour and mortality of white-tailed deer and mule deer. Canadian Journal of Zoology 78:85-99.

20. MacDonald, S. A. T. 2002. Coyote food habits and the relative abundance of rodents in San Mateo County. Master's thesis, San Jose State University, San Jose, California, USA.

21. Major, J. T. 1983. Ecology and interspecific relationships of coyotes, bobcats and red foxes in western Maine. Dissertation, University of Maine, Orono, Maine, USA.

22. Major, J. T., Sherburne, J. A. 1987. Interspecific relationships of coyotes, bobcats, and red foxes in western Maine. The Journal of Wildlife Management 51:606-616.

23. Meleshko, D.W. 1986. Feeding habits of sympatric canids in an area of moderate ungulate density. Master's thesis, University of Alberta, Edmonton, Alberta, Canada.

24. Melville, H. I. 2012. The impacts of three common mesopredators on the reintroduced population of eastern wild turkeys in Texas. Dissertation, Texas A&M University, College Station, Texas, USA.

25. O'Donoghue, M., Boutin S., Krebs C. J., Hofer, E. J. 1997. Numerical responses of coyotes and lynx to the snowshoe hare cycle. Oikos 80:150-62.

26. O'Donoghue, M., Boutin S., Krebs, C. J., Murray, D. L., Hofer, E. J. 1998. Behavioural responses of coyotes and lynx to the snowshoe hare cycle. Oikos 82:169-183.

27. Pastuck, R. D. 1974. Some aspects of the ecology of the coyote (*Canis latrans* Say) in southwestern Manitoba. Master's thesis, University of Manitoba, Winnipeg, Manitoba, Canada.

28. Prugh, L. 2004. Foraging ecology of coyotes in the Alaska Range. Dissertation, University of British Columbia, Vancouver, British Columbia, Canada.

29. Prugh, L. R. 2005. Coyote prey selection and community stability during a decline in food supply. Oikos 110:253-264.

30. Prugh, L. R., Arthur, S. M., Ritland, C. E. 2008. Use of faecal genotyping to determine individual diet. Wildlife Biology 14:318-330.

31. Sanyal, N .K. 1975. The effects of grazing regime on coyote-sheep relationships in southwest Texas. Master's thesis, Texas A&M University, College Station, Texas, USA.

32. López Soto, J. H., Badii, M. H. 2000. Depredación en crías de venado cola blanca (*Odocoileus virginianus texanus*) por coyote (*Canis latrans*) en una Unidad de Manejo y Aprovechamiento del norte de Nuevo León, México. Acta Zoológica Mexicana 81:135-138.

33. Staples III, W. R. 1995. Lynx and coyote diet and habitat relationships during a low hare population on the Kenai Peninsula, Alaska. Master's thesis, University of Alaska, Fairbanks, Alaska, USA.

34. Stoel, P. 1977. Some coyote food habit patterns in shrub-steppe habitat in south-central Washington. Master's thesis, Portland State University, Portland, Oregon, USA.

35. Todd, M. A. 1987. The foraging behaviour of a coyote population on the Long Point National Wildlife Area, Southern Ontario. Master's thesis, University of Waterloo, Waterloo, Ontario, Canada.

36. Todd, A. W., Keith, L. B., Fischer, C. A. 1981. Population ecology of coyotes during a fluctuation of snowshoe hares. The Journal of Wildlife Management 45:629-640.

37. Weaver, J. L. 1977. Coyote-food base relationships in Jackson Hole, Wyoming. Master's thesis, Utah State University, Logan, Utah, USA.

38. Tyson, M. 2012. Diets of coyotes on the Rolling Plains Quail Research Ranch, Texas.master's thesis, Texas Tech University, Lubbock, Texas, USA.

39. Blanton, K. M. 1988. Summer diet of coyotes in the Southeast, and the response of coyotes to siren surveys. Master's thesis, Mississippi State University, Mississippi State, Mississippi, USA.

40. Brillhart, D., Kaufman, D. 1994.Temporal variation in coyote prey in tallgrass prairie of eastern Kansas. Prairie Naturalist 26:93.

41. Cypher, B. L. 1993. Food item use by three sympatric canids in southern Illinois. Transactions of the Illinois State Academy of Science 86:139-144.

42. Fitch, H.S., Packard, R. L. 1955. The coyote on a natural area in northeastern Kansas. Transactions of the Kansas Academy of Science 58:211-221.

43. Green, J. S., Flinders, J. T. 1981. Diets of sympatric red foxes and coyotes in southeastern Idaho. The Great Basin Naturalist 41:251-254.

44. Grogan, M. E. 1997. Feeding Strategies of the Coyote (*Canis latrans*) in Western Tennessee. Master's thesis, University of Memphis, Memphis, Tennessee, USA.

45. Larson, R N., Brown, J. L., Karels, T., Riley, S.P. 2020. Effects of urbanization on resource use and individual specialization in coyotes (*Canis latrans)* in southern California. PLoS One 15:e0228881.

46. Balluffi-Fry, J., Nowell, L. B., Humphries, M. M. 2020. Eastern Coyotes (*Canis latrans* var.) consuming large ungulates in a multi-ungulate system. The Canadian Field-Naturalist 134:45-51.

47. Power, J. W. B. 2015. Genetic relationships, movement patterns, spatial dynamics and diet of the eastern coyote (*Canis latrans* var.) in Cape Breton Highlands National Park. Master's thesis, Acadia University, Wolfville, Nova Scotia, Canada.

48. Shi, Y., Hoareau, Y., Reese, E. M., Wasser, S. K. 2021. Prey partitioning between sympatric wild carnivores revealed by DNA metabarcoding: a case study on wolf (*Canis lupus*) and coyote (*Canis latrans*) in northeastern Washington. Conservation Genetics 22:293-305.

49. Peterson, M., Baglieri, M., Mahon, K., Sarno, R. J., Ries, L., Burman, P., Grigione, M. M. 2021. The diet of coyotes and red foxes in Southern New York. Urban Ecosystems 24:1-10.

50. Laundré, J.W., Calderas, J. M., Hernández, L. 2009. Foraging in the landscape of fear, the predator’s dilemma: where should I hunt? The Open Ecology Journal 2:1-6.

51. MacCracken, J. G. 1980. Feeding ecology of coyotes on the upper Snake River Plain, Idaho. Master's thesis, Colorado State University, Fort Collins, Colorado, USA.

52. MacCracken, J. G., Hansen, R. M. 1982. Seasonal foods of coyotes in southeastern Idaho: a multivariate analysis. The Great Basin Naturalist 42: 45-49.

53. Patterson, B. R., Benjamin, L. K, Messier, F. 1998. Prey switching and feeding habits of eastern coyotes in relation to snowshoe hares and white-tailed deer densities. Canadian Journal of Zoology 76:1885-1897.

54. Patterson, B. R., Messier, F. 2000. Factors influencing killing rates of white-tailed deer by coyotes in eastern Canada. The Journal of Wildlife Management 64:721-732.

55. Richer, M. C., Crête, M., Ouellet, J. P., Rivest, L. P., Huot, J. 2002. The low performance of forest versus rural coyotes in northeastern North America: inequality between presence and availability of prey. Ecoscience 9:44-54.

56. Bartel, R. A., Knowlton, F. F. 2005. Functional feeding responses of coyotes, *Canis latrans*, to fluctuating prey abundance in the Curlew Valley, Utah, 1977-1993. Canadian Journal of Zoology 83:569-578.

57. Bartel, R. A., Knowlton, F F., Stoddart, C. A. 2008. Long-term patterns in mammalian abundance in northern portions of the Great Basin. Journal of Mammalogy, 89: 1170-1183.

58. Bartel, R. A., Haddad, N. M., Wright, J. P. 2010. Ecosystem engineers maintain a rare species of butterfly and increase plant diversity. Oikos 119: 883-890.

59. Hurley, M. A., Zager, P. 2007. Southeast Mule Deer Ecology. Idaho Department of Fish and Game, Boise, Idaho, USA.

60. Hurley, M. A., Unsworth, J. W., Zager, P., Hebblewhite, M., Garton, E. O., Montgomery, D. M., Skalski, J.R., Maycock, C. L. 2011. Demographic response of mule deer to experimental reduction of coyotes and mountain lions in southeastern Idaho. Wildlife Monographs 178:1-33.

61. Johnson, M. K., Hansen, R. M. 1979. Coyote food habits on the Idaho National Engineering laboratory. The Journal of Wildlife Management 43:951-956.

62. Kelly, J. D., Gulsby, W. D., Killmaster, C. H., Bowers, J. W., Miller, K. V. Seasonal and spatial variation in diets of coyotes in central Georgia. Journal of the Southeastern Association of Fish and Wildlife Agencies 2:296-302.

63. Korschgen, L. J. 1957. Food habits of the coyote in Missouri. The Journal of Wildlife Management 21:424-435.

64. Kozlowski, A.J., Gese, E. M., Arjo, W. M. 2012. Effects of intraguild predation: evaluating resource competition between two canid species with apparent niche separation. International Journal of Ecology 2012: 629246.

65. Leopold, B. D., Krausman, P. R. 1986. Diets of 3 predators in Big Bend National Park, Texas. The Journal of Wildlife Management 50:290-295.

66. Moodie, J. D. 1983. Foraging strategies of selected carnivores. Master's thesis, University of Idaho, Moscow, Idaho, USA.

67. Morey, P. S., Gese, E. M., Gehrt, S. 2007. Spatial and temporal variation in the diet of coyotes in the Chicago metropolitan area. The American Midland Naturalist, 158:147-161.

68. Gehrt, S., Brown, J. L., Anchor, C. 2011. Is the urban coyote a misanthropic synanthrope? The case from Chicago. Cities and the Environment 4:3.

69. Rongstad, O. J. 1977. Coyote food habits in northwestern Wisconsin In Phillips, R., Jonkel, C. editors, Proceedings of the 1975 Predator Symposium. Montana Forest and Conservation Experiment Station, University of Montana, Missoula, Montana, USA.

70. Nellis, C. H., Keith, L. B. 1976. Population dynamics of coyotes in central Alberta, 1964-68. The Journal of Wildlife Management 40:389-399.

71. Poulle, M. L., Crête, M., Huot, J., Lemieux, R. 1993. Prédation exercée par le coyote, *Canis latrans*, sur le Cerf de Virginie, *Odocoileus virginianus*, dans un ravage en déclin de l'Est du Québec. Canadian Field Naturalist 107:177-185.

72. Reichel, J. D. 1991. Relationships among coyote food habits, prey populations, and habitat use. Northwest Science 65:133-137.

73. Springer, J. T., Smith, J. S. 1981. Summer food habits of coyotes in Central Wyoming. Great Basin Naturalist 41:449-456.

74. Springer, J.T., Wenger, C. R. 1981. Interactions between and some ecological aspects of coyotes and mule deer in central Wyoming. Wyoming Game and Fish Department, Laramie, Wyoming, USA.

75. Theberge, J. B.., Wedeles, C. H. R. 1989. Prey selection and habitat partitioning in sympatric coyote and red fox populations, southwest Yukon. Canadian Journal of Zoology 67:1285-1290.

76. Tiemeier, O. W. 1955. Winter foods of Kansas coyotes. Transactions of the Kansas Academy of Science 58:196-207.

77. Todd, A. 1985.Demographic and dietary comparisons of forest and farmland coyote, *Canis latrans*, populations in Alberta. Canadian Field-Naturalist 99:163-171.

78. Tremblay, J.-P., Crête, M., Huot, J. 1998. Summer foraging behaviour of eastern coyotes in rural versus forest landscape: a possible mechanism of source-sink dynamics. Ecoscience 5:172-182.

79. Van Vuren, D., Thompson Jr, S. E. 1982. Opportunistic feeding by coyotes. Northwest Science 56:131-135.

80. White, P., Ralls, K., White, C. A. V. 1995. Overlap in habitat and food use between coyotes and San Joaquin kit foxes. The Southwestern Naturalist 40:342-349.

81. Wigglesworth, R. R., McClennen, N., Anderson, S. H., Wachob, D. G. 2001. Comparison of coyote diets between two areas of Jackson Hole, Wyoming. Intermountain Journal of Sciences 6:355-367.

82. Windberg, L.A., Mitchell, C. D. 1990. Winter diets of coyotes in relation to prey abundance in southern Texas. Journal of Mammalogy 71:439-447.

83. Byerly, P. A., Lonsinger, R. C., Gese, E. M., Kozlowski, A. J., Waits, L. P. 2018. Resource partitioning between kit foxes (*Vulpes macrotis*) and coyotes (*Canis latrans*): a comparison of historical and contemporary dietary overlap. Canadian Journal of Zoology 96:497-504.

84. Cypher, B. L., Kelly, E. C., Westall, T. L., Job, C. L. 2018. Coyote diet patterns in the Mojave Desert: implications for threatened desert tortoises. Pacific Conservation Biology 24:44-54.

85. Graciano, E. M. E., García-Collazo, R. 2017. Dieta estacional del coyote (*Canis latrans*) en el Parque estatal Sierra de Tepotzotlán, estado de México. Biología, Ciencia y Tecnología 10:687-696.

86. Watine, L. N., Giuliano, W. M. 2017. Factors determining coyote (*Canis latrans*) diets. Open Journal of Ecology 7: 81249.

87. Gifford, S. J., Gese, E. M., Parmenter, R. R. 2020. Food habits of coyotes (*Canis latrans*) in the Valles Caldera National Preserve, New Mexico. The Southwestern Naturalist 64:122-130.

88. Neff, D. J., Woolsey, N. G. 1985. Effect of predation by coyotes on survival of antelope fawns on Anderson Mesa. Arizona Game and Fish Department, Phoenix, Arizona, USA.

89. Swingen, M. B., DePerno, C. S., Moorman, C. E. 2015. Seasonal coyote diet composition at a low-productivity site. Southeastern Naturalist 14:397-404.

90. Proulx, G., Parr, S. 2018. Is Livestock an Important Food Resource for Coyotes and Wolves in Central Eastern Alberta Counties with Predator Control Bounties? Canadian Wildlife Biology & Management 7:31-45.

91. Sivy, K. J. 2015. Direct and indirect effects of wolves on interior Alaska's mesopredator community. Dissertation, University of Alaska, Fairbanks, Alaska, USA.

92. Smith, J. A., Thomas, A. C., Levi, T., Wang, Y., Wilmers, C. C. 2018. Human activity reduces niche partitioning among three widespread mesocarnivores. Oikos 127:890-901.

93. Larson, R. N., Brown, J. L., Karels, T., Riley, S. P. 2020. Effects of urbanization on resource use and individual specialization in coyotes (*Canis latrans*) in southern California. PLoS One 15:e0228881.

94. Cherry, M. J., Turner, K. L., Howze, M. B., Cohen, B. S., Conner, L. M., Warren, R. J. 2016. Coyote diets in a longleaf pine ecosystem. Wildlife Biology 22:64-70.

95. Alvarez-Castaneda, S. T., Gonzalez-Quintero, P. 2006. Winter-spring food habits of an island population of coyote *Canis latrans* in Baja California, Mexico. Journal of Arid Environments 60:397-404.

96. Boggess, E. 1975. Some population parameters of Iowa coyotes and an analysis of reported livestock losses.Master's thesis, Iowa State University, Ames, Iowa, USA.

97. McGrath, M., Dredge, M., Curran, R., Reynolds, J. 2010. Harvest providing valuable insight into coyote ecology. Department of Environment and Conservation, St. Johns, Newfoundland, Canada.

98. Aranda, M., Rivera, N. L., De Buen, L. L. 1995. Hábitos alimentarios del coyote (*Canis latrans*) en la Sierra del Ajusco, México. Acta Zoológica Mexicana 65:89-99.

99. Arjo, W. M., Pletscher, D. H., Ream, R. R. 2002. Dietary overlap between wolves and coyotes in northwestern Montana. Journal of Mammalogy 83:754-766.

100. Arnaud, G. 1993. Alimentación del coyote (*Canis latrans*) en Baja California Sur, México. Avances en el estudio de los mamíferos de México. Publicaciones Especiales 1:205-215.

101. Atkinson, D. E. 1976. Population dynamics and predator-prey relationships of the Carmen Mountains white-tailed deer. Master's thesis, Texas A&M University, College Station, Texas, USA.

102. Azevedo, F. C., Lester, V., Gorsuch, W., Lariviere, S., Wirsing, A. J., Murray, D. L. 2006. Dietary breadth and overlap among five sympatric prairie carnivores. Journal of Zoology 269:127-135.

103. Bailey, V. 1936. The mammals and life zones of Oregon. North American Fauna No. 55. Bureau of Biological Survey, U. S. Department of Agriculture, Washington, D. C., USA.

104. Baker, K. 2014. Seasonal variation in coyote (*Canis latrans*) diet at the San Joaquin River, San Luis, and Merced National Wildlife Refuges. Master's thesis, California State University, Stanislaus, California, USA.

105. Berg, W.E., Chesness, R. A. 1978. Ecology of coyotes in northern Minnesota. In Bekoff, M. editor, Coyotes: biology, behavior, and management. Academic Press, New York, New York, USA; p. 229-247.

106. Bergeron, J.-M., Demers, P. 1981. Le regime alimentaire du coyote (*Canis latrans*) et du chien errant (*C. familiaris*) dans le sud du Quebec. Canadian Field Naturalist 95:172-177.

107. Best, T. L., Hoditschek, B., Thomas, H. H. 1981. Foods of Coyotes (*Canis latrans*) in Oklahoma. The Southwestern Naturalist 26:67-69.

108. Bixel, K.D. 1995. Trophic ecology of adult coyotes (*Canis latrans*) in south-central Pennsylvania. Master's thesis, Shippensburg University, Shippensburg, Pennsylvania, USA.

109. Bogan, D.A. 2012. The suburban coyote syndrome, from anecdote to evidence: Understanding ecology and human safety to improve coexistence. Dissertation, Cornell University, Ithaca, New York, USA.

110. Boisjoly, D., Ouellet, J. P., Courtois, R. 2010. Coyote habitat selection and management implications for the Gaspésie caribou. The Journal of Wildlife Management, 74:3-11.

111. Bollin-Booth, H. A. 2007. Diet analysis of the coyote (*Canis latrans*) in metropolitan park systems of northeast Ohio.Master's thesis, Cleveland State University, Cleveland, Ohio, USA.

112. Bond, R. M. 1939. Coyote food habits on the Lava Beds National Monument. The Journal of Wildlife Management 3:180-198.

113. Bowen, W. D. 1978. Social organization of the coyote in relation to prey size. Dissertation, University of British Columbia, Vancouver, British Columbia, Canada.

114. Bowen, W. D. 1981. Variation in coyote social organization: the influence of prey size. Canadian Journal of Zoology 59:639-652.

115. Bowyer, R. T., McKenna, S. E., Shea, M. E. 1983. Seasonal changes in coyote food habits as determined by fecal analysis. American Midland Naturalist 109:266-273.

116. Boyd, D. 1982. Food habits and spatial relations of coyotes and a lone wolf in the Rocky Mountains. Master's thesis, University of Montana, Missoula, Montana, USA.

117. Bridger, K. E. 2005. A comparative study of the dietary habits and helminth fauna of Canada lynx (*Lynx canadensis*), red fox (*Vulpes vulpes*) and eastern coyote (*Canis latrans*) on insular Newfoundland. Master's thesis, Memorial University of Newfoundland, St. Johns, Newfoundland, Canada.

118. Brillhart, D. E., Kaufman, D. W. 1995. Spatial and seasonal variation in prey use by coyotes in north-central Kansas. The Southwestern Naturalist 40:160-166.

119. Buck, W. S., Kitts, J. R. 2004. Citizen research of Chicago coyotes: a model program. In Shaw, W., Harris, L., VanDruff, L., editors, 4th International Urban Wildlife Symposium.School of Natural Resources, College of Agriculture and Life Sciences, University of Arizona, Tucson, Arizona, USA.

120. Caturano, S. L. 1983. Habitat and home range use by coyotes in eastern Maine. Master's thesis, University of Maine, Orono, Maine, USA.

121. Cepek, J. D. 2004. Diet composition of coyotes in the Cuyahoga Valley National Park, Ohio. Ohio Journal of Science 104:60-64.

122. Chronert, J. M. 2007. Ecology of the coyote (*Canis latrans*) at Wind Cave National Park. Master's thesis, South Dakota State University, Brookings, South Dakota, USA.

123. Cowan, I. McT. 1943. Report on Game Conditions in Banff, Jasper and Kootenay National Parks. Canadian National Parks Service, Vancouver, British Columbia, Canada.

124. Cox, J. J. 2003. Community dynamics among reintroduced Elk, White-tailed Deer, and Coyote in southeastern Kentucky. Dissertation, University of Kentucky, Lexington, Kentucky, USA.

125. Crabtree, R. L., Sheldon, J. W. 1999. The ecological role of coyotes on Yellowstone’s northern range. Yellowstone Science 7:15-23.

126. Crête, M., Lemieux, R. 1994. Dynamique de population des coyotes colonisant la péninsule gaspésienne. 1994: Ministère de l'Environnement et de la faune, Québec, Québec, Canada.

127. Crimmins, S. M., Edwards, J. W., Houben, J. M. 2012. *Canis latrans* (coyote) habitat use and feeding habits in central West Virginia. Northeastern Naturalist 19:411-420.

128. Crombie, K. 1985. The ecology of the coyote (*Canis atrans*) in intensively cultivated areas in eastern Fresno County, California. Master's thesis, California State University, Fresno, California, USA.

129. Crossett, R. L., Elliott, C. 1991. Winter food habits of red foxes and coyotes in central Kentucky. Proceedings of Annual Conference of Southeastern Fish and Wildlife Agencies 45:97-103.

130. Cruz-Espinoza, A., González, G., Santos, A. 2008. Dieta y abundancia relativa del coyote (*Canis latrans*) en un bosque templado de la sierra Norte de Oaxaca, México. Avances en el estudio de los mamíferos de México II:239-252.

131. Cuatepitzi-Sanchez, H., Servin, J. no date. Habitos alimentarios del coyote (*Canis latrans*) en el Parque Nacionale Iztaccihuatl-Popocatepetl Zoquiapan y Anexas, Mexico. Universidad Autónoma Metropolitana, Xochimilco, Mexico.

132. Cunningham, S. C., Kirkendall, L. Ballard, W. B. 2006. Gray fox and coyote abundance and diet responses after a wildfire in central Arizona. Western North American Naturalist 66:169-180.

133. Delibes, M., Hernández, L., Hiraldo, F. 1986. Datos preliminares sobre la ecología del coyote y gato montés en el sur del Desierto de Chihuahua, México. Historia Natural 6:77-82.

134. Dibello, F., Arthur, S., Krohn, W. B. 1990. Food habits of sympatric coyotes, *Canis latrans*, red foxes, *Vulpes vulpes*, and bobcats, *Lynx rufus*, in Maine. Canadian Field-Naturalist 104:403-408.

135. Dowd, J. L. B. 2010. Coyote diet and movements in relation to winter recreation in northwestern Wyoming: implications for lynx conservation. Master's thesis, Utah State University, Logan, Utah, USA.

136. Dowd, J. L., Gese, E. M. 2012. Seasonal variation of coyote diet in northwestern Wyoming: implications for dietary overlap with Canada lynx? Northwest Science, 86: 289-299.

137. Drewek, J. R. 1980. Behavior, population structure, parasitism, and other aspects of coyote ecology in southern Arizona. Dissertation, University of Arizona, Tucson, Arizona, USA.

138. Dumond, M. 2001. Alimentation, démographie et condition physique chez une population de coyotes (*Canis latrans*) dans le Grand Écosysteme de Kouchibouguac, Nouveau-Brunswick, Canada. Master's thesis, De L'Universite de Moncton, Moncton, New Brunswick, Canada.

139. Dumond, M., Villard, M.-A., É. Tremblay, É. 2001. Does coyote diet vary seasonally between a protected and an unprotected forest landscape? Ecoscience 8:301-310.

140. Edwards Jr, D. A. 1996. Ecological relationships among bobcats, coyotes, and gray foxes in central Mississippi. Master's thesis, Mississippi State University, Mississippi State, Mississippi, USA.

141. Elliott, C. L. 1984. Wildlife food habits and habitat use on revegetated stripmine land in Alaska. Dissertation, University of Alaska, Fairbanks, Alaska, USA.

142. Elliott, C. L., Guetig, R. 1990. Summer food habits of coyotes in Idaho's River of No Return Wilderness area. The Great Basin Naturalist 50:63-65.

143. Esparza García, J. 1991. Variaciones estacionales en la dieta de mamíferos carnívoros en la estación científica las joyas. Tesis profesional, Universidad de Guadalajara, Guadalajara, Mexico.

144. Fedriani, J. M., Fuller, T. K., Sauvajot, R. M., York, E. C. 2000. Competition and intraguild predation among three sympatric carnivores. Oecologia 125:258-270.

145. Fedriani, J. M., Fuller, T. K., Sauvajot, R. M. 2001. Does availability of anthropogenic food enhance densities of omnivorous mammals? An example with coyotes in southern California. Ecography 24:325-331.

146. Fichter, E., Schildman, F., Sather, J. H. 1955. Some feeding patterns of coyotes in Nebraska. Ecological Monographs 25:2-37.

147. Field, S. A. 2004. Population ecology of eastern coyotes (*Canis latrans*) on Prince Edward Island. Master's thesis, Univerity of Prince Edward, Charlottetown, Prince Edward Island, Canada.

148. Garwood T, Ziobro, R., Keene, K., Waller, A., Pauli, J. N. 2015. Diet, habitat use, and size of an urban population of coyotes obtained noninvasively. Urban Naturalist, 5:1-8.

149. Gerads, J. R., Jenks, J. A., Watters, B. K. 2001. Food habits of coyotes inhabiting the Black Hills and surrounding prairies in western South Dakota. Proceedings of the South Dakota Academy of Science 80:95-108.

150. Gese, E. M. 1987. Ecology of coyotes in southeastern Colorado. Dissertation, University of Wisconsin, Madison, Wisconsin, USA.

151. Gese, E. M., Rongstad, O. J., Mytton, W. R. 1988. Relationship between coyote group size and diet in southeastern Colorado. The Journal of Wildlife Management 52:647-653.

152. Gier, H. 1975. Coyote. In Fox, M. W., editor. The wild canids: their systematics, behavioral ecology and evolution. Von Nostrand Rheinholt Company, New York. p. 247-262.

153. Gifford, S. J. 2013. Ecology of coyotes on the Valles Caldera National Preserve, New Mexico: implications for elk calf recruitment. Master's thesis, Utah State University, Logan, Utah, USA.

154. Glass, J. H. 1977. Diets of coyotes (*Canis latrans*) in the Edwards Plateau and Trans-Pecos regions of Texas. Master's thesis, Sul Ross State University, Alpine, Texas, USA.

155. Glatz, R. G. 1976. The coyotes of Connecticut. Master's thesis, University of Connecticut, Storrs, Connecticut, USA.

156. González Ruvalcaba, S. 2008. Dieta del coyote (*Canis latrans*) en la laguna de Santiaguillo, Durango, México. Licencio de Biologica tesis, University de Guadalajara, Guadalajara, Mexico.

157. Grajales-Tam, K. M., González-Romero, A. 2014. Determinación de la dieta estacional del coyote (*Canis latrans*) en la región norte de la Reserva de la Biosfera Mapimí, México. Revista Mexicana de Biodiversidad 85:553-564.

158. Grater, R. K. 1943. Coyote foods near Boulder Dam. The Journal of Wildlife Management 7:422-423.

159. Guerrero, S., Badii, M. H., Zalapa, S. S., Arce, J. A. 2004. Variación espacio-temporal en la dieta del coyote en la costa norte de Jalisco, México. Acta Zoológica Mexicana 20:145-157.

160. Hall, D. I., Newsom, J. D. 1978. Coyote in Louisiana. Louisiana Agriculture 21:4-5.

161. Hamilton, Jr., W. J. 1974. Food habits of the coyote in the Adirondacks. New York Fish and Game Journal 21:177-181.

162. Harrison, D. J., Harrison, J. A. 1984. Foods of adult Maine coyotes and their known-aged pups. The Journal of Wildlife Management 48:922-926.

163. Hawthorne, V. M. 1971. Coyote movements in Sagehen Creek basin, northeastern California. California Fish and Game 57:154-161.

164. Hawthorne, V. M. 1972. Coyote food habits in Sagehen Creek basin, northeastern California. California Fish and Game 58:4-12.

165. Henke, S. E. 2002. Coyotes: Friends or Foe of Northern Bobwhite in Southern Texas. In DeMaso S. J., Kuvlesky, Jr. W. P., Hernandez, F., Berger, M. E. Quail V: Proceedings of the Fifth National Quail Symposium 5. Texas Parks and Wildlife Department, Austin, Texas, USA. p. 57–60.

166. Hernández, L., Delibes, M. 1994. Seasonal food habits of coyotes, *Canis latrans*, in the Bolsón de Mapimí, southern Chihuahuan Desert, Mexico. [Zeitschrift für Säugetierkunde](https://www.biodiversitylibrary.org/bibliography/85187) 59:82-86.

167. Hernández, L., Delibes, M., Hiraldo, F. 1994. Role of reptiles and arthropods in the diet of coyotes in extreme desert areas of northern Mexico. Journal of Arid Environments 26:165-170.

168. Hernández, L., Parmenter, R. R., Dewitt, J. W., Lightfoot, D. C., Laundré, J. W. 2002. Coyote diets in the Chihuahuan Desert, more evidence for optimal foraging. Journal of Arid Environments 51:613-624.

169. Montoya Gómez G, Hernández Ruiz JF, Velasco Pérez A, Reygadas L, Ramos Maza T. 2006. Organización comunitaria para la conservación forestal: estudio de caso en la Selva Lacandona de Chiapas, México. Papeles de población 12:177-204.

170. Hidalgo-Mihart, M. G., Cantú-Salazar, L., López-González, C. A., Martínez-Meyer, E., González-Romero, A. 2001. Coyote (*Canis latrans*) food habits in a tropical deciduous forest of western Mexico. American Midland Naturalist 146:210-216.

171. Hidalgo-Mihart, M. G., Cantú-Salazar, L., López-González, C. A., Martínez-Gutierrez, P. G., Fernandez, E. C., , González-Romero, A. L. Coyote habitat use in a tropical deciduous forest of western Mexico. The Journal of Wildlife Management 70:216-221.

172. Hilton, H. 1976. The physical characteristics, taxonomic status and food habits of the eastern coyote in Maine. Master's thesis, University of Maine, Orono, Maine, USA.

173. Richens, V. B., Hugie, R. D. 1974. Distribution, taxonomic status, and characteristics of coyotes in Maine. The Journal of Wildlife Management 38:447-454.

174. Hinton, J. W. 2014. Red wolf (*Canis rufus*) and coyote (*Canis latrans*) ecology and interactions in northeastern North Carolina. Dissertation, University of Georgia, Athens, Georgia, USA.

175. Hoerath, J., Causey, M. 1991. Seasonal diets of coyotes in western central Alabama. Proceedings of the Annual Conference of the Southeastern Association of Fish and Wildlife Agencies 45:91-96.

176. Holle, D. 1978. Food habits of coyotes in an area of high fawn mortality. Proceedings of the Oklahoma Academy of Science 58:11-15.

177. Huebschman, J. J., Hygnstrom, S. E., Gubanyi, J. A. 1997. Coyote food habits at DeSoto National Wildlife Refuge, Nebraska. The Prairie Naturalist 29:99-109.

178. Huegel, C. N. 1979. Winter ecology of coyotes in northern Wisconsin. Master's thesis, University of Wisconsin, Madison, Wisconsin, USA.

179. Johnson, M. K., Hansen, R. M. 1977. Food of coyotes in the lower Grand Canyon, Arizona. Journal of the Arizona Academy of Science 12:81-83.

180. Kamler, J. F., Gipson, P. S. 2000. Space and habitat use by resident and transient coyotes. Canadian Journal of Zoology 78:2106-2111.

181. Kamler, J. F., Gipson, P. S., Perchellet, C. C. 2002. Seasonal Food Habits of Coyotes. The Prairie Naturalist 34:75-84.

182. Kamler, J.F. 1998. Ecology and interspecific relationships of mammalian predators on Fort Riley Military Reservation, Kansas. Master's thesis, Kansas State University, Manhattan, Kansas, USA.

183. Kamler, J. F., Ballard, W. B., Gilliland, R. L., Mote, K. 2003. Spatial relationships between swift foxes and coyotes in northwestern Texas. Canadian Journal of Zoology, 81:168-172.

184. Kamler, J. F., Ballard, W. B., Wallace, M. C., Gilliland, R. L., Gipson, P. S. 2007. Dietary overlap of swift foxes and coyotes in northwestern Texas. The American Midland Naturalist 158:139-146.

185. Kamler, J. F., Klare, U., Ballard, W. B., Wallace, M. C., Gipson, P. S. 2014. Comparison of coyote (*Canis latrans*) diets in continuous and fragmented short-grass prairie in the Texas Panhandle. Texas Journal of Science 66:25-41.

186. Kelly, J. D. 2012. Seasonal food habits of the coyote (*Canis latrans*) on differing landscapes in the Piedmont region of Georgia. Master's thesis, University of Georgia, Athens, Georgia, USA.

187. Kitchen, A. M., Gese, E. M., Schauster, E. R. 1999. Resource partitioning between coyotes and swift foxes: space, time, and diet. Canadian Journal of Zoology 77:1645-1656.

188. Kitchen, A., 2000. Resource partitioning between coyotes and swift foxes: Space, time, and diet.Master's thesis, Utah State University, Logam Utah, USA

189. Knowlton, F. F. 1964. Aspects of coyote predation in south Texas with special reference to white-tailed deer. Dissertation, Purdue University, Lafeyette, Indian, USA.

190. Koehler, G. M., Hornocker, M. G. 1991. Seasonal resource use among mountain lions, bobcats, and coyotes. Journal of Mammalogy 72:391-396.

191. Kuenzi, A. J. 1997. Diet of coyotes at Lemoore Naval Air Station. Transactions Western Section of The Wildlife Society 33:9-11.

192. LaPierre, L. 1985. Fall and winter food habits of the eastern coyote *Canis latrans* in southeastern New Brunswick. Proceedings Nova Scotia Institute of Science 35:71-74.

193. Latham, A. D. M., Latham, M. C., Knopff, K. H., Hebblewhite, M., Boutin, S. 2013. Wolves, white‐tailed deer, and beaver: implications of seasonal prey switching for woodland caribou declines. Ecography 36:1276-1290.

194. Latham, A.D.M., Latham, M. C., Boyce, M. S., Boutin, S. 2011. The Role of Predation in Woodland Caribou Population Declines in Northeastern Alberta – Coyotes. Petroleum Technology Alliance of Canada, Alberta, Canada.

195. Lee, R., Kennedy, M. 1986. Food habits of the coyote in Tennessee. Proceedings of the Annual Conference of the Southeastern Association of Fish and Wildlife Agencies 40:364-372.

196. Lewis, T., Jensen, W. F., Keehr, K. A., Seabloom, R. W. 1994. Summer and fall food habits of coyotes in southwestern North Dakota. The Prairie Naturalist 26:287-292.

197. List, R., Manzano-Fisher, P., Macdonald, D. W. 2003. Coyote and kit fox diets in prairie dog towns and adjacent grasslands in Mexico. In Sovada, M. and Carbyn, L. editors. The swift fox: ecology and conservation of the swift foxes in a changing world, University of Regina Press, Regina, Saskatchewan. p. 183-188.

198. Litvaitis, J. A. 1981. A comparison of coyote and bobcat food habits in the Wichita Mountains, Oklahoma. in Proceedings of the Oklahoma Academy of Science 61:81-82.

199. Litvaitis, J. A., Harrison, D. J. 1989. Bobcat–coyote niche relationships during a period of coyote population increase. Canadian Journal of Zoology 67:1180-1188.

200. López Soto, J. H. 2000. Predación de coyote *Canis latrans texensis* en venado cola blanca *Odocoileus virginianus texanus* en Anáhuac, Nuevo León, México. Maestros tesis, Universidad Autónoma de Nuevo León, San Nocolas de los Garza, Nuev León, Mexico.

201. López-Soto, J., García-Hernández, R., Badii, M. H. 2001. Dieta invernal del Coyote (*Canis latrans*) en un rancho del noreste de México. Ciencia Nicolaíta 27:27-35.

202. Lovell, C. D. 1996. Bobcat, coyote, and gray fox micro-habitat use and interspecies relationships in a managed forest in central Mississippi. Master's thesis, Mississippi State University, Mississippi State, Mississippi, USA.

203. Lukasik, V. M. 2009. The diet and human interactions of urban coyotes in Calgary, Alberta. Master's thesis, University of Calgary, Calgary, Alberta, Canada.

204. Lukasik, V. M., Alexander, S. M. 2011. Human–coyote interactions in Calgary, Alberta. Human Dimensions of Wildlife 16:114-127.

205. Makar, P. W. 1980. Bobcat and coyote food habits and habitat use in Rocky Mountain National Park. Master's thesis, Colorado State University, Fort Collins, Colorado, USA.

206. Manning, D. L. 2007. A comparative ecological study between coyotes (*Canis latrans*) in a protected and urban habitat: a closer look at enteric parasites and diet between Florida coyotes. Master's thesis, University of South Florida, Tampa, Florida, USA.

207. García, J. A. M., Martínez, G. D., Rosas, O. C., Arámbula, L. A., Bender, L. C. 2014. Use of prey by sympatric bobcat (*Lynx rufus*) and coyote (*Canis latrans*) in the Izta-Popo National Park, Mexico. The Southwestern Naturalist 59:167-172.

208. Martínez-Vázquez, J., González-Monroy, R. M., Díaz-Díaz,D. 2010. Hábitos alimentarios del coyote en el parque nacional Pico de Orizaba. Therya 1:145-154.

209. Mathwig, H. J. 1973. Food and population characteristics of Iowa coyotes. Iowa State Journal of Research 47:167-189.

210. McCue, A. J. 2013. General Patterns Among Generalists: What is Revealed by Spatial Models of Coyotes? Master's thesis, Memorial University of Newfoundland, St. John's, Newfoundland and Labrador, Canada.

211. McKinney, T., Smith, T. W. 2007. Diets of sympatric bobcats and coyotes during years of varying rainfall in central Arizona. Western North American Naturalist 67:8-15.

212. McLean, D. D. 1934. Predatory animal studies. California Fish and Game Journal 20:30-36.

213. Mcvey, J. 2011. Assessing Food Habits of Red Wolves (*Canis rufus*) and Coyotes (C*anis latrans*) in Eastern North Carolina. Master's thesis, North Carolina State University, Raleigh, North Carolina, USA.

214. Meinzer, W. P., Ueckert, D. N., Flinders, J. T. 1975. Foodniche of coyotes in the rolling plains of Texas. Journal of Range Management 28:22-27.

215. Messier, F., Barrette, C. 1982. The social system of the coyote (*Canis latrans*) in a forested habitat. Canadian Journal of Zoology 60:1743-1753.

216. Messier, F., Barrette, C., Huot, J. 1986. Coyote predation on a white-tailed deer population in southern Quebec. Canadian Journal of Zoology 64:1134-1136.

217. Michaelson, K.A., Goertz, J. 1977. Food habits of coyotes in northwest Louisiana. Louisiana Academy of Sciences 40:77-81.

218. Monroy, V., Ortega, M., Velázquez, A. 2003. Dieta y abundancia relativa del coyote: un dispersor potencial de semillas. Instituto Nacional de Ecologia y Cambio Climatico. p. 565-591.

219. Moore, G., Millar, J. 1986. Food habits and average weights of a fall-winter sample of eastern coyotes, *Canis latrans*. Canadian Field-Naturalist 100:105-106.

220. Murie, O. J. 1935. Food habits of the coyote in Jackson Hole, Wyo. Circular 362, US Department of Agriculture, Washington, D. C., USA.

221. Murie, A. 1940. Ecology of the coyote in the Yellowstone. Fauna of the National Parks of the United States No. 4, National Park Service, Washington, D. C., USA.

222. Murie, O. J.1945. Notes on coyote food habits in Montana and British Columbia. Journal of Mammalogy 26:33-40.

223. Murie, A., Coyote food habits on a southwestern cattle range. Journal of Mammalogy, 1951. 32(3): p. 291-295.

224. Murray, M., et al., Poor health is associated with use of anthropogenic resources in an urban carnivore. Proceedings of the Royal Society B: Biological Sciences, 2015. 282(1806): p. 20150009.

225. Murray, M., Cembrowski, A., Latham, A. D. M., Lukasik, V. M., Pruss, S., St. Clair, C. C. 2015. Greater consumption of protein‐poor anthropogenic food by urban relative to rural coyotes increases diet breadth and potential for human–wildlife conflict. Ecography 38:1235-1242.

226. Neale, J. C. C., Sacks, B. N. 2001. Resource utilization and interspecific relations of sympatric bobcats and coyotes. Oikos 94:236-249.

227. Sacks, B. N., Neale, J. C. 2002. Foraging strategy of a generalist predator toward a special prey: coyote predation on sheep. Ecological Applications 12:299-306.

228. Sacks, B. N. 1996. Ecology and behavior of coyotes in relation to depredation and control on a California sheep ranch. Master's thesis, University of California, Berkeley, California, USA.

229. O'Connell, Jr., A. F., Harrison, D. J., Connery, B., Anderson, K. A. 1992. Food use by an insular population of coyotes. Northeast Wildlife 49:36-42.

230. Ortega, J. C. 1987. Coyote food habits in southeastern Arizona. The Southwestern Naturalist 32:152-155.

231. Owens, K. M. 2006. Seasonal dietary composition of the eastern Coyote (*Canis latrans*) on the Berry College Campus in northwestern Georgia. Master's thesis, University of Tennessee, Chattanooga, Tennessee, USA.

232. Ozoga, J. J., Harger, E. M. 1966. Winter activities and feeding habits of northern Michigan coyotes. The Journal of Wildlife Management 30:809-818.

233. Parke, R. W. 2004. Food habits of coyotes and red foxes in western South Dakota as determined by fecal analysis. Master's thesis, South Dakota State University, Brookings, South Dakota, USA.

234. Parker, G. 1986. The seasonal diet of coyotes, *Canis latrans*, in northern New Brunswick. Canadian Field-Naturalist 100:74-77.

235. Parker, T. S. 1999. Food habits of the coyote (*Canis latrans*) in urban and suburban areas of western Tennessee. Master's thesis, University of Memphis, Memphis, Tennessee, USA..

236. Pederson, J. C., Tuckfield, R. C. 1983. A comparative study of coyote food habits on two Utah deer herds. The Great Basin Naturalist 43:432-437.

237. Perrine III, J. D. 2005. Ecology of red fox (*Vulpes vulpes*) in the Lassen Peak region of California, USA. Dissertation, University of California, Berkeley, California, USA.

238. Person, D. K. 1988. Home range, activity, habitat use, and food habits of eastern coyotes in the Champlain valley region of Vermont. Master's thesis, University of Vermont, Burlington, Vermont, USA.

239. Phillips, M., Hubert Jr, G. 1980. Winter food habits of coyotes in southeastern Illinois. Transactions of the Illinois State Academy of Science 73:80-84.

240. Polechla, P. J. 1980. Food habits of the Coyote (*Canis latrans* Say) in east-central New Mexico with special reference to size and sex differences. Master's thesis, Eastern New Mexico University, Potrtales, New Mexico, USA.

241. Quinn, T. 1997. Coyote (*Canis latrans*) food habits in three urban habitat types of western Washington. Northwest Science 71:1-5.

242. Ribic, C. A. 1978. Summer foods of Coyotes at Rocky Flats, Colorado. The Southwestern Naturalist 23:152-153 .

243. Rogers, J. G. 1965. Analysis of the coyote population of Dona Ana County, New Mexico. Master's thesis, New Mexico State University, Las Cruces, New Mexico, USA.

244. Paez, M. A. S. 1987. Hábitos alimenticios de la zorra, coyote y gato montés en la Sierra Tarasca. Revista Mexicana de Ciencias Forestales 12:117-132.

245. Samson, C., Crete, M. 1997. Summer food habits and population density of coyotes, *Canis latrans*, in boreal forests of southeastern Québec. The Canadian Field-Naturalist 111:227-233.

246. Sanabria, B., Ortega-Rubio, A., Arguelles-Méndez, C. 1995. Food habits of the coyote in the Vizcaíno Desert, México. Ohio Journal of Science 95:289-291.

247. Santana, E. 2010. Food habits and anthropogenic supplementation in the diet of coyotes (*Canis latrans*) along an urban-rural gradient. Master's thesis, Auburn University, Auburn, Alabama, USA.

248. Schoch, B. N. 2003. Diet, age, and reproduction of mesomammalian predators in response to intensive removal during the quail nesting season. Master's thesis, University of Georgia, Athens, Georgia, USA.

249. Schrecengost, J. D. 2007. Home range and food habits of the coyote (*Canis latrans*) at the Savannah River Site, South Carolina. Master's thesis, University of Georgia, Athens, Georgia, USA.

250. Schrecengost, J. D., Kilgo, J. C., Mallard, D., Ray, H. S., Miller, K. V. 2008. Seasonal food habits of the coyote in the South Carolina coastal plain. Southeastern Naturalist 7:135-144.

251. Servín, J., Huxley, C. 1991. La dieta del coyote en un bosque de encino-pino de la Sierra Madre Occidental de Durango, México. Acta Zoológica Mexicana 44:1-26.

252. Shoemaker, S. 1985. Food selection, home range, and movements of coyotes on and off a sanctuary in Klamath Basin. Master's thesis, Oregon State University, Corvallis, Oregon, USA.

253. Short, H. L. 1979. Food habits of coyotes in a semidesert grass-shrub habitat. USDA Forest Service Research Note RM 364.

254. Small, R. L. 1971. Interspecific competition among three species of Carnivora on the Spider Ranch, Yavapai County, Arizona. Master's thesis, University of Arizona, Tucson, Arizona, USA.

255. Smith, J. 1990. Coyote diets associated with seasonal mule deer activities in California. California Fish and Game 76:78-82.

256. Smith, R., Kennedy, M. 1983. Food habits of the coyote (*Canis latrans*) in western Tennessee. Journal of the Tennessee Academy of Science 58:27-28.

257. Snodgrass, K. 1997. Food habits of Coyotes (*Canis latrans*) on barrier islands. Master's thesis, Texas A & M University, Corpus Christi, Texas, USA.

258. Souther, O., Wiggers, S. 2012. Comparative diet analysis of *Canis rufus* and *Canis latrans*. Proceedings of The National Conference On Undergraduate Research, Weber State University, Ogden Utah. p. 312-317.

259. Sovada, M. A., Telesco, D. J., Roy, C. C. 2000. Coyote, *Canis latrans*, use of commercial sunflower, *Helianthus* spp., seeds as a food source in western Kansas. Canadian Field-Naturalist 114:697-699.

260. Sperry, C. C. 1933. Autumn food habits of coyotes, a report of progress, 1932. Journal of Mammalogy 14:216-220.

261. Sperry, C. C. 1934. Winter food habits of coyotes: a report of progress, 1933. Journal of Mammalogy 15:286-290.

262. Steinmann, K. W., Cegelski, M. J., Deliset, P. R. 2011. Dietary patterns of Pennsylvania coyotes during winter. Keystone Journal of Undergraduate Research 1:13-18.

263. Stratman, M. R., Pelton, M. R. 1997. Food habits of coyotes in northwestern Florida. Proceedings Annual Conference of Southeastern Association of Fish and Wildlife Agencies 51:269-275.

264. Thornton, D. H., Sunquist, M. E., Main, M. B. 2004. Ecological separation within newly sympatric populations of coyotes and bobcats in south-central Florida. Journal of Mammalogy 85:973-982.

265. Thurber, J. M., Peterson, R. O., Woolington, J. D., Vucetich, J. A. 1992. Coyote coexistence with wolves on the Kenai Peninsula, Alaska. Canadian Journal of Zoology 70:2492-2498.

266. Toweill, D. E., Anthony, R. G. 1988. Coyote food in coniferous forest in Oregon. The Journal of Wildlife Management 52:507-512.

267. Turkowski, F. J. 1980. Carnivora food habits and habitat use in ponderosa pine forests. USDA Forest Service Research Note RM 215.

268. Turner, M. M., Rockhill, A. P., Deperno, C. S., Jenks, J. A., Klaver, R. W., Jarding, A. R., Grovenburg, T. W., Pollock, K. H. 2011. Evaluating the effect of predators on white-tailed deer: movement and diet of coyotes. The Journal of Wildlife Management 75:905-912.

269. Van Vuren, D.1991. Yellow-bellied marmots as prey of coyotes. American Midland Naturalist 125:135-139.

270. Vangilder, C. L. 2006. Coyote and bobcat food habits and the effects of an intensive predator removal on white-tailed deer recruitment in northeastern Alabama. Master's thesis, University of Georgia, Athens, Georgia, USA.

271. Vaughan, T. 1954. Mammals of the San Gabriel Mountains of California. University of Kansas Museum of Natural History Publication 7513-582.

272. Warsen, S. A. 2012. Evolving niche of coyotes in the Adirondack Mountains of New York: long-term dietary trends and interspecific competition. Master's thesis, State University of New York, Syracuse, New York, USA.

273. Weintraub, J. D. 1986. Coyote diets, five years later, at Cuyamaca Rancho State Park. Bulletin of the Southern California Academy of Science 85:152-157.

274. Westmoreland, D. A., Woolf, A. 1981. Presence of fawn remains and other food items in coyote scats from southern Illinois. Transactions of the Illinois State Academy of Science 81:63-66.

275. Wiley, J. E.I . 1975. The history and status of the eastern coyote in New Hampshire. In Chambers, R. E., editor. Transactions of the Eastern Coyote Workshop, New Haven, Connecticutt. p. 20-32.

276. Witczuk, J., Pagacz, S., Mills, L. S. 2013. Disproportionate predation on endemic marmots by invasive coyotes. Journal of Mammalogy 94:702-713.

277. Witczuk, J., Pagacz, S., Gliwicz, J., Mills, L. S. 2015. Niche overlap between sympatric coyotes and bobcats in highland zones of Olympic Mountains, Washington. Journal of Zoology 297:176-183.

278. Witmer, G. W., DeCalesta, D. S. 1986. Resource use by unexploited sympatric bobcats and coyotes in Oregon. Canadian Journal of Zoology 64:2333-2338.

279. Witmer, G., Pipas, M., Hayden, A. 1995. Some observations on coyote food habits in Pennsylvania. Journal of the Pennsylvania Academy of Science 69:77-80.

280. Wooding, J. B., Hill, E. P., Sumner, P. W. 1984. Coyote food habits in Mississippi and Alabama. Proceedings of the Annual Conference of Southeast Associations of Fish and WIldlife Agencies 38:182-188.

281. Young, S. P., Jackson, H. H. T. 1951. The Clever Coyote. Stackpole Publications, Harrisburg, Pennsylvania and The Wildlife Management Institute, Washington, D. C.

282. Young, J. K., Andelt, W. F., Terletzky, P. A., Shivik, J. A. 2006**.** A comparison of coyote ecology after 25 years: 1978 versus 2003. Canadian Journal of Zoology 84:573-582.

283. Méndez, J. V. 2012. Variación estacional en la dieta y traslape en el nicho alimenticio entre el coyote (*Canis latrans* Say, 1823) y la zorra gris (*Urocyon cinereoargenteus* Schreber, 1775) en una zona rural de la porción sur del altiplano Mexicano., Licenciado en biologia tesis, Universidad de Guadalajara, Guadalajara, Mexico.

284. Hinton, J. W., Rountree, K., Chamberlain, M. J. 2021. Diet of coyotes on the Tensas River National Wildlife Refuge during the white-tailed deer pre-fawning and fawning seasons. Southeastern Naturalist 20:245-258.
