# Supplementary material for: Diet selection in the Coyote Canis latrans
Source: J Mammal. 2023 Nov 4;104(6):1338–52. doi: 10.1093/jmammal/gyad094 (PMC10697429; doi:10.1093/jmammal/gyad094)
Supplement: gyad094_suppl_Supplementary_Data_SD2 [file gyad094_suppl_supplementary_data_sd2.docx]

**Supplementary Materials Table S2. Summary results of coyote prey preferences.**

| Species | Scientific name | Pref/Avoid | Jacobs index | n | Body mass (kg) | Kills | t | d.f. | p |
| --- | --- | --- | --- | --- | --- | --- | --- | --- | --- |
| Beaver, American | *Castor canadensis* | *~* | -0.77 +- 0.14 | 2 | 21.82 | 0.03 +- 0.04 | -5.58 | 1 | 0.110 |
| Birds | Aves | *~* | -0.09 +- 0.20 | 6 | 0.2 | 0.05 +- 0.1 | -0.45 | 5 | 0.670 |
| Chipmunk, least | *Neotamias minimus* | *-* | -0.75 +- 0.06 | 7 | 0.14 | 0.02 +- 0.01 | -12.72 | 6 | 0.000 |
| Chipmunks | Sciuridae | *~* | -0.49 +- 0.18 | 7 | 0.11 | 0.01 +- 0.01 | -2.79 | 6 | 0.030 |
| Coyote | *Canis latrans* | *-* | -0.74 +- 0.04 | 5 | 13.41 | 0.02 +- 0.01 | -17.9 | 4 | 0.000 |
| Deer, mule | *Odocoileus hemionus* | *~* | 0.14 +- 0.20 | 18 | 54.21 | 0.08 +- 0.03 | 0.7 | 17 | 0.490 |
| Deer, white-tailed | *Odocoileus virginianus* | *+* | 0.47 +- 0.10 | 38 | 55.51 | 0.16 +- 0.03 | 4.71 | 37 | 0.000 |
| Elk | *Cervus canadensis* | *~* | 0.23 +- 0.39 | 2 | 131.25 | 0.16 +- 0.12 | 0.58 | 1 | 0.660 |
| Gopher, northern pocket | *Thomomys talpoides* | *-* | -0.56 +- 0.15 | 8 | 0.13 | 0.11 +- 0.04 | -3.86 | 7 | 0.010 |
| Grasshopper | Caelifera | *~* | -0.05 +- 0.55 | 3 | 0.01 | 0.06 +- 0.05 | -0.09 | 2 | 0.930 |
| Grouse, spruce | *Falcipennis canadensis* | *-* | -0.85 +- 0.06 | 5 | 0.47 | 0.03 +- 0.01 | -13.85 | 4 | 0.000 |
| Hare, showshoe | *Lepus americanus* | *~* | 0.2 +- 0.13 | 30 | 1.71 | 0.2 +- 0.04 | 1.52 | 29 | 0.140 |
| Invertebrates | Invertebrata | *~ -* | -0.71 +- 0.22 | 4 | 0 | 0.07 +- 0.05 | -3.22 | 3 | 0.050 |
| Jackrabbit | *Lepus spp.* | *+* | 0.96 +- 0.01 | 4 | 2.45 | 0.1 +- 0.08 | 75.76 | 3 | 0.000 |
| Jackrabbit, black-tailed | *Lepus californicus* | *~* | 0.2 +- 0.11 | 29 | 2.42 | 0.15 +- 0.04 | 1.84 | 28 | 0.080 |
| Lemming, southern bog | *Synaptomys cooperi* | *~* | 0.62 +- 0.20 | 2 | 0.03 | 0.03 +- 0.08 | 3.12 | 1 | 0.200 |
| Marmot, yellow-bellied | *Marmota flaviventris* | *~* | -0.43 +- 0.29 | 2 | 3.35 | 0.03 +- 0.02 | -1.47 | 1 | 0.380 |
| Moose | *Alces alces* | *~* | 0.04 +- 0.20 | 7 | 357 | 0.11 +- 0.06 | 0.19 | 6 | 0.860 |
| Mouse, California deer | *Peromyscus californicus* | *-* | -0.91 +- 0.09 | 4 | 0.02 | 0 +- 0 | -9.92 | 3 | 0.000 |
| Mouse, deer | *Peromyscus spp.* | *-* | -0.72 +- 0.06 | 64 | 0.02 | 0.03 +- 0.01 | -11.86 | 63 | 0.000 |
| Mouse, fulvous harvest | *Reithrodontomys fulvescens* | *~* | -0.39 +- 0.58 | 2 | 0.01 | 0.01 +- 0.01 | -0.67 | 1 | 0.620 |
| Mouse, Great Basin pocket | *Perognathus parvus* | *~* | -0.1 +- 0.20 | 12 | 0.02 | 0.03 +- 0.01 | -0.5 | 11 | 0.630 |
| Mouse, harvest | *Reithrodontomys spp.* | *-* | -0.83 +- 0.05 | 8 | 0.01 | 0.03 +- 0.02 | -18.42 | 7 | 0.000 |
| Mouse, hispid pocket | *Chaetodipus hispidus* | *~* | -0.37 +- 0.30 | 6 | 0.03 | 0.01 +- 0 | -1.24 | 5 | 0.270 |
| Mouse, northern grasshopper | *Onychomys leucogaster* | *~* | -0.63 +- 0.27 | 4 | 0.03 | 0.01 +- 0.01 | -2.32 | 3 | 0.100 |
| Mouse, pinyon deer | *Peromyscus truei* | *-* | -0.66 +- 0.18 | 4 | 0.03 | 0.01 +- 0 | -3.66 | 3 | 0.040 |
| Mouse, pocket | *Chaetodipus spp.* | *~* | -0.24 +- 0.16 | 10 | 0.03 | 0.04 +- 0.02 | -1.52 | 9 | 0.160 |
| Mouse, southern marsh harvest | *Reithrodontomys megalotis* | *-* | -0.79 +- 0.21 | 4 | 0.01 | 0.01 +- 0.01 | -3.7 | 3 | 0.030 |
| Mouse, western harvest | *Reithrodontomys megalotis* | *~* | 0.14 +- 0.20 | 10 | 0.01 | 0.03 +- 0.02 | 0.72 | 9 | 0.490 |
| Mouse, western jumping | *Zapus princeps* | *~* | -0.65 +- 0.35 | 2 | 0.02 | 0.03 +- 0.03 | -1.85 | 1 | 0.320 |
| Mouse, white-footed | *Peromyscus leucopus* | *-* | -0.56 +- 0.17 | 6 | 0.02 | 0.05 +- 0.02 | -3.33 | 5 | 0.020 |
| Muskrat | *Ondatra zibethicus* | *-* | -0.83 +- 0.08 | 8 | 1.07 | 0.02 +- 0.02 | -9.86 | 7 | 0.000 |
| Opossum, Virginia | *Didelphis virginiana* | *~ -* | -0.92 +- 0.08 | 2 | 2.2 | 0.01 +- 0.01 | -12.19 | 1 | 0.050 |
| Pheasant, ring-necked | *Phasianus colchicus* | *~* | 0.34 +- 0.45 | 4 | 1.12 | 0.02 +- 0.02 | 0.75 | 3 | 0.510 |
| Pronghorn | *Antilocapra americana* | *~* | -0.12 +- 0.35 | 4 | 46.08 | 0.03 +- 0.03 | -0.34 | 3 | 0.760 |
| Rabbit, desert cottontail | *Sylvilagus audubonii* | *~ +* | 0.35 +- 0.16 | 14 | 0.89 | 0.08 +- 0.02 | 2.11 | 13 | 0.050 |
| Rabbit, eastern cottontail | *Sylvilagus floridanus* | *+* | 0.36 +- 0.09 | 15 | 1.17 | 0.13 +- 0.03 | 4.21 | 14 | 0.000 |
| Rabbit, mountain cottontail | *Sylvilagus nuttallii* | *~ +* | 0.67 +- 0.21 | 3 | 0.76 | 0.06 +- 0.04 | 3.23 | 2 | 0.080 |
| Raccoon, northern | *Procyon lotor* | *-* | -0.94 +- 0.02 | 2 | 6.55 | 0.02 +- 0.04 | -56.8 | 1 | 0.010 |
| Rat, hispid cotton | *Sigmodon hispidus* | *~* | 0.02 +- 0.10 | 27 | 0.09 | 0.81 +- 1.80 | 0.23 | 26 | 0.820 |
| Rat, Ord's kangaroo | *Dipodomys ordii* | *-* | -0.48 +- 0.10 | 11 | 0.06 | 0.04 +- 0.01 | -4.67 | 10 | 0.000 |
| Sheep | *Ovis aries* | *~* | -0.02 +- 0.13 | 6 | 70 | 0.04 +- 0.04 | -0.13 | 5 | 0.900 |
| Sheep, Dall | *Ovis dalli* | *+* | 0.67 +- 0 | 3 | 55.65 | 0.06 +- 0.01 | 229.15 | 2 | 0.000 |
| Skunk, striped | *Mephitis mephitis* | *~* | -0.41 +- 0.59 | 2 | 2.09 | 0.02 +- 0.03 | -0.7 | 1 | 0.610 |
| Small mammals | *Mammalia* | *-* | -0.55 +- 0.15 | 15 | 0.03 | 0.43 +- 0.9 | -3.64 | 14 | 0.000 |
| Squirrel, American red | *Tamiasciurus hudsonicus* | *-* | -0.82 +- 0.04 | 8 | 0.2 | 0.02 +- 0.01 | -18.9 | 7 | 0.000 |
| Squirrel, fox | *Sciurus niger* | *-* | -0.98 +- 0.02 | 2 | 0.7 | 0 +- 0 | -42.69 | 1 | 0.010 |
| Squirrel, golden-mantled ground | *Callospermophilus lateralis* | *~* | 0.04 +- 0.29 | 3 | 0.19 | 0.03 +- 0.03 | 0.15 | 2 | 0.900 |
| Squirrel, ground | Sciuridae | *~* | -0.48 +- 0.31 | 4 | 0.27 | 0.06 +- 0.07 | -1.57 | 3 | 0.220 |
| Squirrel, Townsend's ground | *Urocitellus townsendii* | *~* | -0.24 +- 0.14 | 3 | 0.41 | 0.04 +- 0.02 | -1.76 | 2 | 0.220 |
| Squirrel, Uinta ground | *Urocitellus armatus* | *~* | 0.12 +- 0.50 | 4 | 0.31 | 0.06 +- 0.03 | 0.25 | 3 | 0.820 |
| Vole, California | *Microtus californicus* | *+* | 0.25 +- 0.05 | 4 | 0.06 | 0.08 +- 0.05 | 5.38 | 3 | 0.010 |
| Vole, meadow | *Microtus pennsylvanicus* | *-* | -0.82 +- 0.13 | 3 | 0.04 | 0.08 +- 0.05 | -6.3 | 2 | 0.020 |
| Vole, prairie | *Microtus ochrogaster* | *~* | -0.13 +- 0.18 | 11 | 0.04 | 0.1 +- 0.02 | -0.74 | 10 | 0.470 |
| Vole, southern red-backed | *Myodes gapperi* | *-* | -0.87 +- 0.10 | 6 | 0.02 | 0.03 +- 0.02 | -8.43 | 5 | 0.000 |
| Waterfowl | Anatidae | *-* | -0.97 +- 0.02 | 3 | 1 | 0.04 +- 0.04 | -58.55 | 2 | 0.000 |
| Woodchuck/groundhog | *Marmota monax* | *~* | -0.61 +- 0.16 | 2 | 3.81 | 0.05 +- 0.05 | -3.84 | 1 | 0.160 |
| Woodrat, desert | *Neotoma lepida* | *~* | -0.52 +- 0.38 | 2 | 0.16 | 0.05 +- 0.06 | -1.35 | 1 | 0.410 |
| Woodrat, eastern | *Neotoma floridana* | *~* | 0.47 +- 0.05 | 2 | 0.24 | 0.02 +- 0.01 | 9.98 | 1 | 0.060 |
| Woodrat, southern plains | *Neotoma micropus* | *~* | -0.07 +- 0.08 | 27 | 0.24 | 0.06 +- 0.01 | -0.86 | 26 | 0.400 |
